# Supplementary material for: Design, Synthesis and Biological Evaluation of Pyrazolopyrimidine Derivatives as Aryl Hydrocarbon Receptor Antagonists for Colorectal Cancer Immunotherapy
Source: Pharmaceutics. 2025 Oct 21;17(10):1359. doi: 10.3390/pharmaceutics17101359 (PMC12567141; doi:10.3390/pharmaceutics17101359)

pharmaceutics

Supplementary Materials: Design, Synthesis and Biological Evaluation of Pyrazolopyrimidine Derivatives as Aryl Hydrocarbon Receptor Antagonists for Colorectal Cancer Immunotherapy

Byeong Wook Choi [1,†], Jae-Eon Lee [2,†], Da Bin Jeon [3], Pyeongkeun Kim [1], Gwi Bin Lee [1], Saravanan Parameswaran [4], Ji Yun Jang [3], Gopalakrishnan Chandrasekaran [3], So Yeon Jeong [2], Geumi Park [2], Kyoung-jin Min [5], Heegyum Moon [5], Jihyeon Yoon [1], Yerim Heo [1], Donggun Kim [1], Se Hwan Ahn [1], You Jeong Choi [1], Seong Soon Kim [6], Jung Yoon Yang [6], Myung Ae Bae [6], Yong Hyun Jeon [2], Seok-Yong Choi [3,*] and Jin Hee Ahn [1,7,*]

 https://doi.org/10.3390/pharmaceutics17101359

List of contents

Figure S1-11, Table S1 .... **3**

Synthesis of biological testing compound **15**

HPLC purity analysis **24**

^{1}H NMR and ^{13}C NMR spectra **34**

HRMS data **61**

Supporting information

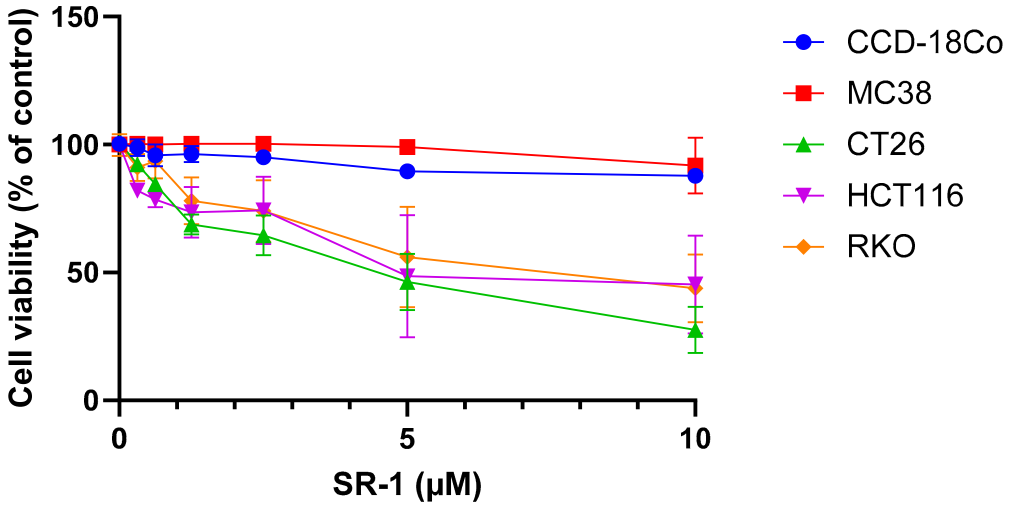

Figure S1. The effects of SR1 on the proliferation of normal and colorectal cancer cells. (A) Cell viability of normal colon cells (CCD-18Co) and colorectal cancer cells (MC38, CT26, HCT116, and RKO) following 24 h treatment with SR1. Cell viability was assessed using the Cell Counting Kit-8 (CCK-8) assay. Each experiment was conducted in duplicate.

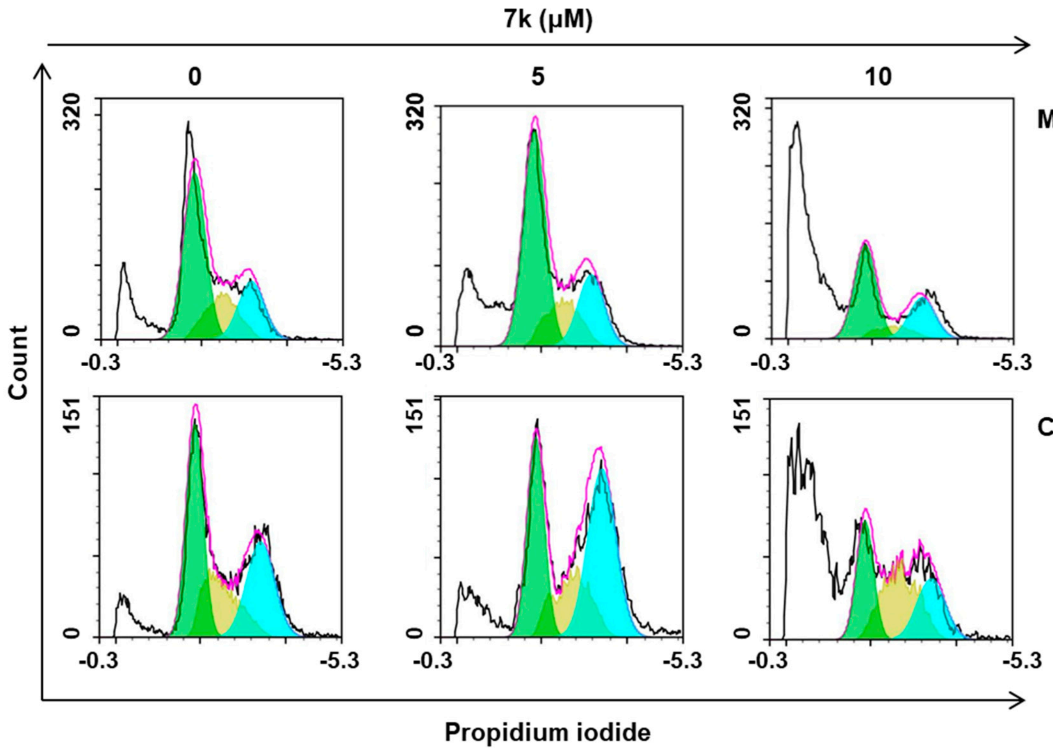

Figure S2. Cell cycle analysis in **7k**-induced colorectal cancer cells. MC38 and CT26 cells were treated with **7k** for 24h, and apoptosis analysis was conducted.

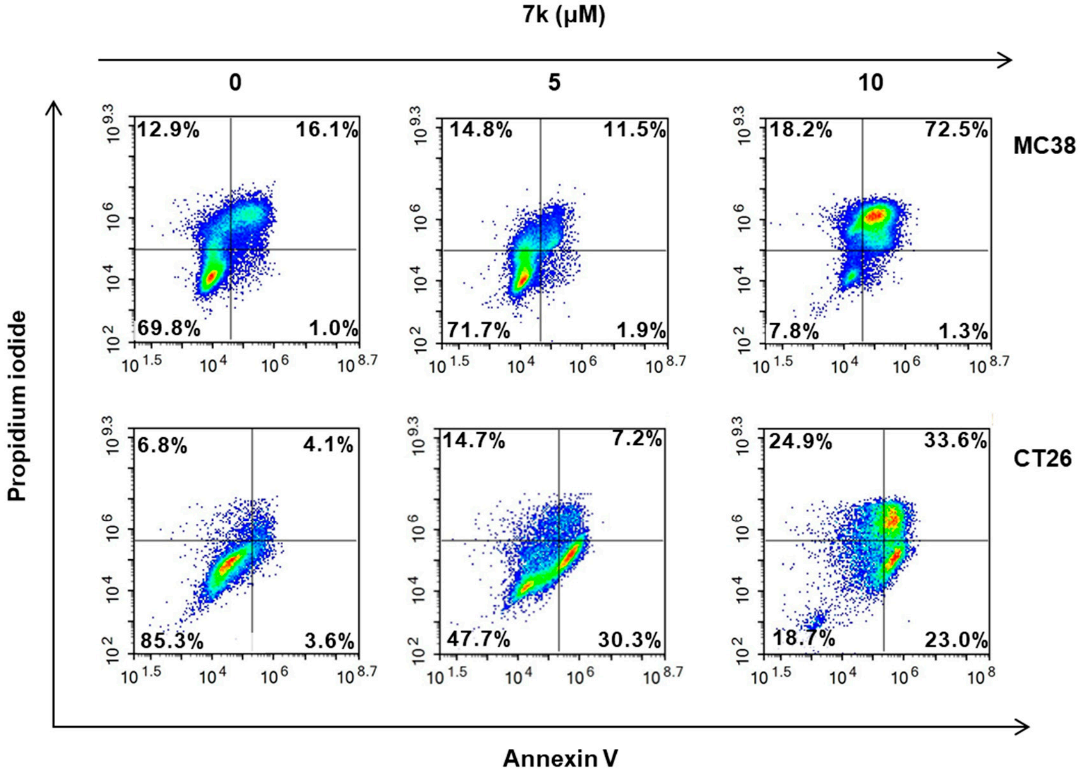

Figure S3. Apoptosis analysis with annexin V and propidium iodide in **7k**-induced colorectal cancer cells. MC38 and CT26 cells were treated with **7k** for 24h, and apoptosis analysis was conducted.

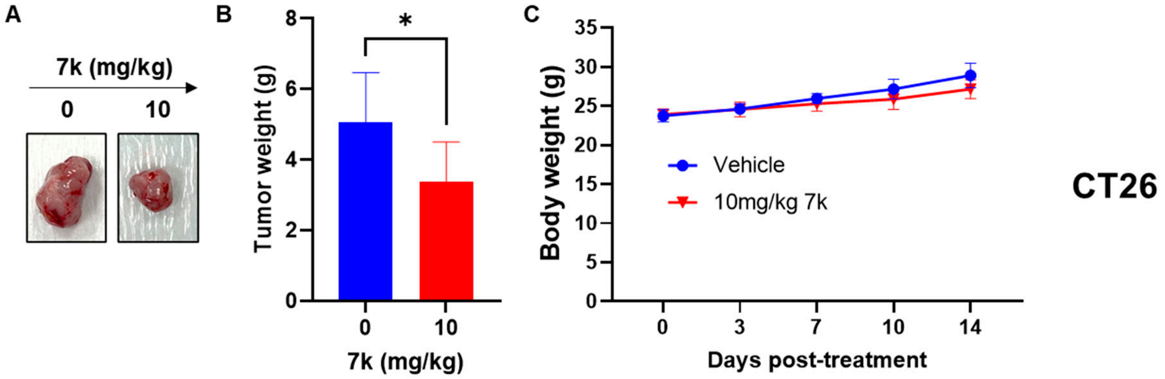

Figure S4. (A) Photo of excised tumors, (B) tumor weight, and (C) body weight in CT26 tumor bearing mice treated with 7k. Data are presented as the mean ± SD. ns, not significant. *, P < 0.05, compared to vehicle-treated cells.

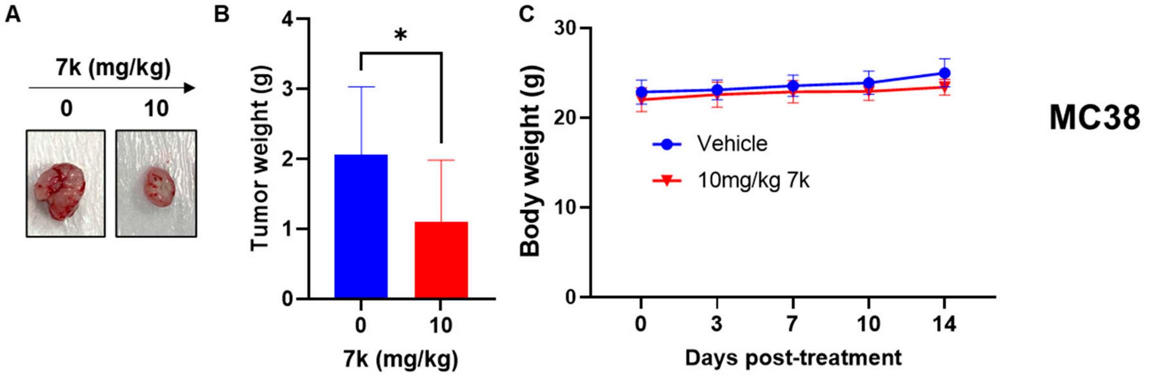

Figure S5. (A) Photo of excised tumors, (B) tumor weight, and (C) body weight in MC38 tumor bearing mice treated with 7k. Data are presented as the mean ± SD. ns, not significant. *, P < 0.05, compared to vehicle-treated cells.

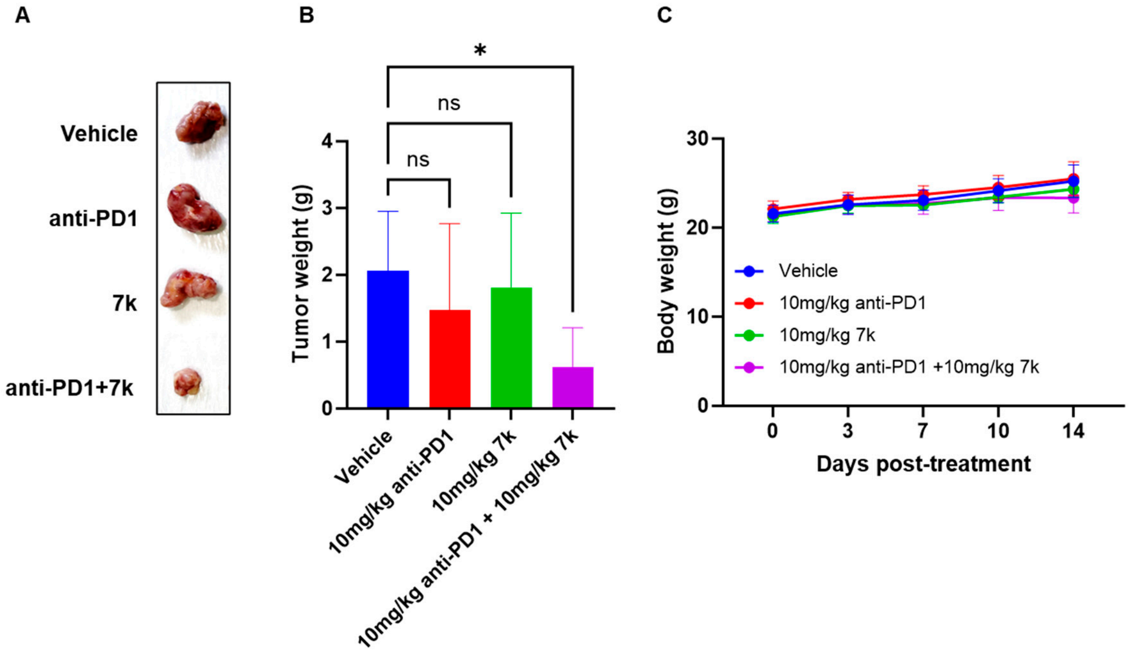

Figure S6. (A) Photo of excised tumors, (B) tumor weight, and (C) body weig[illegible] bearing mice treated with single agent or combination. Data are presented as the mean ± SD. ns, not significant. *, P < 0.05, compared to vehicle-treated cells.

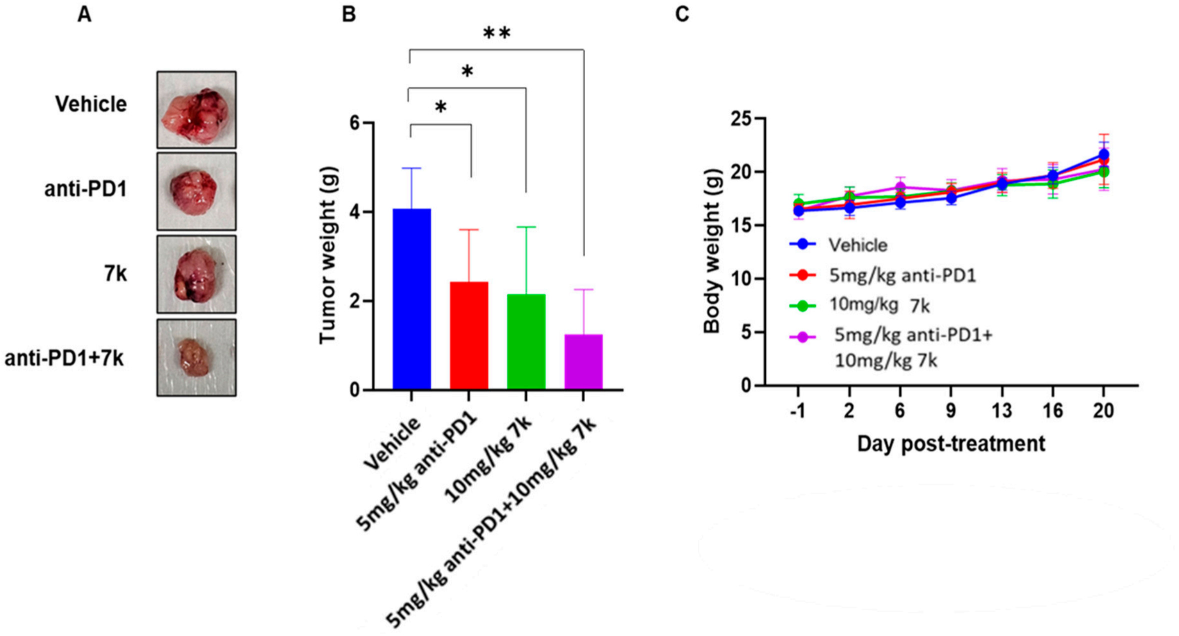

Figure S7. (A) Photo of excised tumors, (B) tumor weight, and (C) body weight in MC38 tumor bearing mice treated with single agent or combination.

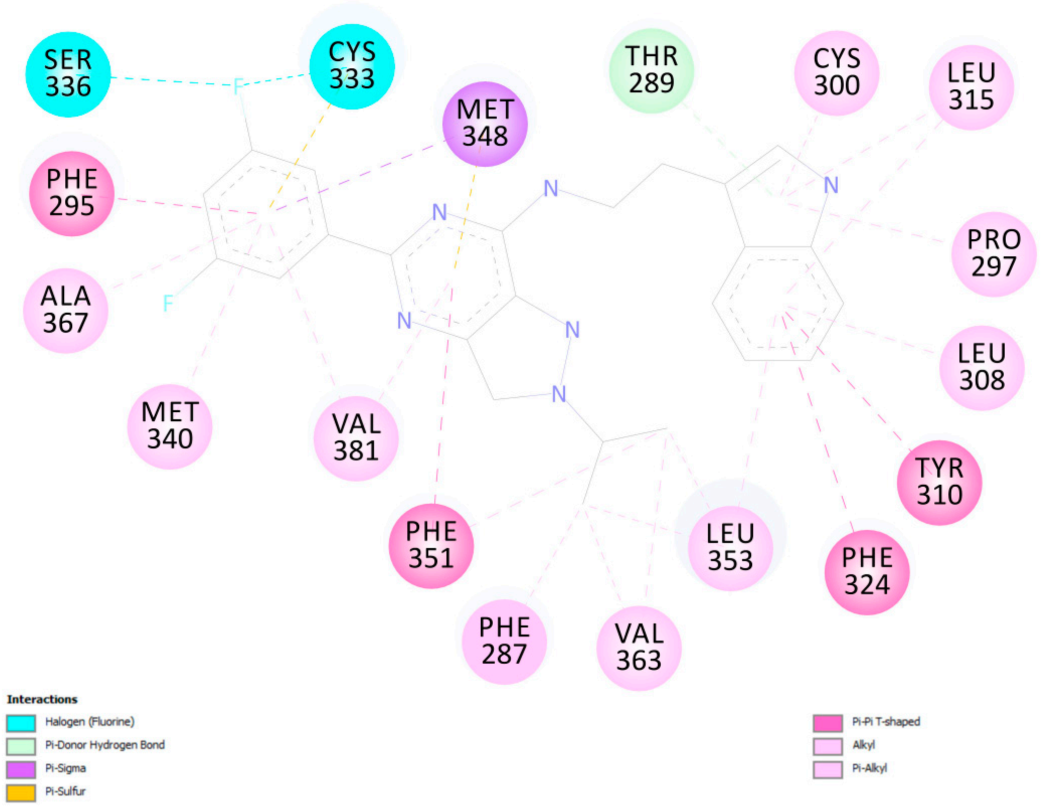

Figure S8. The intermolecular interactions of synthesized compound **7k** with AhR.

**Interaction Name**	**Distance**	**Interaction Type**	**Interaction from Atom**	**From Chemistry**	**Interaction from Atom**	**To Chemistry**
**X:CYS333:O - d:***1:F**	**3.29524**	**Halogen (Fluorine)**	**X:CYS333:O**	**Halogen Acceptor**	**d:***1:F**	**Halogen**
**X:SER336:C - d:***1:F**	**3.26572**	**Halogen (Fluorine)**	**X:SER336:C**	**Halogen Acceptor**	**d:***1:F**	**Halogen**
**X:THR289:HG1 - d:***1**	**2.70564**	**Pi-Donor Hydrogen Bond**	**X:THR289:HG1**	**H-Donor**	**d:***1**	**Pi-Orbitals**
**X:MET348:CE - d:***1**	**3.2235**	**Pi-Sigma**	**X:MET348:CE**	**C-H**	**d:***1**	**Pi-Orbitals**

**X:CYS333 :SG - d:***1**	**5.959 82**	**Pi-Sulfur**	**X:CYS333 :SG**	**Sulfur**	**d:***1**	**Pi-Orbit-als**
**X:MET34 8:SD - d:***1**	**5.052 27**	**Pi-Sulfur**	**X:MET34 8:SD**	**Sulfur**	**d:***1**	**Pi-Orbit-als**
**X:MET34 8:SD - d:***1**	**3.609 34**	**Pi-Sulfur**	**X:MET34 8:SD**	**Sulfur**	**d:***1**	**Pi-Orbit-als**
**X:PHE29 5 - d:***1**	**4.174 03**	**Pi-Pi T-shaped**	**X:PHE29 5**	**Pi-Orbit-als**	**d:***1**	**Pi-Orbit-als**
**X:TYR31 0 - d:***1**	**4.895 27**	**Pi-Pi T-shaped**	**X:TYR31 0**	**Pi-Orbit-als**	**d:***1**	**Pi-Orbit-als**
**X:PHE32 4 - d:***1**	**5.629 9**	**Pi-Pi T-shaped**	**X:PHE32 4**	**Pi-Orbit-als**	**d:***1**	**Pi-Orbit-als**

**X:PHE351 - d:***1**	**5.76024**	**Pi-Pi T-shaped**	**X:PHE351**	**Pi-Orbitals**	**d:***1**	**Pi-Orbitals**
**d:***1:C - X:LEU353**	**3.47598**	**Alkyl**	**d:***1:C**	**Alkyl**	**X:LEU353**	**Alkyl**
**d:***1:C - X:VAL363**	**3.39309**	**Alkyl**	**d:***1:C**	**Alkyl**	**X:VAL363**	**Alkyl**
**d:***1:C - X:LEU353**	**3.49463**	**Alkyl**	**d:***1:C**	**Alkyl**	**X:LEU353**	**Alkyl**
**d:***1:C - X:VAL363**	**3.25995**	**Alkyl**	**d:***1:C**	**Alkyl**	**X:VAL363**	**Alkyl**
**X:PHE287 - d:***1:C**	**5.38563**	**Pi-Alkyl**	**X:PHE287**	**Pi-Orbitals**	**d:***1:C**	**Alkyl**
**X:PHE351 - d:***1:C**	**4.24864**	**Pi-Alkyl**	**X:PHE351**	**Pi-Orbitals**	**d:***1:C**	**Alkyl**

**d:***1 - X:LEU308**	**5.34011**	**Pi-Alkyl**	**d:***1**	**Pi-Orbit-als**	**X:LEU308**	**Alkyl**
**d:***1 - X:LEU315**	**5.1613**	**Pi-Alkyl**	**d:***1**	**Pi-Orbit-als**	**X:LEU315**	**Alkyl**
**d:***1 - X:LEU353**	**5.49898**	**Pi-Alkyl**	**d:***1**	**Pi-Orbit-als**	**X:LEU353**	**Alkyl**
**d:***1 - X:PRO297**	**5.25329**	**Pi-Alkyl**	**d:***1**	**Pi-Orbit-als**	**X:PRO297**	**Alkyl**
**d:***1 - X:CYS300**	**5.33241**	**Pi-Alkyl**	**d:***1**	**Pi-Orbit-als**	**X:CYS300**	**Alkyl**
**d:***1 - X:LEU315**	**4.4408**	**Pi-Alkyl**	**d:***1**	**Pi-Orbit-als**	**X:LEU315**	**Alkyl**
**d:***1 - X:VAL381**	**4.87317**	**Pi-Alkyl**	**d:***1**	**Pi-Orbit-als**	**X:VAL381**	**Alkyl**

**d:***1 - X:MET340**	**4.92865**	**Pi-Alkyl**	**d:***1**	**Pi-Orbitals**	**X:MET340**	**Alkyl**
**d:***1 - X:ALA367**	**4.92041**	**Pi-Alkyl**	**d:***1**	**Pi-Orbitals**	**X:ALA367**	**Alkyl**
**d:***1 - X:VAL381**	**5.25897**	**Pi-Alkyl**	**d:***1**	**Pi-Orbitals**	**X:VAL381**	**Alkyl**

Table S1. The intermolecular interactions of synthesized compounds with AhR.

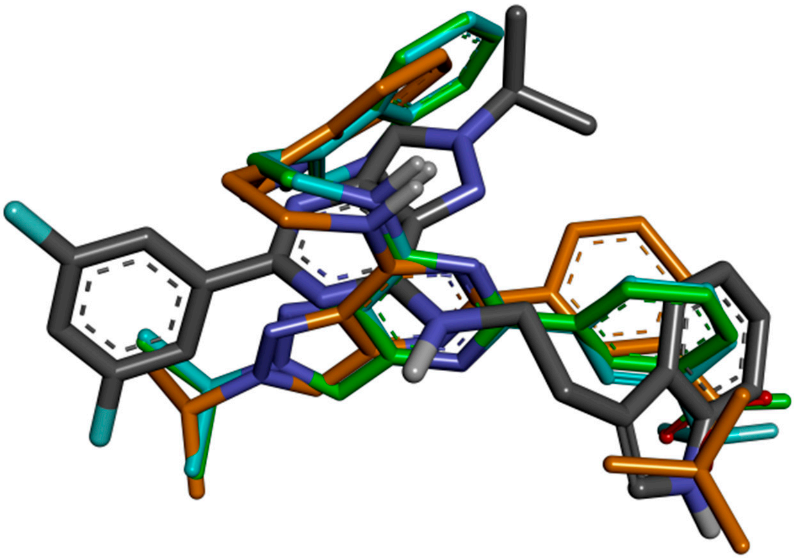

Figure S9. The 3D overlay of synthesized compounds **7e**, **7f**, and **7i** with compound **7k** revealed that the binding pose of compounds **7e**, **7f**, and **7i** was different from that of compound **7k**. The binding pose of compounds were represented by sticks with CPK colors while the carbon atoms of compounds **7e**, **7f**, and **7i** were colored as green, cyan, and orange.

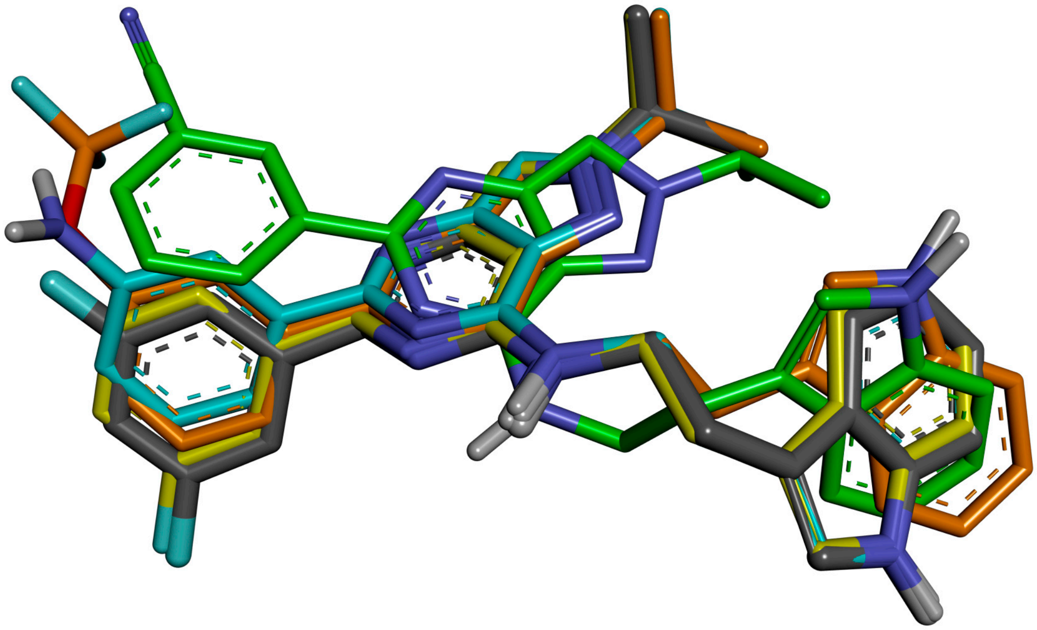

Figure S10. The 3D overlay of synthesized compounds **7c**, **7g**, **7h**, and **7j** with compound **7k** revealed that their overall binding modes were similar. The binding poses of compounds **7g** and **7j** share a similar binding pose with compound **7k**, while a slight deviation is observed in the compounds **7c** and **7h**. The binding pose of compounds were represented by sticks with CPK colors while the carbon atoms of compounds **7c**, **7g**, **7h** and **7j** were colored as green, cyan, orange, and yellow.

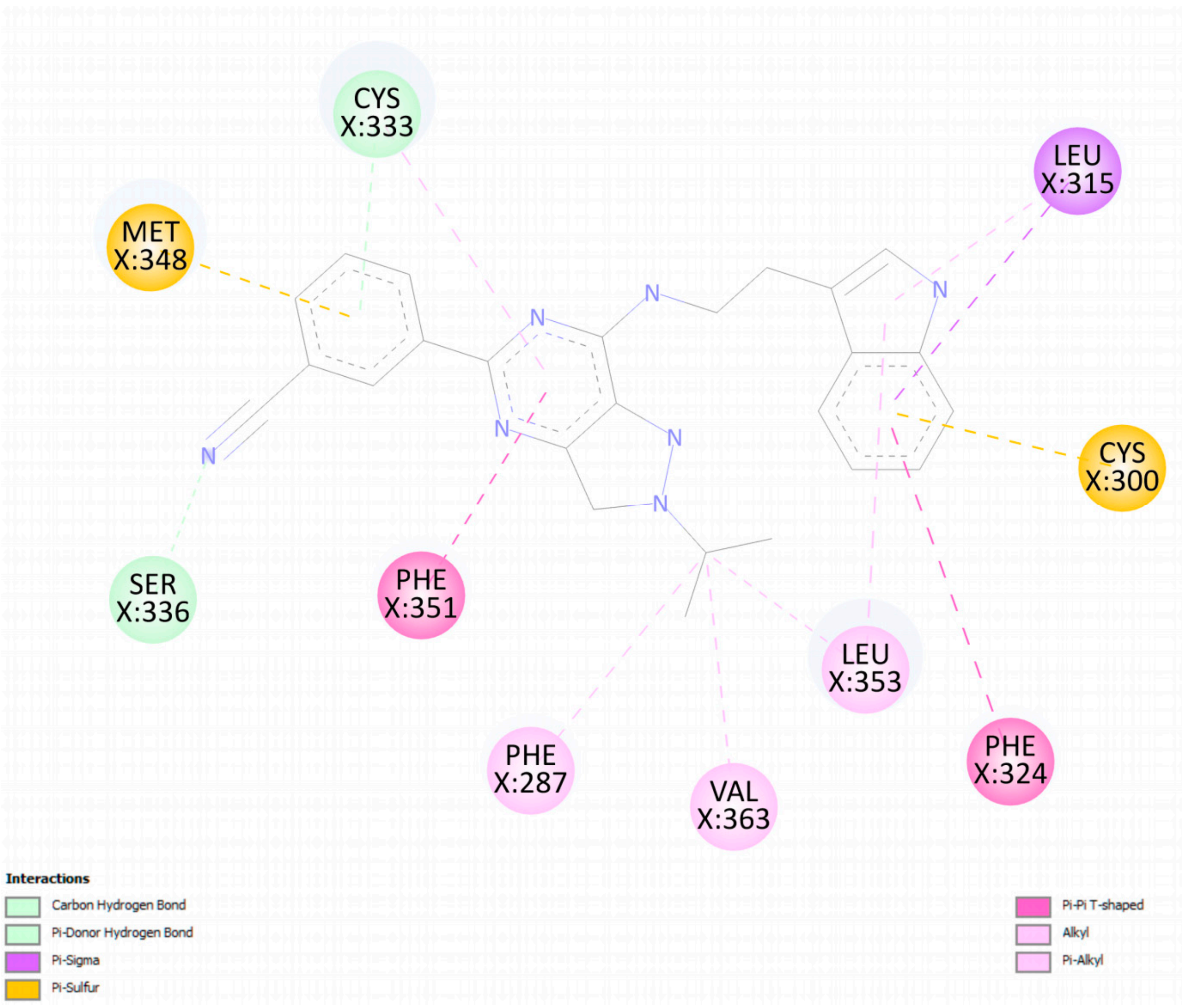

Figure S11. The intermolecular interactions of synthesized compound **7c** with AhR.

Experimental procedures

General

All solvents and chemicals were used as purchased without further purification. All the reported yields are isolated yields after column chromatography or crystallization. ^{1}H NMR spectra and ^{13}C spectra were recorded on a JEOL JNM-ECS400 spectrometer at 400 MHz for ^{1}H NMR and 100 MHz for ^{13}C NMR, respectively. The chemical shift (δ) is expressed in ppm relative to tetramethylsilane (TMS) as an internal standard, and $CDCl_3$, MeOH-*d*$_4$ and DMSO-*d*$_6$ were used as solvents. Multiplicity of peaks is expressed as s (singlet), d (doublet), t (triplet), q (quartet), dd (doublet of doublets), td (triplet of doublets), qd (quartet of doublets), dt (doublet of triplets), and m (multiplet). HRMS data were obtained by a JMS 700 (JEOL, Japan). Melting points were determined on a Melting Point M-560, purchased from Buchi. Optical rotations were measured on a P-2000 polarimeter, purchased from Jasco. High-performance liquid chromatography (HPLC) analyses were performed with a Waters Agilent HPLC system equipped with a PDA detector and an Agilent SB-C18 column (1.8 μm, 2.1 × 50 mm). The mobile phase consisted of buffer A (ultrapure H2O containing 0.1% trifluoroacetic acid) and buffer B (chromatographic grade CH3CN) for method A and buffer C (chromatographic grade MeOH) for method B was applied at a flow rate of 0.3 mL/min.Method

Time (min)	Water (%)	CH_3CN (%)
0	95	5
15	0	100

General procedure of 7a-m

N-(2-(1H-indol-3-yl)ethyl)-2-isopropyl-5-phenyl-2H-pyrazolo[4,3-d]pyrimidin-7-amine (7a)

Step 1. Commercially available 4-nitro-1*H*-pyrazole-3-carboxylic acid (1 g, 6.37 mmol) was dissolved in 100 mL of methanol and 1 mL of sulfuric acid was added. The mixture was heated under reflux for 12 hours. After completion of the reaction, the methanol was removed under vacuum. pH of the solution was adjusted to ~7 by aq.NaOH. Afterwards, the mixture was extracted with water and EA. The combined organic layer was dried over anhydrous Na_2SO_4, filtered, and evaporated. The dried extract was concentrated under reduced pressure and purified on silica gel column chromatography (0-100% EA/n-Hexane) to obtain methyl 4-nitro-1*H*-pyrazole-3-carboxylate as a white solid (1.05 g, 96%). ^{1}H-NMR (400 MHz, DMSO-*d*$_6$) δ 8.52 (s, 1H), 3.20 (s, 3H), 1.44 (d, J = 6.9 Hz, 6H).

Step 2. Methyl 4-nitro-1*H*-pyrazole-3-carboxylate (3 g, 17.53 mmol) was dissolved in 10 mL DMF. 2-Bromopropane (3.23 g, 26.3 mmol) and potassium carbonate (3.64 g, 26.3 mmol) were added. The reaction mixture was stirred under room temperature for 12 h. After the reaction mixture was cooled to room temperature, the solvent was removed under reduced pressure. The reaction mixture was extracted with ethyl acetate and water. Then the combined organic fraction was dried over anhydrous sodium sulfate, and filtered. The solvent was removed under reduced pressure. The residue was then purified by silica gel column chromatography (0-100% EA/n-Hexane) to give methyl 1-isopropyl-4-nitro-1*H*-pyrazole-3-carboxylate (2.09 g, 56%). ^{1}H-NMR (400 MHz, DMSO-*d*$_6$) δ 9.05 (s, 1H), 4.68-4.58 (m, 1H), 3.88 (s, 3H), 1.45 (d, J = 6.7 Hz, 6H).

Step 3. Methyl 1-isopropyl-4-nitro-1*H*-pyrazole-3-carboxylate (2.09 g, 9.78 mmol) was dissolved in MeOH (50 mL). 10% Pd/C (360 mg) was added and the resulting mixture was stirred for 18 h at room temperature under hydrogen atmosphere The reaction mixture was filtered through celite and the filtrate was concentrated in vacuo and obtained without further purification. Methyl 4-amino-1-isopropyl-1*H*-pyrazole-3-carboxylate (1.37 g, 76%). ^{1}H NMR (400 MHz, $CDCl_3$) δ 7.19 (s, 1H), 4.18-4.04 (m, 2H), 1.25 (d, *J* = 7.0 Hz, 6H).

Step 4. 4-Amino-1-isopropyl-1*H*-pyrazole-3-carboxylate (1.37 g, 7.48 mmol) was added urea (2.25 g, 37.39 mmol) and the mixture was heated in a sealed tube at 200 °C for 16 h. The reaction mixture cooled to room temperature and was added with water (50 mL), and precipitate was filtered to obtain compound 2-isopropyl-2,4-dihydro-5*H*-pyrazolo[4,3-*d*]pyrimidine-5,7(6*H*)-dione (661 mg, 46%) as an off-white solid which was used in the next step without further purification. ^{1}H-NMR (400 MHz, DMSO-d_6) δ 10.82 (s, 2H), 7.68 (s, 1H), 4.62 (td, *J* = 13.4, 6.7 Hz, 1H), 1.43 (d, *J* = 6.4 Hz, 6H)

Step 5. 2-Isopropyl-2,4-dihydro-5*H*-pyrazolo[4,3-*d*]pyrimidine-5,7(6*H*)-dione (120 mg, 0.62 mmol) were added $POCl_3$ (3 mL) and under ice-cold condition and the resulting mixture was stirred for 12 h at 100 °C. The reaction mixture was cooled to room temperature, concentrated in vacuo and the residue was quenched with ice. pH of the solution was adjusted to ~7 by aq.NaOH (3-4 mL) and filtered to obtain 5,7-dichloro-2-isopropyl-2*H*-pyrazolo[4,3-*d*]pyrimidine(60 mg, 42%) as yellow solid without further purification. ^{1}H-NMR (400 MHz, DMSO-d_6) δ 9.02 (s, 1H), 5.05-4.95 (m, 1H), 1.59 (d, *J* = 6.5 Hz, 6H).

Step 6. 5,7-dichloro-2-isopropyl-2*H*-pyrazolo[4,3-*d*]pyrimidine (60 mg, 0.26 mmol), was stirred with tryptamine (62.4 mg, 0.39 mmol) in 25mL of IPA for 12 hours. Afterwards, the mixture was extracted with water and EA. The organic layer was dried with Na_2SO_4, and the dried extract was concentrated under reduced pressure. The resulting concentrate was purified on silica gel column chromatography (0-100% EA/n-Hexane) to yield *N*-(2-(1*H*-indol-3-yl)ethyl)-5-chloro-2-isopropyl-2*H*-pyrazolo[4,3-*d*]pyrimidine-7-amine (73 mg, 79%). ^{1}H-NMR (400 MHz, DMSO-d_6) δ 9.13-9.27 (1H), 8.56-8.67 (1H), 8.36 (s, 1H), 7.04 (d, *J* = 8.5 Hz, 2H), 6.68 (d, *J* = 8.5 Hz, 2H), 4.77 (s, 1H), 3.61 (d, *J* = 9.2 Hz, 2H), 2.81 (d, *J* = 7.9 Hz, 2H), 1.55-1.52 (m, 6H).

Step 7. *N*-(2-(1*H*-indol-3-yl)ethyl)-5-chloro-2-isopropyl-2*H*-pyrazolo[4,3-*d*]pyrimidine-7-amine (20 mg, 0.06 mmol) and phenylboronic acid (10.31 mg, 0.09 mmol) was stirred with 2M sodium bicarbonate solution (1 mL) and $Pd(PPh_3)_4$ (6.51 mg, 0.01 mmol) mixture in 10 mL of 1,4-dioxane at 90 °C for 3 hours. The reaction mixture was extracted with ethyl acetate and water. The combined organic layer was dried over anhydrous sodium sulfate, concentrated under vacuum. The concentrated product was purified by silica gel column chromatography (0-100% EA/n-Hexane) to yield *N*-(2-(1*H*-indol-3-yl)ethyl)-2-isopropyl-5-phenyl-2*H*-pyrazolo[4,3-*d*]pyrimidin-7-amine (9.3 mg, 42%).

HRMS (FAB) m/z calculated for $C_{24}H_{24}N_6$ $[M + H]^+$ 397.2141, found 397.2138; HPLC purity 99.0033%.

^{1}H-NMR (400 MHz, DMSO-d_6) δ 10.79 (s, 1H), 8.39 (d, *J* = 8.9 Hz, 3H), 8.16 (t, *J* = 5.6 Hz, 1H), 7.72-7.61 (m, 1H), 7.44-7.36 (m, 3H), 7.32 (d, *J* = 8.2 Hz, 1H), 7.21 (d, *J* = 1.8 Hz, 1H), 7.07-7.03 (m, 1H), 6.96 (t, *J* = 7.0 Hz, 1H), 4.81-4.71 (m, 1H), 3.90 (dd, *J* = 14.5, 6.6 Hz, 2H), 3.10 (t, *J* = 7.6 Hz, 2H), 1.54-1.48 (m, 6H)

^{13}C-NMR (101 MHz, MeOH-d_4) δ 159.3, 154.4, 139.2, 138.5, 136.9, 130.3, 129.3, 128.6, 128.0, 127.8, 127.6, 122.2, 121.0, 120.7, 118.2, 112.2, 110.9, 56.0, 41.0, 25.1, 22.0

4-(7-((2-(1H-indol-3-yl)ethyl)amino)-2-isopropyl-2H-pyrazolo[4,3-d]pyrimidin-5-yl)benzonitrile (7b)

N-(2-(1*H*-indol-3-yl)ethyl)-5-chloro-2-isopropyl-2*H*-pyrazolo[4,3-*d*]pyrimidine-7-amine (25 mg, 0.07 mmol) and 4-cyano-phenylboronic acid (15.53 mg, 0.11 mmol) was

stirred with 2M sodium bicarbonate solution (1ml) and $Pd(PPh_3)_4$ (8.14 mg, 0.01 mmol) mixture in 10 mL of dioxane at 90 °C for 3 hours. The reaction mixture was poured into brine and extracted with ethyl acetate. The combined organic layer was dried over anhydrous sodium sulfate, concentrated under vacuum. The concentrated product was purified by silica gel column chromatography (0-100% EA/n-Hexane) to yield 4-(7-((2-(1*H*-indol-3-yl)ethyl)amino)-2-isopropyl-2*H*-pyrazolo[4,3-*d*]pyrimidin-5-yl)benzonitrile (10.1 mg, 34%) as a solid.

HRMS (FAB) m/z calculated for $C_{25}H_{23}N_7$ $[M + H]^+$ 422.2093, found 422.2084; HPLC purity 97.7059%.

^{1}H-NMR (400 MHz, MeOH-d_4) δ 10.15 (s, 1H), 8.38 (d, *J* = 8.4 Hz, 2H), 8.17 (s, 1H), 7.75 (d, *J* = 8.8 Hz, 2H), 7.62 (d, *J* = 7.6 Hz, 1H), 7.29 (d, *J* = 8.0 Hz, 1H), 7.07 (t, *J* = 7.1 Hz, 2H), 6.99-6.95 (m, 1H), 4.03 (t, *J* = 7.2 Hz, 2H), 3.18 (t, *J* = 7.1 Hz, 2H), 1.61 (d, *J* = 6.9 Hz, 6H)

^{13}C-NMR (101 MHz, DMSO-d_6) δ 155.9, 154.3, 144.2, 139.3, 136.8, 132.7, 130.5, 128.7, 127.9, 123.2, 123.1, 122.7, 121.5, 119.6, 118.9, 118.8, 112.4, 111.9, 56.0, 41.1, 25.4, 23.3

3-(7-((2-(1H-indol-3-yl)ethyl)amino)-2-isopropyl-2H-pyrazolo[4,3-d]pyrimidin-5-yl)benzonitrile (7c)

N-(2-(1*H*-indol-3-yl)ethyl)-5-chloro-2-isopropyl-2*H*-pyrazolo[4,3-*d*]pyrimidine-7-amine (200 mg, 0.56 mmol) and 3-cyanophenylboronic acid (124.23 mg, 0.85 mmol) was stirred with 2M sodium bicarbonate solution (1 mL) and $Pd(PPh_3)_4$ (65.13 mg, 0.06 mmol) mixture in 10 mL of 1,4-dioxane at 90 °C for 3 hours. The reaction mixture was extracted with ethyl acetate and water. The combined organic layer was dried over anhydrous sodium sulfate, concentrated under vacuum. The concentrated product was purified by silica gel column chromatography (0-100% EA/n-Hexane) to yield 3-(7-((2-(1*H*-indol-3-yl)ethyl)amino)-2-isopropyl-2*H*-pyrazolo[4,3-*d*]pyrimidin-5-yl)benzonitrile (157.2 mg, 66%). HRMS (FAB) m/z calculated for $C_{25}H_{23}N_7$ $[M + H]^+$ 422.2093, found 422.2089; HPLC purity 99.3088%.

1H-NMR (400 MHz, DMSO-d_6) δ 10.80 (s, 1H), 8.64 (t, J = 7.7 Hz, 2H), 8.47 (s, 1H), 8.34 (d, J = 6.0 Hz, 1H), 7.87 (d, J = 7.7 Hz, 1H), 7.66-7.61 (m, 2H), 7.30 (d, J = 8.0 Hz, 1H), 7.20 (d, J = 2.2 Hz, 1H), 7.05-6.96 (m, 2H), 4.78 (t, J = 6.7 Hz, 1H), 3.89 (dd, J = 15.3, 6.7 Hz, 2H), 3.08 (t, J = 7.8 Hz, 2H), 1.53 (d, J = 6.6 Hz, 6H)

^{13}C-NMR (101 MHz, MeOH-d_4) δ 156.7, 154.4, 140.6, 138.4, 136.9, 132.3, 132.2, 131.3, 130.3, 129.0, 127.5, 122.2, 121.1, 121.0, 118.6, 118.4, 118.1, 112.1, 111.9, 110.9, 56.1, 41.0, 25.2, 22.0

1-(3-(7-((2-(1H-indol-3-yl)ethyl)amino)-2-isopropyl-2H-pyrazolo[4,3-d]pyrimidin-5-yl)phenyl)ethan-1-one (7d)

N-(2-(1*H*-indol-3-yl)ethyl)-5-chloro-2-isopropyl-2*H*-pyrazolo[4,3-*d*]pyrimidine-7-amine (20 mg, 0.06 mmol) and 3-acetylphenylboronic acid (13.86 mg, 0.09 mmol) was stirred with 2M sodium bicarbonate solution (1 ml) and $Pd(PPh_3)_4$ (6.51 mg, 0.01 mmol) mixture in 10 mL of 1,4-dioxane at 90 °C for 3 hours. The reaction mixture was extracted with ethyl acetate and water. The combined organic layer was dried over anhydrous sodium sulfate, concentrated under vacuum. The concentrated product was purified by silica gel column chromatography (0-100% EA/n-Hexane) to yield 1-(3-(7-((2-(1*H*-indol-3-yl)ethyl)amino)-2-isopropyl-2*H*-pyrazolo[4,3-*d*]pyrimidin-5-yl)phenyl)ethan-1-one (12 mg, 49%).

HRMS (FAB) m/z calculated for $C_{26}H_{26}N_6O$ $[M + H]^+$ 439.2246, found 439.2240; HPLC purity 95.9668%.

^{1}H-NMR (400 MHz, MeOH-d_4) δ 8.68 (t, *J* = 1.5 Hz, 1H), 8.28 (t, *J* = 7.6 Hz, 2H), 8.13-8.10 (m, 1H), 7.69 (t, *J* = 7.8 Hz, 1H), 7.58 (d, *J* = 7.6 Hz, 1H), 7.20 (d, *J* = 8.0 Hz, 1H), 7.11 (d,

J = 6.1 Hz, 1H), 6.98 (t, *J* = 7.1 Hz, 1H), 6.85 (t, *J* = 7.4 Hz, 1H), 4.93 (q, *J* = 6.7 Hz, 1H), 4.20 (t, *J* = 7.1 Hz, 2H), 3.25 (t, *J* = 7.1 Hz, 2H), 2.66 (s, 3H), 1.68-1.64 (m, 6H)

^{13}C-NMR (101 MHz, MeOH-d_4) δ 158.2, 155.8, 142.0, 140.0, 138.2, 133.8, 133.6, 132.7, 132.0, 130.4, 128.9, 124.7, 123.6, 122.3, 119.9, 119.7, 119.4, 113.5, 113.3, 112.2, 54.8, 42.4, 33.6, 26.5, 20.7

N-(2-(1H-indol-3-yl)ethyl)-2-isopropyl-5-(3-(trifluoromethyl)phenyl)-2H-pyrazolo[4,3-d]pyrimidin-7-amine (7e)

N-(2-(1*H*-indol-3-yl)ethyl)-5-chloro-2-isopropyl-2*H*-pyrazolo[4,3-*d*]pyrimidine-7-amine (30 mg, 0.09 mmol) and 3-trifluoromethylphenylboronic acid (24.13 mg, 0.13 mmol) was stirred with 2M sodium bicarbonate solution (1 mL) and Pd(PPh_3)$_4$ (9.79 mg, 0.01 mmol) mixture in 10 mL of 1,4-dioxane at 90 °C for 3 hours. The reaction mixture was extracted with ethyl acetate and water. The combined organic layer was dried over anhydrous sodium sulfate, concentrated under vacuum. The concentrated product was purified by silica gel column chromatography (0-100% EA/n-Hexane) to yield *N*-(2-(1*H*-indol-3-yl)ethyl)-2-isopropyl-5-(3-(trifluoromethyl)phenyl)-2*H*-pyrazolo[4,3-*d*]pyrimidin-7-amine (22.4 mg, 57%).

HRMS (FAB) m/z calculated for $C_{25}H_{23}F_3N_6$ [M + H]$^+$ 465.2015, found 465.2016; HPLC purity 98.6371%.

^{1}H-NMR (400 MHz, DMSO-d_6) δ 10.81 (s, 1H), 8.69 (s, 1H), 8.64 (d, *J* = 7.9 Hz, 1H), 8.46 (s, 1H), 8.31 (t, *J* = 5.8 Hz, 1H), 7.75 (d, *J* = 7.9 Hz, 1H), 7.68-7.60 (m, 2H), 7.31 (d, *J* = 7.9 Hz, 1H), 7.19 (d, *J* = 1.8 Hz, 1H), 7.04 (t, *J* = 7.2 Hz, 1H), 6.95 (t, *J* = 7.5 Hz, 1H), 4.78 (td, *J* = 13.4, 6.6 Hz, 1H), 3.90 (dd, *J* = 14.6, 6.4 Hz, 2H), 3.11 (t, *J* = 7.6 Hz, 2H), 1.53 (d, *J* = 6.7 Hz, 6H)

^{13}C-NMR (101 MHz, MeOH-d_4) δ 157.4, 154.4, 140.3, 138.5, 136.9, 131.4, 130.7, 130.4, 130.3, 130.0, 129.7, 128.7, 127.5, 125.5, 124.4, 122.2, 121.0, 121.0, 118.3, 118.1, 112.1, 110.9, 56.0, 41.1, 25.1, 22.0

N-(2-(1H-indol-3-yl)ethyl)-5-(3-aminophenyl)-2-isopropyl-2H-pyrazolo[4,3-d]pyrimidin-7-amine (7f)

N-(2-(1*H*-indol-3-yl)ethyl)-5-chloro-2-isopropyl-2*H*-pyrazolo[4,3-*d*]pyrimidine-7-amine (20 mg, 0.06 mmol) and 3-aminophenylboronic acid (11.58 mg, 0.09 mmol) was stirred with 2M sodium bicarbonate solution (1 mL) and Pd(PPh_3)$_4$ (6.51 mg, 0.01 mmol) mixture in 10 mL of 1,4-dioxane at 90 °C for 3 hours. The reaction mixture was extracted with ethyl acetate and water. The combined organic layer was dried over anhydrous sodium sulfate, concentrated under vacuum. The concentrated product was purified by silica gel column chromatography (0-10% DCM/MeOH) to yield *N*-(2-(1*H*-indol-3-yl)ethyl)-5-(3-aminophenyl)-2-isopropyl-2*H*-pyrazolo[4,3-*d*]pyrimidin-7-amine (9.3 mg, 40%).

HRMS (FAB) m/z calculated for $C_{24}H_{25}N_7$ [M + H]$^+$ 412.2250, found 412.2247; HPLC purity 99.5489%.

^{1}H-NMR (400 MHz, MeOH-d_4) δ 8.24 (s, 1H), 7.59 (d, *J* = 7.9 Hz, 1H), 7.42 (t, *J* = 1.8 Hz, 1H), 7.38-7.30 (m, 2H), 7.25 (t, *J* = 7.6 Hz, 1H), 7.09-6.99 (m, 3H), 6.92-6.88 (m, 1H), 4.16 (t, *J* = 7.2 Hz, 2H), 3.22 (t, *J* = 7.0 Hz, 2H), 1.63 (d, *J* = 6.7 Hz, 6H)

^{13}C-NMR (101 MHz, MeOH-d_4) δ 156.5, 155.5, 136.8, 132.5, 129.7, 128.8, 127.4, 126.6, 122.7, 122.5, 121.0, 120.3, 118.4, 118.3, 118.0, 117.3, 115.4, 111.4, 110.9, 57.1, 42.3, 25.0, 21.8

3-(7-((2-(1H-indol-3-yl)ethyl)amino)-2-ethyl-2H-pyrazolo[4,3-d]pyrimidin-5-yl)benzonitrile (7g)

Step 1. Methyl 4-nitro-1*H*-pyrazole-3-carboxylate (1 g, 5.84 mmol) was dissolved in DMF (10 mL). Bromoethane (955.26 mg, 8.78 mmol) and potassium carbonate (1.21 g, 8.78 mmol) were added. The reaction mixture was stirred under room temperature for 12 h.

After the reaction mixture was cooled to room temperature, the solvent was removed under reduced pressure. The reaction mixture was extracted with ethyl acetate and water. Then the combined organic fraction was dried over anhydrous sodium sulfate, and filtered. The solvent was removed under reduced pressure. The residue was then purified by silica gel column chromatography (0-100% EA/n-Hexane) to give methyl 1-ehtyl-4-nitro-1*H*-pyrazole-3-carboxylate (680 mg, 58%). ^{1}H-NMR (400 MHz, DMSO-d_6) δ 8.94 (s, 1H), 4.25 (q, *J* = 7.4 Hz, 2H), 1.55 (t, *J* = 7.2 Hz, 3H)

Step 2. Methyl 1-ethyl-4-nitro-1*H*-pyrazole-3-carboxylate (680 mg, 3.41 mmol) was dissolved in MeOH (50 mL). 10% Pd/C (120 mg) was added and the resulting mixture was stirred for 18 h at room temperature under hydrogen atmosphere The reaction mixture was filtered through celite and the filtrate was concentrated in vacuo and obtained without further purification. Methyl 4-amino-1-ethyl-1*H*-pyrazole-3-carboxylate (500.3 mg, 87%). ^{1}H-NMR (400 MHz, $CDCl_3$) δ 6.93 (s, 1H), 4.06 (q, *J* = 6.8 Hz, 2H), 3.86 (s, 3H), 1.40 (t, *J* = 7.2Hz, 3H)

Step 3. Methyl 4-amino-1-ethyl-1*H*-pyrazole-3-carboxylate (300 mg, 1.77 mmol) was added urea (532.51 mg, 8.87 mmol) and the mixture was heated in a sealed tube at 200 °C for 16 h. The reaction mixture cooled to room temperature and was added with water (50 mL), and precipitate was filtered to obtain compound 2-ethyl-2,4-dihydro-5*H*-pyrazolo[4,3-*d*]pyrimidine-5,7(6*H*)-dione (80.2 mg, 25%) as an off-white solid which was used in the next step without further purification. ^{1}H-NMR (400 MHz, DMSO-d_6) δ 10.81 (s, 2H), 7.64 (s, 1H), 4.19 (q, *J* = 7.2 Hz, 2H), 1.35 (t, *J* = 7.2 Hz, 3H)

Step 4. 2-Ethyl-2,4-dihydro-5*H*-pyrazolo[4,3-*d*]pyrimidine-5,7(6*H*)-dione (80.2 mg, 0.45 mmol) were added $POCl_3$ (5 mL) and under ice-cold condition and the resulting mixture was stirred for 12 h at 100 °C. The reaction mixture was cooled to room temperature, concentrated in vacuo and the residue was quenched with ice. pH of the solution was adjusted to ~7 by aq.NaOH (3-4 mL) and filtered to obtain 5,7-dichloro-2-ethyl-2*H*-pyrazolo[4,3-*d*]pyrimidine(72.0 mg, 75%).

^{1}H-NMR (400 MHz, DMSO-d_6) δ 8.94 (s, 1H), 4.57 (q, *J* = 7.4 Hz, 2H), 1.51 (t, *J* = 7.2 Hz, 3H)

Step 5. 5,7-Dichloro-2-ethyl-2*H*-pyrazolo[4,3-*d*]pyrimidine (40 mg, 0.18 mmol), was stirred with tryptamine (44.3 mg, 0.28 mmol) in 25 mL of IPA for 12 hours. Afterwards, the mixture was extracted with water and EA. The organic layer was dried with Na_2SO_4, and the dried extract was concentrated under reduced pressure. The resulting concentrate was purified on silica gel column chromatography (0-100% EA/n-Hexane) to yield *N*-(2-(1*H*-indol-3-yl)ethyl)-5-chloro-2-ethyl-2*H*-pyrazolo[4,3-*d*]pyrimidine-7-amine (50.2 mg, 80%).

^{1}H-NMR (400 MHz, DMSO-d_6) δ 10.78 (s, 1H), 8.76 (t, *J* = 5.8 Hz, 1H), 8.29 (s, 1H), 7.68 (d, *J* = 7.6 Hz, 1H), 7.29 (d, *J* = 7.9 Hz, 1H), 7.16 (d, *J* = 2.4 Hz, 1H), 7.05-7.01 (m, 1H), 6.94 (t, *J* = 7.0 Hz, 1H), 4.37 (q, *J* = 7.3 Hz, 2H), 3.69 (dd, *J* = 14.8, 6.3 Hz, 2H), 3.01-2.95 (m, 2H), 1.45 (t, *J* = 7.3 Hz, 3H)

Step 6. *N*-(2-(1*H*-indol-3-yl)ethyl)-5-chloro-2-ethyl-2*H*-pyrazolo[4,3-*d*]pyrimidine-7-amine (20 mg, 0.06 mmol) and 3-cyano-phenylboronic acid (12.93 mg, 0.09 mmol) was stirred with 2M sodium bicarbonate solution (1 mL) and $Pd(PPh_3)_4$ (6.78 mg, 0.01 mmol) mixture in 10 mL of 1,4-dioxane at 90 °C for 3 hours. The reaction mixture was poured into brine and extracted with ethyl acetate. The combined organic layer was dried over anhydrous sodium sulfate, concentrated under vacuum. The concentrated product was purified by silica gel column chromatography (0-100% EA/n-Hexane) to yield 3-(7-((2-(1*H*-indol-3-yl)ethyl)amino)-2-ethyl-2*H*-pyrazolo[4,3-*d*]pyrimidin-5-yl)benzonitrile (9.2 mg, 39%) as a solid.

HRMS (FAB) m/z calculated for $C_{24}H_{21}N_7$ $[M + H]^+$ 408.1937, found 408.1931; HPLC purity 98.6363%.

^{1}H-NMR (400 MHz, MeOH-d_4) δ 8.60 (d, *J* = 1.5 Hz, 1H), 8.54 (dt, *J* = 7.9, 1.4 Hz, 1H), 8.13 (s, 1H), 7.74 (dt, *J* = 7.8, 1.3 Hz, 1H), 7.65 (d, *J* = 7.6 Hz, 1H), 7.58 (t, *J* = 7.8 Hz, 1H), 7.30 (d, *J* = 8.0 Hz, 1H), 7.10-7.00 (m, 3H), 4.45 (q, *J* = 7.4 Hz, 2H), 4.08-4.01 (m, 2H), 3.19 (t, *J* = 7.2 Hz, 2H), 1.58 (t, *J* = 7.2 Hz, 3H)

^{13}C-NMR (101 MHz, MeOH-d_4) δ 156.8, 154.5, 140.6, 138.7, 136.9, 132.4, 132.2, 131.3, 130.6, 129.0, 127.5, 122.7, 122.2, 121.0, 118.6, 118.4, 118.0, 112.1, 111.9, 110.9, 48.7, 41.0, 25.2, 14.7

3-(7-((2-(1H-indol-3-yl)ethyl)amino)-2-butyl-2H-pyrazolo[4,3-d]pyrimidin-5-yl)benzonitrile (7h)

Step 1. Methyl 4-nitro-1*H*-pyrazole-3-carboxylate (1 g, 5.84 mmol) was dissolved in DMF (10 mL). 1-Bromobutane (1.2 g, 8.77 mmol) and potassium carbonate (1.21 g, 8.77 mmol) were added. The reaction mixture was stirred under room temperature for 12 h. After the reaction mixture was cooled to room temperature, the solvent was removed under reduced pressure. The reaction mixture was extracted with ethyl acetate and water. Then the combined organic fraction was dried over anhydrous sodium sulfate, and filtered. The solvent was removed under reduced pressure. The residue was then purified by silica gel column chromatography (0-100% EA/n-Hexane) to give methyl 1-butyl-4-nitro-1*H*-pyrazole-3-carboxylate (832 mg, 63%).

^{1}H-NMR (400 MHz, $CDCl_3$) δ 8.25 (s, 1H), 4.16 (t, *J* = 7.3 Hz, 2H), 2.04-1.84 (m, 2H), 1.38-1.22 (m, 2H), 0.94 (t, *J* = 7.5 Hz, 3H)

Step 2. Methyl 1-butyl-4-nitro-1*H*-pyrazole-3-carboxylate (832 mg, 3.66 mmol) was dissolved in MeOH (50 mL). 10% Pd/C (180 mg) was added and the resulting mixture was stirred for 18 h at room temperature under hydrogen atmosphere The reaction mixture was filtered through celite and the filtrate was concentrated in vacuo and obtained without further purification. Methyl 4-amino-1-butyl-1*H*-pyrazole-3-carboxylate (640.7 mg, 89%). ^{1}H-NMR (400 MHz, $CDCl_3$) δ 6.90 (s, 1H), 3.97 (t, *J* = 7.2 Hz, 2H), 3.83 (s, 3H), 1.77-1.70 (m, 2H), 1.29-1.19 (m, 2H), 0.84 (t, *J* = 7.3 Hz, 3H)

Step 3. Methyl 4-amino-1-butyl-1*H*-pyrazole-3-carboxylate (640.7 mg, 3.25 mmol) was added urea (1.07 g, 16.24 mmol) and the mixture was heated in a sealed tube at 200 °C for 16 h. The reaction mixture cooled to room temperature and was added with water (20 mL), and precipitate was filtered to obtain compound 2-butyl-2,4-dihydro-5*H*-pyrazolo[4,3-*d*]pyrimidine-5,7(6*H*)-dione (480.2 mg, 71%) as an off-white solid which was used in the next step without further purification.

^{1}H-NMR (400 MHz, DMSO-d_6) δ 10.63 (s, 2H), 7.63 (s, 1H), 4.43-4.36 (m, 2H), 1.91-1.78 (m, 2H), 1.24 (td, *J* = 14.9, 7.4 Hz, 2H), 0.87 (t, *J* = 7.5 Hz, 3H)

Step 4. 2-Butyl-2,4-dihydro-5*H*-pyrazolo[4,3-*d*]pyrimidine-5,7(6*H*)-dione (480.2 mg, 2.31 mmol) were added $POCl_3$ (5 mL) and under ice-cold condition and the resulting mixture was stirred for 12 h at 100 °C. The reaction mixture was cooled to room temperature, concentrated in vacuo and the residue was quenched with ice. pH of the solution was adjusted to ~7 by aq.NaOH (3-4 mL) and filtered to obtain 5,7-dichloro-2-butyl-2*H*-pyrazolo[4,3-*d*]pyrimidine(380 mg, 67%).

^{1}H-NMR (400 MHz, DMSO-d_6) δ 8.34 (s, 1H), 4.37 (t, *J* = 7.0 Hz, 2H), 1.91-1.84 (m, 2H), 1.28-1.19 (m, 2H), 0.90 (t, *J* = 7.5 Hz, 3H)

Step 5. 5,7-dichloro-2-butyl-2*H*-pyrazolo[4,3-*d*]pyrimidine (70 mg, 0.29 mmol), was stirred with tryptamine (68.63 mg, 0.43 mmol) in 25 mL of IPA for 12 hours. Afterwards, the mixture was extracted with water and EA. The organic layer was dried with Na_2SO_4, and the dried extract was concentrated under reduced pressure. The resulting concentrate was purified on silica gel column chromatography (0-100% EA/n-Hexane) to

yield *N*-(2-(1H-indol-3-yl)ethyl)-5-chloro-2-butyl-2*H*-pyrazolo[4,3-*d*]pyrimidine-7-amine (72.3 mg, 69%).

^{1}H-NMR (400 MHz, DMSO-*d*$_6$) δ 10.79 (s, 1H), 8.76 (t, *J* = 6.0 Hz, 1H), 8.30 (s, 1H), 7.67 (d, *J* = 7.9 Hz, 1H), 7.29 (d, *J* = 7.9 Hz, 1H), 7.17 (d, *J* = 2.1 Hz, 1H), 7.03 (t, *J* = 6.9 Hz, 1H), 6.94 (t, *J* = 6.9 Hz, 1H), 4.34 (t, *J* = 7.0 Hz, 2H), 3.69 (dd, *J* = 15.1, 6.0 Hz, 2H), 2.99 (t, *J* = 7.5 Hz, 2H), 1.87-1.80 (m, 2H), 1.21 (q, *J* = 7.5 Hz, 2H), 0.86 (t, *J* = 7.5 Hz, 3H)

Step 6. *N*-(2-(1H-indol-3-yl)ethyl)-5-chloro-2-butyl-2*H*-pyrazolo[4,3-*d*]pyrimidine-7-amine (72.3 mg, 0.2 mmol) and 3-cyano-phenylboronic acid (43.28 mg, 0.29 mmol) was stirred with 2M sodium bicarbonate solution (1 mL) and $Pd(PPh_3)_4$ (22.69 mg, 0.02 mmol) mixture in 10 mL of 1,4-dioxane at 90 °C for 3 hours. The reaction mixture was poured into brine and extracted with ethyl acetate. The combined organic layer was dried over anhydrous sodium sulfate, concentrated under vacuum. The concentrated product was purified by silica gel column chromatography (0-100% EA/n-Hexane) to yield 3-(7-((2-(1*H*-indol-3-yl)ethyl)amino)-2-butyl-2*H*-pyrazolo[4,3-*d*]pyrimidin-5-yl)benzonitrile (18 mg, 21%) as a solid.

HRMS (FAB) m/z calculated for $C_{26}H_{25}N_7$ $[M + H]^+$ 436.2250, found 436.2243; HPLC purity 98.2113%.

^{1}H-NMR (400 MHz, MeOH-*d*$_4$) δ 8.62 (s, 1H), 8.56 (dt, *J* = 7.9, 1.4 Hz, 1H), 8.15 (s, 1H), 7.77 (dt, *J* = 7.6, 1.3 Hz, 1H), 7.66-7.58 (m, 2H), 7.30 (d, *J* = 8.0 Hz, 1H), 7.11-6.99 (m, 3H), 4.42 (t, *J* = 7.1 Hz, 2H), 4.04 (t, *J* = 7.4 Hz, 2H), 3.21 (t, *J* = 7.2 Hz, 2H), 2.01-1.93 (m, 2H), 1.35 (td, *J* = 15.0, 7.4 Hz, 2H), 0.97 (t, *J* = 7.4 Hz, 3H)

^{13}C-NMR (101 MHz, DMSO-*d*$_6$) δ 176.3, 155.1, 153.7, 140.4, 138.8, 136.2, 132.6, 131.9, 130.7, 129.5, 127.2, 124.1, 122.6, 120.8, 118.9, 118.2, 118.2, 111.7, 111.3, 52.9, 40.6, 31.8, 24.8, 19.1, 13.3

3-(7-((2-(5-hydroxy-1H-indol-3-yl)ethyl)amino)-2-isopropyl-2H-pyrazolo[4,3-d]pyrimidin-5-yl)benzonitrile (7i)

Step 1. 5,7-dichloro-2-isopropyl-2*H*-pyrazolo[4,3-*d*]pyrimidine (50 mg, 0.22 mmol), was stirred with serotonin hydrochloride (69.03 mg, 0.33 mmol) in 25ml of IPA for 12 hours. Afterwards, the mixture was extracted with water and EA. The organic layer was dried with Na_2SO_4, and the dried extract was concentrated under reduced pressure. The resulting concentrate was purified on silica gel column chromatography (0-100% EA/n-Hexane) to yield 3-(2-((5-chloro-2-isopropyl-2*H*-pyrazolo[4,3-*d*]pyrimidin-7-yl)amino)ethyl)-1*H*-indol-5-ol (60.7 mg, 76%).

^{1}H-NMR (400 MHz, MeOH-*d*$_4$) δ 8.13 (d, *J* = 7.9 Hz, 2H), 8.05 (d, *J* = 10.7 Hz, 1H), 7.67 (d, *J* = 7.9 Hz, 1H), 7.44 (td, *J* = 7.9, 6.1 Hz, 1H), 7.33-7.31 (m, 1H), 7.18-7.12 (m, 2H), 7.10-7.05 (m, 1H), 7.01-6.97 (m, 1H), 4.59 (s, 1H), 4.08-4.01 (m, 2H), 3.21 (t, *J* = 7.5 Hz, 2H), 1.64-1.60 (m, 6H)

Step 2. 3-(2-((5-chloro-2-isopropyl-2*H*-pyrazolo[4,3-*d*]pyrimidin-7-yl)amino)ethyl)-1*H*-indol-5-ol (30 mg, 0.08 mmol) and 3-cyanophenylboronic acid (17.83 mg, 0.12 mmol) was stirred with 2M sodium bicarbonate solution (1 mL) and $Pd(PPh_3)_4$ (9.35 mg, 0.01 mmol) mixture in 10 mL of 1,4-dioxane at 90 °C for 3 hours. The reaction mixture was extracted with ethyl acetate and water. The combined organic layer was dried over anhydrous sodium sulfate, concentrated under vacuum. The concentrated product was purified by silica gel column chromatography (0-100% EA/n-Hexane) to yield 3-(7-((2-(5-hydroxy-1*H*-indol-3-yl)ethyl)amino)-2-isopropyl-2*H*-pyrazolo[4,3-*d*]pyrimidin-5-yl)benzonitrile (11.3 mg, 32%).

HRMS (FAB) m/z calculated for $C_{25}H_{23}N_7O$ $[M + H]^+$ 438.2042, found 438.2039; HPLC purity 98.7628%.

^{1}H-NMR (400 MHz, DMSO-*d*$_6$) δ 10.47 (s, 1H), 8.56-8.53 (m, 2H), 8.35-8.30 (m, 2H), 7.88 (d, *J* = 7.6 Hz, 1H), 7.78-7.73 (m, 1H), 7.59 (td, *J* = 7.6, 1.2 Hz, 1H), 7.12-7.10 (m, 2H), 6.89 (d, *J* = 2.1 Hz, 1H), 6.59 (dd, *J* = 8.5, 2.4 Hz, 1H), 4.82 (t, *J* = 6.7 Hz, 1H), 3.93 (q, *J* = 7.0 Hz, 2H), 3.01 (t, *J* = 7.6 Hz, 2H), 1.57 (d, *J* = 6.4 Hz, 6H)

^{13}C-NMR (101 MHz, MeOH-*d*$_4$) δ 171.7, 155.6, 154.0, 150.0, 135.5, 133.2, 132.8, 131.6, 131.3, 130.2, 128.7, 128.2, 123.4, 117.7, 117.5, 113.0, 111.2, 111.0, 110.8, 102.1, 57.2, 42.2, 25.2, 21.8

N-(2-(1H-indol-3-yl)ethyl)-5-(3-fluorophenyl)-2-isopropyl-2H-pyrazolo[4,3-d]pyrimidin-7-amine (7j)

N-(2-(1*H*-indol-3-yl)ethyl)-5-chloro-2-isopropyl-2*H*-pyrazolo[4,3-*d*]pyrimidine-7-amine (20 mg, 0.06 mmol) and 3-fluorophenylboronic acid (11.83 mg, 0.09 mmol) was stirred with 2M sodium bicarbonate solution (1 mL) and $Pd(PPh_3)_4$ (6.51 mg, 0.01 mmol) mixture in 10 mL of 1,4-dioxane at 90 °C for 3 hours. The reaction mixture was extracted with ethyl acetate and water. The combined organic layer was dried over anhydrous sodium sulfate, concentrated under vacuum. The concentrated product was purified by silica gel column chromatography (0-100% EA/n-Hexane) to yield *N*-(2-(1*H*-indol-3-yl)ethyl)-5-(3-fluorophenyl)-2-isopropyl-2*H*-pyrazolo[4,3-*d*]pyrimidin-7-amine (4.6 mg, 20%).

HRMS (FAB) m/z calculated for $C_{24}H_{23}FN_6$ $[M + H]^+$ 415.2046, found 415.2045; HPLC purity 98.1197%.

^{1}H-NMR (400 MHz, MeOH-*d*$_4$) δ 10.80 (s, 1H), 8.44 (s, 1H), 8.21 (d, *J* = 7.6 Hz, 1H), 8.09 (d, *J* = 11.0 Hz, 1H), 7.63 (t, *J* = 7.6 Hz, 1H), 7.48-7.43 (m, 1H), 7.31 (d, *J* = 7.9 Hz, 1H), 7.24-7.20 (m, 2H), 7.04 (t, *J* = 7.5 Hz, 1H), 6.95 (t, *J* = 7.5 Hz, 1H), 4.80-4.74 (m, 1H), 3.87 (t, *J* = 7.6 Hz, 2H), 3.09 (t, *J* = 7.6 Hz, 2H), 1.54-1.49 (m, 6H)

^{13}C-NMR (101 MHz, MeOH-*d*$_4$) δ 164.2, 161.8, 157.8, 154.4, 141.8, 138.5, 136.9, 130.3, 129.5, 129.5, 127.5, 123.6, 122.2, 121.0, 120.9, 118.3, 118.1, 115.9, 115.7, 114.5, 114.3, 112.1, 110.9, 56.1, 41.1, 25.1, 22.0

N-(2-(1H-indol-3-yl)ethyl)-5-(3,5-difluorophenyl)-2-isopropyl-2H-pyrazolo[4,3-d]pyrimidin-7-amine (7k)

N-(2-(1H-indol-3-yl)ethyl)-5-chloro-2-isopropyl-2H-pyrazolo[4,3-d]pyrimidine-7-amine (25 mg, 0.07 mmol) and 3,5-difluoro-phenylboronic acid (16.7 mg, 0.11 mmol) was stirred with 2M sodium bicarbonate solution (1 mL) and $Pd(PPh_3)_4$ (8.14 mg, 0.01 mmol) mixture in 10 mL of 1,4-dioxane at 90 °C for 3 hours. The reaction mixture was extracted with ethyl acetate and water. The combined organic layer was dried over anhydrous sodium sulfate, concentrated under vacuum. The concentrated product was purified by silica gel column chromatography (0-100% EA/n-Hexane) to yield N-(2-(1H-indol-3-yl)ethyl)-5-(3,5-difluorophenyl)-2-isopropyl-2H-pyrazolo[4,3-d]pyrimidin-7-amine (9.3 mg, 31%) as a solid. HRMS (FAB) m/z calculated for $C_{24}H_{22}F_2N_6$ $[M + H]^+$ 433.1952, found 433.1950; HPLC purity 97.6282%.

$^{1.}$ H-NMR (400 MHz, MeOH-d$_4$) δ 8.15 (s, 1H), 7.94-7.89 (m, 2H), 7.68-7.64 (m, 1H), 7.32 (d, J = 7.9 Hz, 1H), 7.11-7.06 (m, 2H), 7.02-6.96 (m, 2H), 4.78 (td, J = 13.3, 6.6 Hz, 1H), 4.00 (t, J = 7.5 Hz, 2H), 3.19 (t, J = 7.5 Hz, 2H), 1.60 (dd, J = 6.6, 2.6 Hz, 6H)

^{13}C-NMR (101 MHz, MeOH-*d*$_4$) δ 164.3, 161.8, 156.4, 154.3, 143.3, 138.4, 136.9, 130.3, 127.5, 122.2, 121.1, 121.0, 118.3, 118.1, 112.1, 110.9, 110.5, 110.2, 103.9, 56.0, 41.1, 25.1, 21.9

N-(2-(1H-indol-3-yl)ethyl)-2-isopropyl-5-(3-(trifluoromethoxy)phenyl)-2H-pyrazolo[4,3-d]pyrimidin-7-amine (7l)

N-(2-(1*H*-indol-3-yl)ethyl)-5-chloro-2-isopropyl-2*H*-pyrazolo[4,3-*d*]pyrimidine-7-amine (30 mg, 0.09 mmol) and 3-trifluoromethoxy-phenylboronic acid (26.12 mg, 0.13 mmol) was stirred with 2M sodium bicarbonate solution (1 mL) and $Pd(PPh_3)_4$ (9.77 mg, 0.01 mmol) mixture in 10 mL of 1,4-dioxane at 90 °C for 3 hours. The reaction mixture was extracted with ethyl acetate and water. The combined organic layer was dried over anhydrous sodium sulfate, concentrated under vacuum. The concentrated product was purified by silica gel column chromatography (0-100% EA/n-Hexane) to yield *N*-(2-(1*H*-indol-3-yl)ethyl)-2-isopropyl-5-(3-(trifluoromethoxy)phenyl)-2*H*-pyrazolo[4,3-d]pyrimidin-7-amine (15.3 mg, 38%) as a solid.

HRMS (FAB) m/z calculated for $C_{25}H_{23}F_3N_6O$ $[M + H]^+$ 481.1964, found 481.1968; HPLC purity 95.5667%.

^{1}H-NMR (400 MHz, MeOH-d_4) δ 8.28 (d, *J* = 8.0 Hz, 1H), 8.23 (s, 1H), 8.11 (s, 1H), 7.62 (d, *J* = 8.0 Hz, 1H), 7.48 (t, *J* = 8.0 Hz, 1H), 7.29 (d, *J* = 8.0 Hz, 2H), 7.08-7.03 (m, 2H), 6.98-6.94 (m, 1H), 4.79-4.72 (m, 1H), 4.00 (t, *J* = 7.2 Hz, 2H), 3.18 (t, *J* = 7.2 Hz, 2H), 1.57 (d, *J* = 6.9 Hz, 6H)

^{13}C-NMR (101 MHz, MeOH-d_4) δ 157.4, 154.4, 149.3, 141.7, 138.5, 136.9, 130.3, 129.4, 127.5, 126.5, 122.2, 122.0, 121.5, 121.0, 121.0, 120.2, 119.5, 118.3, 118.1, 112.1, 110.9, 56.0, 41.0, 25.1, 22.0

tert-butyl-3-(7-((2-(1H-indol-3-yl)ethyl)amino)-2-isopropyl-2H-pyrazolo[4,3-d]pyrimidin-5-yl)benzoate (7m)

N-(2-(1*H*-indol-3-yl)ethyl)-5-chloro-2-isopropyl-2*H*-pyrazolo[4,3-*d*]pyrimidine-7-amine (20 mg, 0.06 mmol) and (3-(tert-butoxycarbonyl)phenyl)boronic acid (18.77 mg, 0.09 mmol) was stirred with 2M sodium bicarbonate solution (1 mL) and $Pd(PPh_3)_4$ (6.51 mg, 0.01 mmol) mixture in 10 mL of 1,4-dioxane at 90 °C for 3 hours. The reaction mixture was extracted with ethyl acetate and water. The combined organic layer was dried over anhydrous sodium sulfate, concentrated under vacuum. The concentrated product was purified by silica gel column chromatography (0-100% EA/n-Hexane) to yield tert-butyl-3-(7-((2-(1*H*-indol-3-yl)ethyl)amino)-2-isopropyl-2*H*-pyrazolo[4,3-*d*]pyrimidin-5-yl)benzoate (17.5 mg, 63%) as a solid.

HRMS (FAB) m/z calculated for $C_{29}H_{32}N_6O_2$ $[M + H]^+$ 497.2665, found 497.2669; HPLC purity 98.7177%.

^{1}H-NMR (400 MHz, MeOH-d_4) δ 8.97 (s, 1H), 8.49 (d, *J* = 7.6 Hz, 1H), 8.16 (s, 1H), 8.02 (d, *J* = 8.0 Hz, 1H), 7.65 (d, *J* = 7.6 Hz, 1H), 7.53 (t, *J* = 7.6 Hz, 1H), 7.31 (d, *J* = 8.0 Hz, 1H), 7.15 (s, 1H), 7.06 (t, *J* = 7.4 Hz, 1H), 6.95 (t, *J* = 7.2 Hz, 1H), 4.06 (t, *J* = 7.4 Hz, 2H), 3.24 (t, *J* = 7.4 Hz, 2H), 1.62 (d, *J* = 6.5 Hz, 6H), 1.57 (s, 9H)

^{13}C-NMR (101 MHz, MeOH-d_4) δ 166.0, 158.3, 154.5, 139.7, 139.6, 138.5, 136.9, 132.0, 131.9, 130.3, 129.9, 128.9, 128.0, 127.6, 122.2, 120.9, 118.3, 118.2, 112.2, 110.9, 81.0, 56.1, 41.1, 27.1, 25.1, 22.0

HPLC purity analysis

N-(2-(1*H*-indol-3-yl)ethyl)-2-isopropyl-5-phenyl-2*H*-pyrazolo[4,3-*d*]pyrimidin-7-amine (7a)

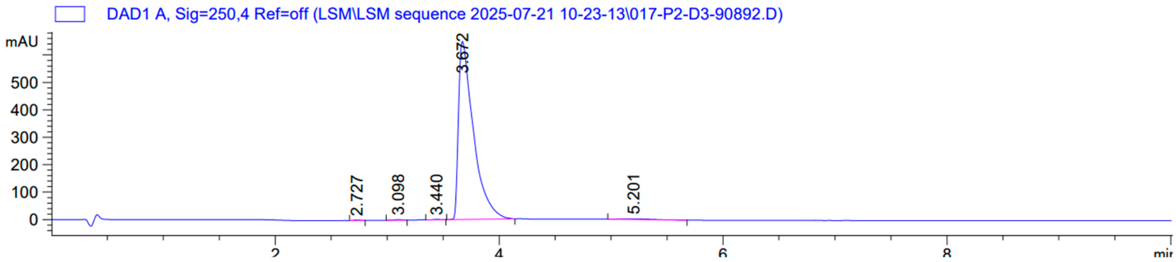

Signal 1: DAD1 A, Sig=250,4 Ref=off

Peak #	RetTime [min]	Type	Width [min]	Area [mAU*s]	Height [mAU]	Area %
1	2.727	BB	0.0558	5.10606	1.35965	0.0836
2	3.098	BB	0.0548	8.19758	2.23556	0.1343
3	3.440	BB	0.0570	6.91085	1.79259	0.1132
4	3.672	BB	0.1325	6043.66797	649.76080	99.0033
5	5.201	BB	0.2328	40.62634	2.08985	0.6655
Totals :				6104.50879	657.23846	

4-(7-((2-(1*H*-indol-3-yl)ethyl)amino)-2-isopropyl-2*H*-pyrazolo[4,3-*d*]pyrimidin-5-yl)benzonitrile

(7b)

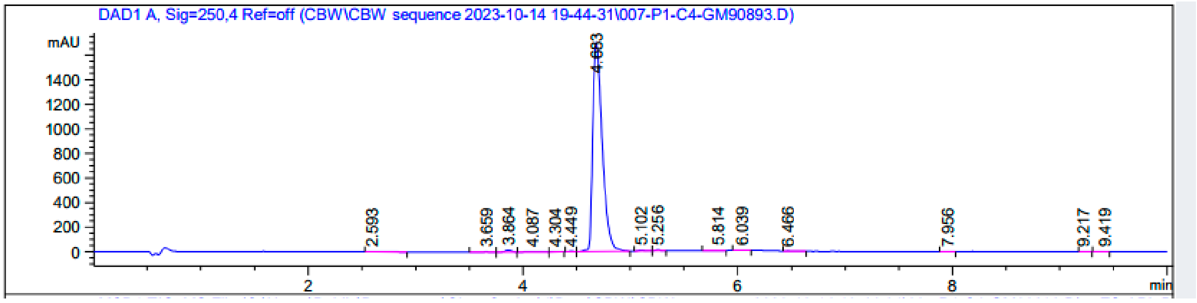

Signal 1: DAD1 A, Sig=250,4 Ref=off

Peak #	RetTime [min]	Type	Width [min]	Area [mAU*s]	Height [mAU]	Area %
1	2.593	BB	0.1580	20.89239	1.62537	0.1956
2	3.659	BB	0.0923	19.03686	2.91097	0.1783
3	3.864	BB	0.0596	64.21117	16.41806	0.6013
4	4.087	BB	0.1086	25.82426	3.40211	0.2418
5	4.304	BB	0.0592	10.42774	2.68993	0.0976
6	4.449	BB	0.0436	8.93168	3.30150	0.0836
7	4.683	BB	0.0958	1.04339e4	1695.44104	97.7059
8	5.102	BB	0.0616	22.89691	5.61005	0.2144
9	5.256	BB	0.0503	27.24086	8.75784	0.2551
10	5.814	BB	0.1012	8.35074	1.02308	0.0782
11	6.039	BB	0.0670	9.79031	2.15420	0.0917
12	6.466	BB	0.0643	8.34631	1.72948	0.0782
13	7.956	BB	0.0627	5.54140	1.32633	0.0519
14	9.217	BB	0.0670	5.98160	1.22511	0.0560
15	9.419	BB	0.0850	7.51152	1.20007	0.0703
Totals :				1.06788e4	1748.81513	

3-(7-((2-(1*H*-indol-3-yl)ethyl)amino)-2-isopropyl-2*H*-pyrazolo[4,3-*d*]pyrimidin-5-yl)benzonitrile (7c)

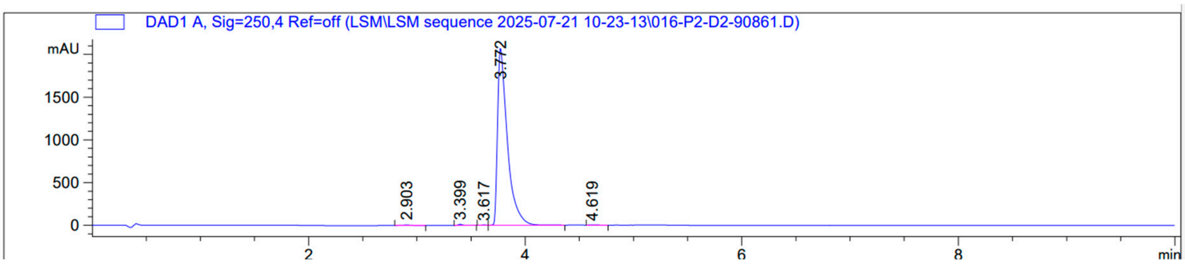

Signal 1: DAD1 A, Sig=250,4 Ref=off

Peak #	RetTime [min]	Type	Width [min]	Area [mAU*s]	Height [mAU]	Area %
1	2.903	BB	0.0676	35.90430	7.26652	0.2734
2	3.399	BB	0.0506	41.03966	12.38802	0.3125
3	3.617	BB	0.0495	7.51639	2.47311	0.0572
4	3.772	BB	0.0933	1.30421e4	2076.71655	99.3088
5	4.619	BB	0.0620	6.31437	1.47253	0.0481
Totals :				1.31329e4	2100.31674	

1-(3-(7-((2-(1*H*-indol-3-yl)ethyl)amino)-2-isopropyl-2*H*-pyrazolo[4,3-*d*]pyrimidin-5-yl)phenyl)ethan-1-one (7d)

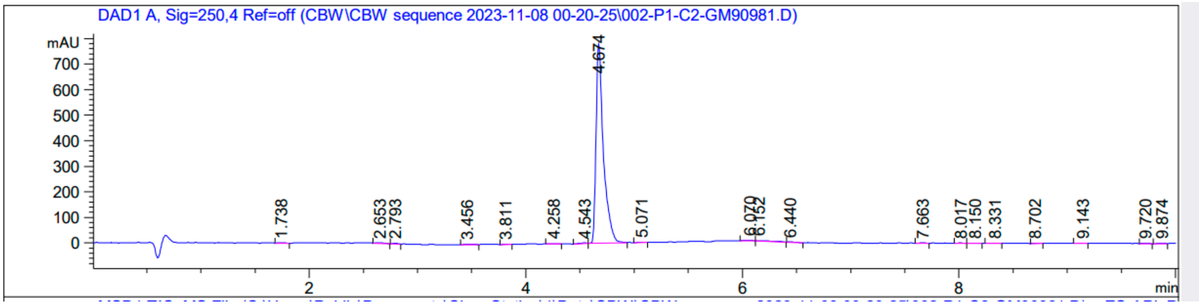


```
Signal 1: DAD1 A, Sig=250,4 Ref=off
```

Peak #	RetTime [min]	Type	Width [min]	Area [mAU*s]	Height [mAU]	Area %
1	1.738	BB	0.0512	7.47479	2.01651	0.1937
2	2.653	BB	0.0618	13.28044	2.87753	0.3441
3	2.793	BB	0.0608	6.48145	1.85186	0.1680
4	3.456	BB	0.0776	9.56669	1.70255	0.2479
5	3.811	BB	0.0437	5.49888	1.70227	0.1425
6	4.258	BB	0.0878	9.73291	1.83757	0.2522
7	4.543	BB	0.0620	10.60098	2.37687	0.2747
8	4.674	BB	0.0693	3703.38818	780.85376	95.9668
9	5.071	BB	0.0593	9.36751	2.30895	0.2427
10	6.070	BB	0.0752	10.46087	1.93191	0.2711
11	6.152	BB	0.1798	16.06676	1.07856	0.4163
12	6.440	BB	0.0850	5.84223	1.01756	0.1514
13	7.663	BB	0.0572	7.20124	1.64164	0.1866
14	8.017	BB	0.0603	5.61214	1.41444	0.1454
15	8.150	BB	0.0602	5.77247	1.29023	0.1496
16	8.331	BB	0.0617	5.61119	1.19100	0.1454
17	8.702	BB	0.0554	6.18029	1.58869	0.1602
18	9.143	BB	0.0621	5.42809	1.31792	0.1407
19	9.720	BB	0.0632	7.84073	1.93855	0.2032
20	9.874	BB	0.0521	7.62177	2.01635	0.1975
Totals :				3859.02961	813.95470	

N-(2-(1*H*-indol-3-yl)ethyl)-2-isopropyl-5-(3-(trifluoromethyl)phenyl)-2*H*-pyrazolo[4,3-*d*]pyrimidin-7-amine (7e)

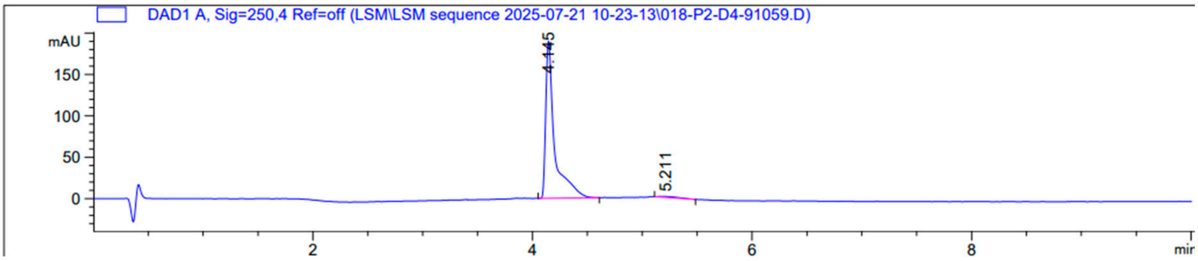

Signal 1: DAD1 A, Sig=250,4 Ref=off

Peak #	RetTime [min]	Type	Width [min]	Area [mAU*s]	Height [mAU]	Area %
1	4.145	BB	0.0795	1067.33521	189.97682	98.6371
2	5.211	BB	0.1477	14.74743	1.23188	1.3629
Totals :				1082.08263	191.20870	

N-(2-(1*H*-indol-3-yl)ethyl)-5-(3-aminophenyl)-2-isopropyl-2*H*-pyrazolo[4,3-*d*]pyrimidin-7-amine (7f)

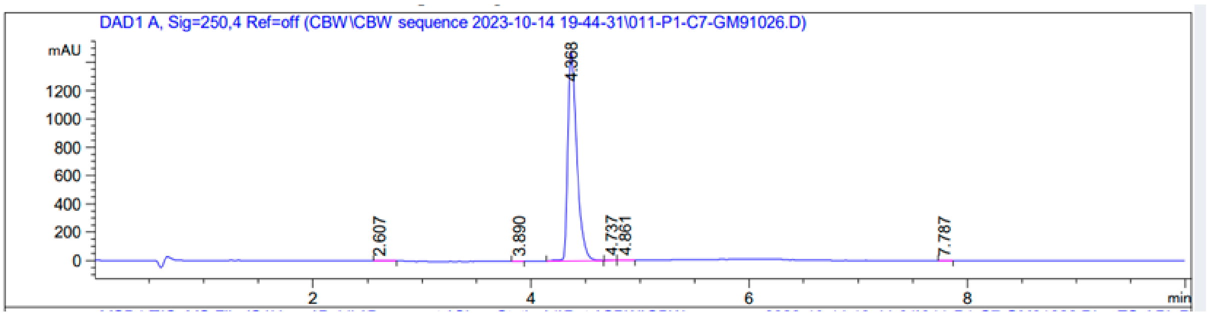

Signal 1: DAD1 A, Sig=250,4 Ref=off

Peak #	RetTime [min]	Type	Width [min]	Area [mAU*s]	Height [mAU]	Area %
1	2.607	BB	0.1137	10.89787	1.25002	0.1294
2	3.890	BB	0.0595	5.80273	1.42426	0.0689
3	4.368	BB	0.0842	8386.17578	1477.33069	99.5489
4	4.737	BB	0.0558	9.19523	2.69589	0.1092
5	4.861	BB	0.0750	6.50082	1.13279	0.0772
6	7.787	BB	0.0636	5.60901	1.17740	0.0666
Totals :				8424.18144	1485.01105	

3-(7-((2-(1*H*-indol-3-yl)ethyl)amino)-2-ethyl-2*H*-pyrazolo[4,3-*d*]pyrimidin-5-yl)benzonitrile (7g)

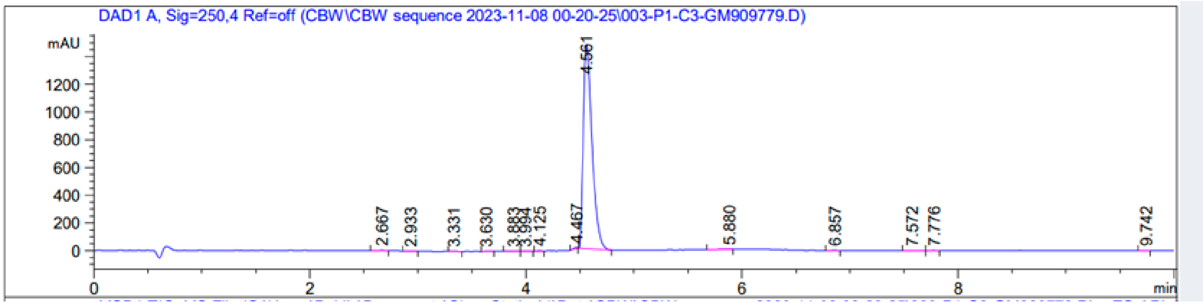

Signal 1: DAD1 A, Sig=250,4 Ref=off

Peak #	RetTime [min]	Type	Width [min]	Area [mAU*s]	Height [mAU]	Area %
1	2.667	BB	0.0701	10.60410	2.20502	0.1269
2	2.933	BB	0.0677	8.69579	1.82006	0.1041
3	3.331	BB	0.0562	5.70334	1.50486	0.0682
4	3.630	BB	0.0501	5.36228	1.56078	0.0642
5	3.883	BB	0.0607	11.40442	2.43178	0.1365
6	3.994	BB	0.0560	8.11126	2.14855	0.0971
7	4.125	BB	0.0535	7.04476	2.31516	0.0843
8	4.467	BB	0.0443	17.71708	6.40136	0.2120
9	4.561	BB	0.0852	8242.96387	1474.44690	98.6363
10	5.880	BB	0.0861	7.32628	1.06719	0.0877
11	6.857	BB	0.0586	10.34683	2.70763	0.1238
12	7.572	BB	0.0828	7.70731	1.27000	0.0922
13	7.776	BB	0.0661	7.88136	1.91216	0.0943
14	9.742	BB	0.0522	6.06176	1.67403	0.0725
Totals :				8356.93043	1503.46548	

3-(7-((2-(1H-indol-3-yl)ethyl)amino)-2-butyl-2H-pyrazolo[4,3-d]pyrimidin-5-yl)benzonitrile (7h)

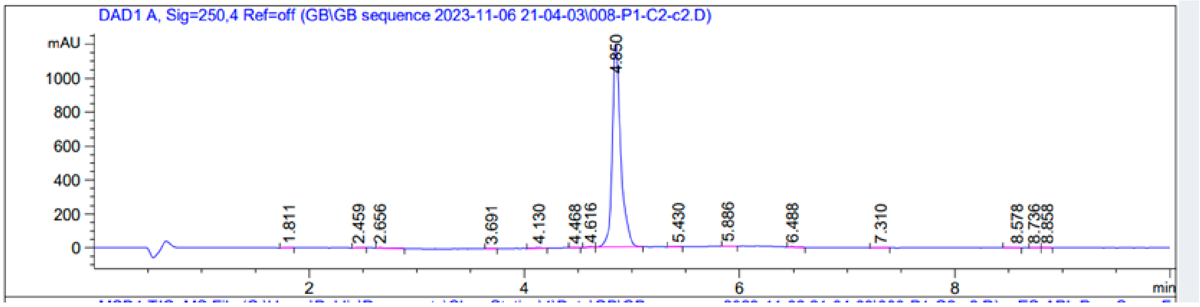

Signal 1: DAD1 A, Sig=250,4 Ref=off

Peak #	RetTime [min]	Type	Width [min]	Area [mAU*s]	Height [mAU]	Area %
1	1.811	BB	0.0495	5.14464	1.60250	0.0770
2	2.459	BB	0.0582	6.30225	1.52639	0.0943
3	2.656	BB	0.1308	12.29340	1.16801	0.1839
4	3.691	BB	0.0550	5.47157	1.48294	0.0818
5	4.130	BB	0.0718	19.55833	3.94794	0.2926
6	4.468	BB	0.0454	5.66612	1.67781	0.0848
7	4.616	BB	0.0498	14.93320	4.87315	0.2234
8	4.850	BB	0.0797	6565.42627	1203.05981	98.2113
9	5.430	BB	0.0652	6.02629	1.32199	0.0901
10	5.886	BB	0.0674	6.57349	1.24909	0.0983
11	6.488	BB	0.0632	7.85882	1.79055	0.1176
12	7.310	BB	0.0750	11.32364	2.03117	0.1694
13	8.578	BB	0.0618	6.62748	1.38570	0.0991
14	8.736	BB	0.0487	5.31001	1.68885	0.0794
15	8.858	BB	0.0493	6.48294	2.27251	0.0970
Totals :				6684.99845	1231.07842	

3-(7-((2-(5-hydroxy-1*H*-indol-3-yl)ethyl)amino)-2-isopropyl-2*H*-pyrazolo[4,3-*d*]pyrimidin-5-yl)benzonitrile (7i)

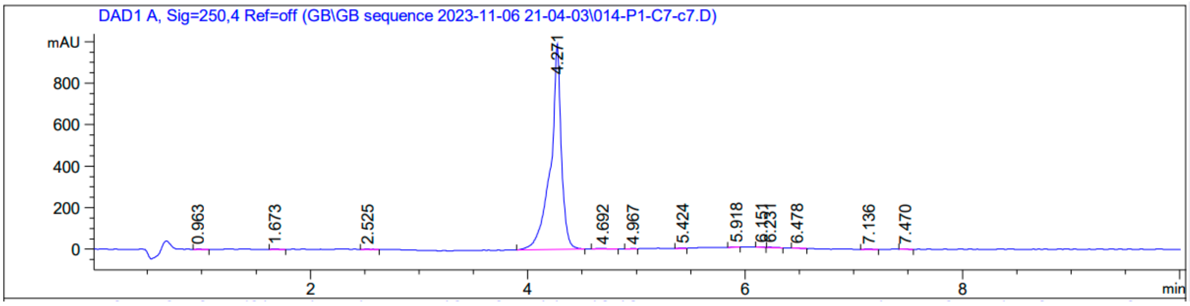

Signal 1: DAD1 A, Sig=250,4 Ref=off

Peak #	RetTime [min]	Type	Width [min]	Area [mAU*s]	Height [mAU]	Area %
1	0.963	BB	0.0632	7.14464	1.45734	0.1031
2	1.673	BB	0.0592	6.82122	1.55297	0.0985
3	2.525	BB	0.0794	7.36985	1.17137	0.1064
4	4.271	BB	0.0946	6842.67773	990.41650	98.7628
5	4.692	BB	0.0954	11.98959	1.56472	0.1731
6	4.967	BB	0.0518	5.25519	1.62268	0.0758
7	5.424	BB	0.0471	6.06414	2.01369	0.0875
8	5.918	BB	0.0621	5.19596	1.16462	0.0750
9	6.151	BB	0.0466	5.94943	2.00888	0.0859
10	6.231	BB	0.0681	8.69867	1.80740	0.1256
11	6.478	BB	0.0565	7.64097	1.83815	0.1103
12	7.136	BB	0.0653	6.92584	1.51588	0.1000
13	7.470	BB	0.0638	6.66330	1.34459	0.0962

Totals : 6928.39654 1009.47879

N-(2-(1H-indol-3-yl)ethyl)-5-(3-fluorophenyl)-2-isopropyl-2H-pyrazolo[4,3-d]pyrimidin-7-amine

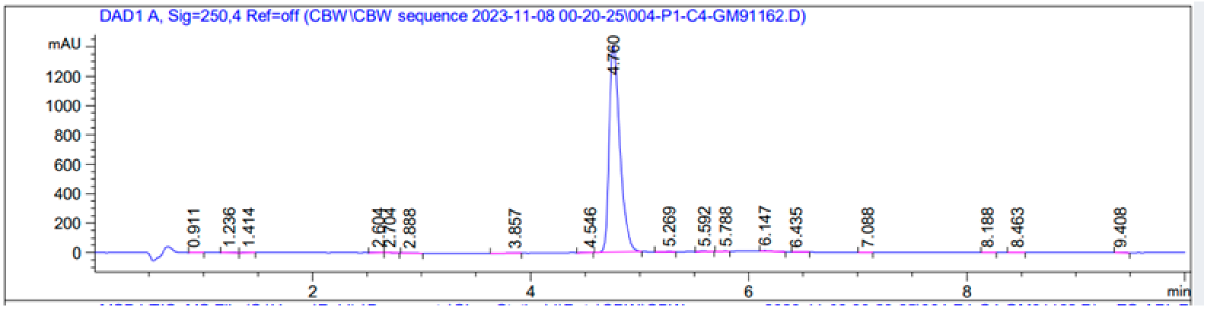

(7j)

Signal 1: DAD1 A, Sig=250,4 Ref=off

Peak #	RetTime [min]	Type	Width [min]	Area [mAU*s]	Height [mAU]	Area %
1	0.911	BB	0.0600	5.96561	1.51177	0.0609
2	1.236	BB	0.0759	13.88612	2.61844	0.1417
3	1.414	BB	0.0663	9.19150	2.04888	0.0938
4	2.604	BB	0.0575	8.28727	2.12292	0.0845
5	2.704	BB	0.0662	7.45600	1.49339	0.0761
6	2.888	BB	0.0858	14.88037	2.41921	0.1518
7	3.857	BB	0.0895	14.11416	2.12382	0.1440
8	4.546	BB	0.0603	5.18886	1.11492	0.0529
9	4.760	BB	0.1036	9618.43848	1409.32532	98.1197
10	5.269	BB	0.0693	13.66497	2.99357	0.1394
11	5.592	BB	0.0924	22.80002	3.77839	0.2326
12	5.788	BB	0.0502	7.96097	2.20012	0.0812
13	6.147	BB	0.0773	16.63214	2.88185	0.1697
14	6.435	BB	0.0876	8.93283	1.27765	0.0911
15	7.088	BB	0.0705	9.31208	1.99164	0.0950
16	8.188	BB	0.0598	8.64596	2.11184	0.0882
17	8.463	BB	0.0644	6.54353	1.35366	0.0668
18	9.408	BB	0.0602	10.86082	2.62964	0.1108
Totals :				9802.76169	1445.99705	

N-(2-(1*H*-indol-3-yl)ethyl)-5-(3,5-difluorophenyl)-2-isopropyl-2*H*-pyrazolo[4,3-*d*]pyrimidin-7-amine (7k)

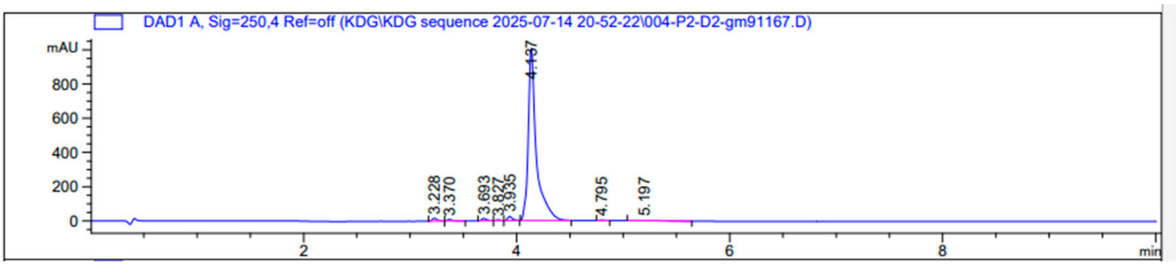


```
Signal 1: DAD1 A, Sig=250,4 Ref=off
```

Peak #	RetTime [min]	Type	Width [min]	Area [mAU*s]	Height [mAU]	Area %
1	3.228	BB	0.0511	51.53830	16.19594	0.9236
2	3.370	BB	0.0501	27.91042	8.55334	0.5002
3	3.693	BB	0.0491	43.23708	13.59500	0.7749
4	3.827	BB	0.0425	12.41185	4.74823	0.2224
5	3.935	BB	0.0510	73.51533	23.18324	1.3175
6	4.137	BB	0.0736	5328.65674	1008.56653	95.4958
7	4.795	BB	0.0474	5.14670	1.69437	0.0922
8	5.197	BB	0.2507	37.57685	1.83640	0.6734
Totals :				5579.99327	1078.37305	

N-(2-(1*H*-indol-3-yl)ethyl)-2-isopropyl-5-(3-(trifluoromethoxy)phenyl)-2*H*-pyrazolo[4,3-d]pyrimidin-7-amine (7l)

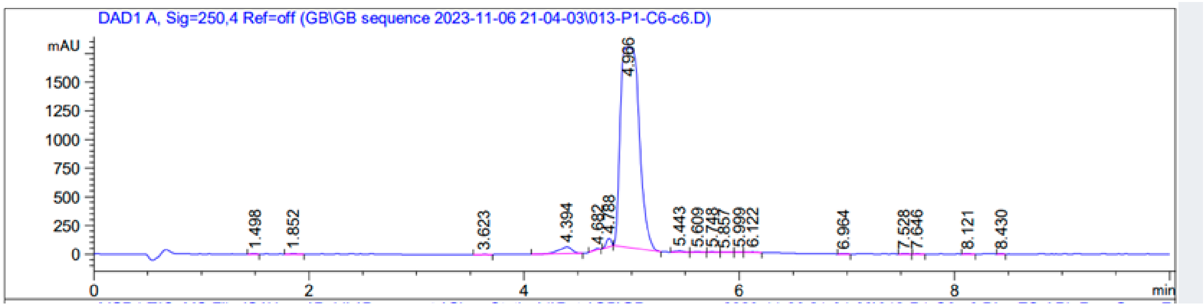

Signal 1: DAD1 A, Sig=250,4 Ref=off

Peak #	RetTime [min]	Type	Width [min]	Area [mAU*s]	Height [mAU]	Area %
1	1.498	BB	0.0473	5.60513	1.75207	0.0253
2	1.852	BB	0.0662	6.78757	1.46067	0.0306
3	3.623	BB	0.0808	9.79646	1.66033	0.0442
4	4.394	BB	0.1261	524.27533	59.91590	2.3648
5	4.682	BB	0.0503	54.98228	16.74775	0.2480
6	4.788	BB	0.0485	217.90814	73.58375	0.9829
7	4.966	BB	0.1718	2.12095e4	1745.33911	95.6667
8	5.443	BB	0.0811	47.48154	9.69433	0.2142
9	5.609	BB	0.0587	17.59221	4.59206	0.0794
10	5.748	BB	0.0518	15.15758	4.44702	0.0684
11	5.857	BB	0.0470	7.15885	2.25644	0.0323
12	5.999	BB	0.0385	7.29179	2.98116	0.0329
13	6.122	BB	0.0631	17.90869	4.09141	0.0808
14	6.964	BB	0.0550	6.39250	1.65648	0.0288
15	7.528	BB	0.0598	6.74577	1.51969	0.0304
16	7.646	BB	0.0515	5.40417	1.59422	0.0244
17	8.121	BB	0.0696	5.04253	1.23465	0.0227
18	8.430	BB	0.0375	5.16084	2.19139	0.0233
Totals :				2.21702e4	1936.71843	

tert-butyl-3-(7-((2-(1*H*-indol-3-yl)ethyl)amino)-2-isopropyl-2*H*-pyrazolo[4,3-*d*]pyrimidin-5-yl)benzoate (7m)

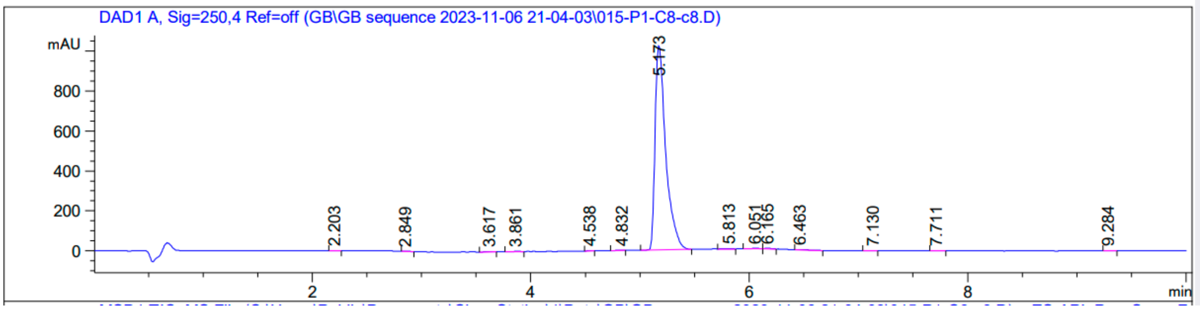

Signal 1: DAD1 A, Sig=250,4 Ref=off

Peak #	RetTime [min]	Type	Width [min]	Area [mAU*s]	Height [mAU]	Area %
1	2.203	BB	0.0489	6.02283	2.01356	0.0856
2	2.849	BB	0.0588	5.00637	1.24893	0.0711
3	3.617	BB	0.0621	5.02070	1.17019	0.0713
4	3.861	BB	0.0706	6.50411	1.25122	0.0924
5	4.538	BB	0.0437	5.50430	2.02772	0.0782
6	4.832	BB	0.0614	7.07501	1.60473	0.1005
7	5.173	BB	0.1033	6947.09961	1022.52039	98.7177
8	5.813	BB	0.0642	5.62865	1.26698	0.0800
9	6.051	BB	0.0736	8.97047	1.59578	0.1275

Peak #	RetTime [min]	Type	Width [min]	Area [mAU*s]	Height [mAU]	Area %
10	6.165	BB	0.0565	9.43271	2.36776	0.1340
11	6.463	BB	0.0917	12.65968	1.90125	0.1799
12	7.130	BB	0.0643	5.58823	1.34978	0.0794
13	7.711	BB	0.0580	6.01350	1.52464	0.0855
14	9.284	BB	0.0628	6.81054	1.56490	0.0968
Totals :				7037.33671	1043.40783	

^{1}H NMR and ^{13}C NMR spectra

***N*-(2-(1*H*-indol-3-yl)ethyl)-2-isopropyl-5-phenyl-2*H*-pyrazolo[4,3-*d*]pyrimidin-7-amine (7a)**

^{1}H NMR, ^{13}C NMR

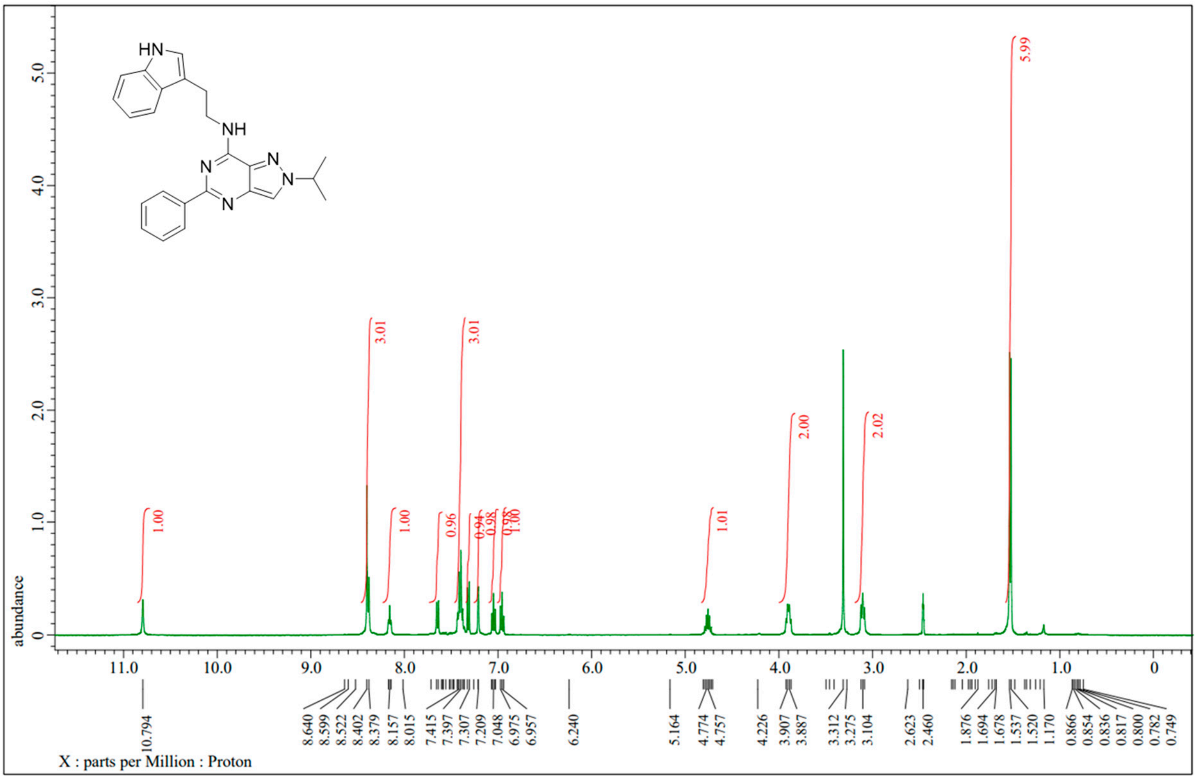

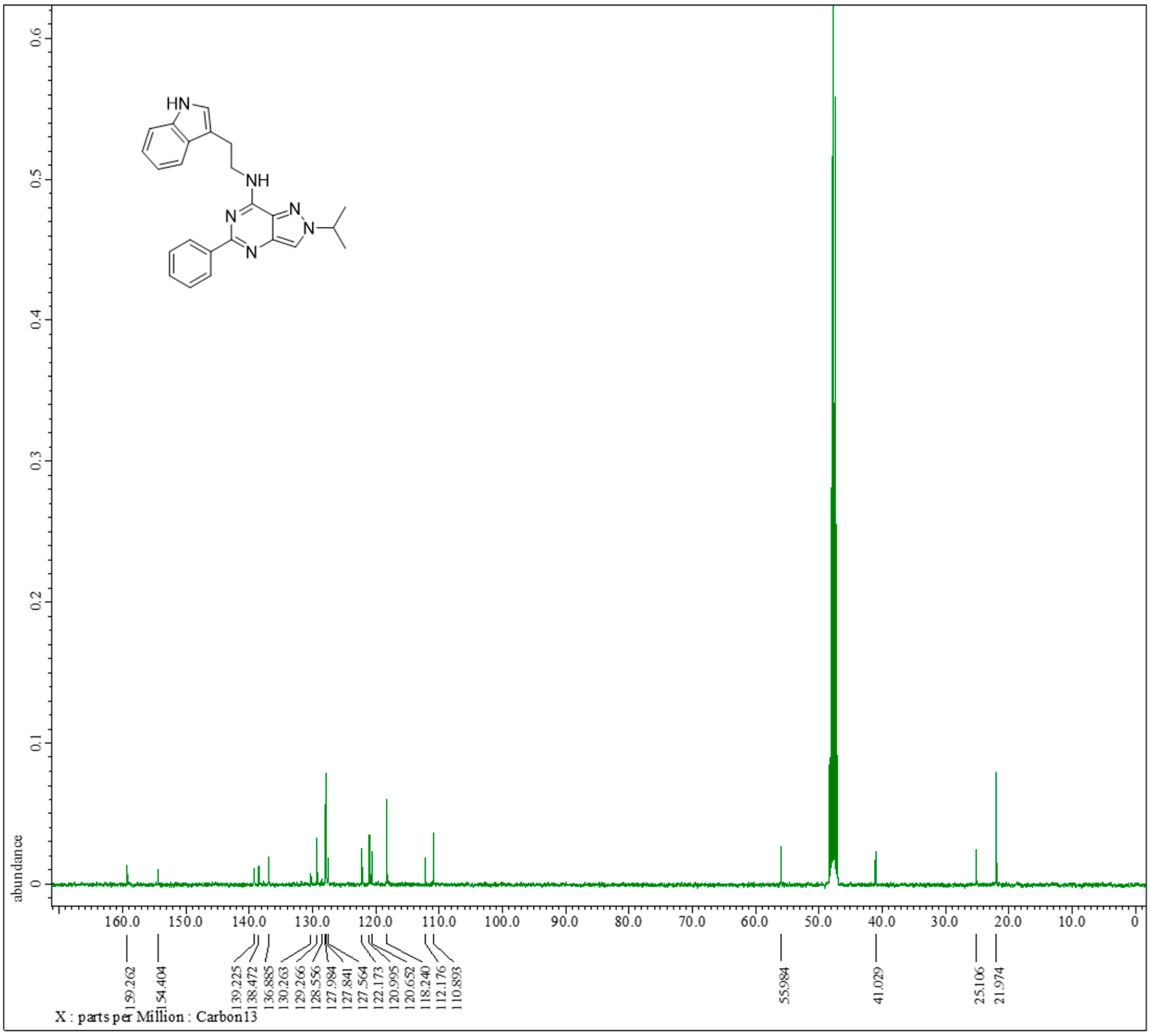

4-(7-((2-(1*H*-indol-3-yl)ethyl)amino)-2-isopropyl-2*H*-pyrazolo[4,3-*d*]pyrimidin-5-yl)benzonitrile (7b)

^{1}H NMR, ^{13}C NMR

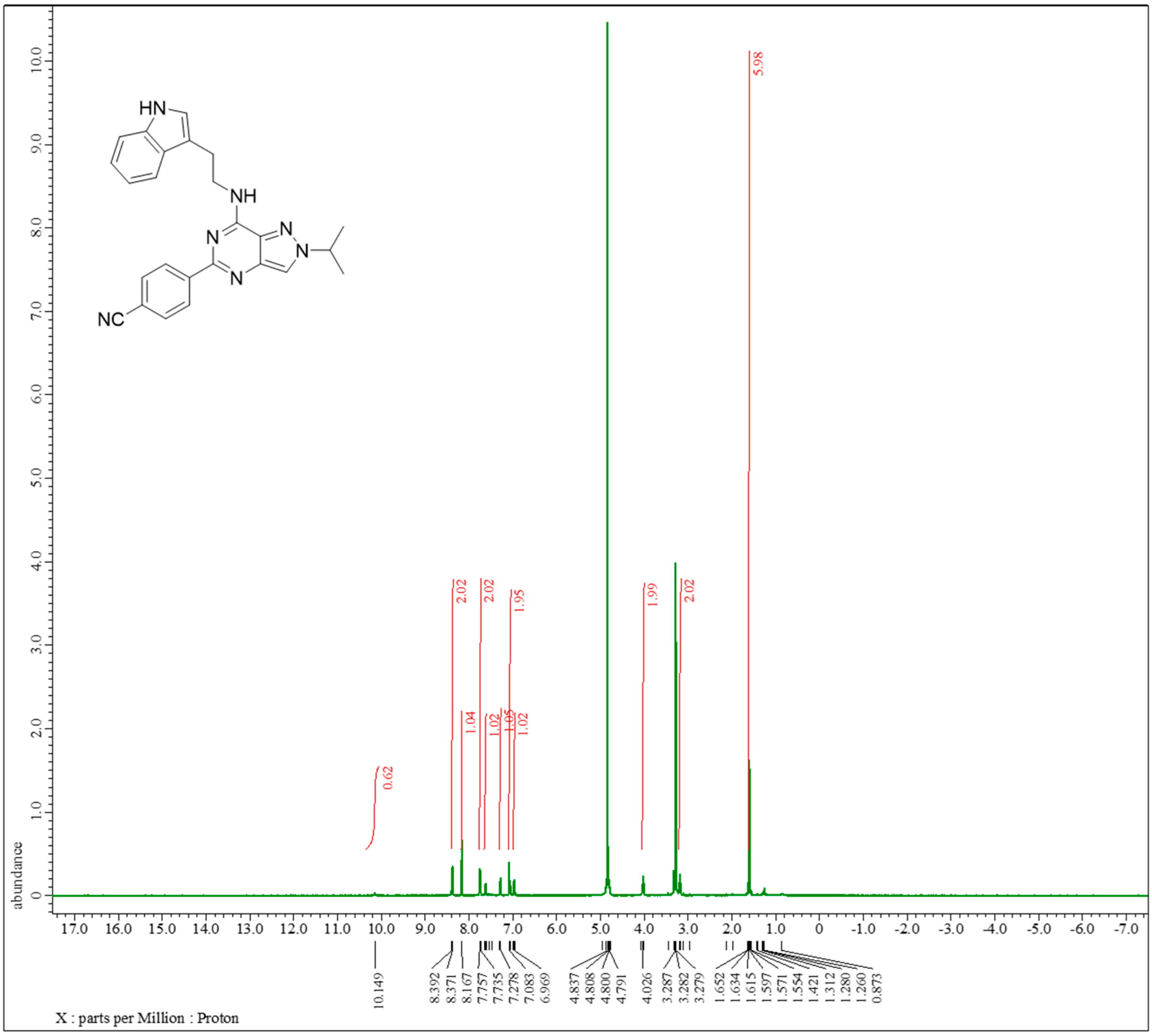

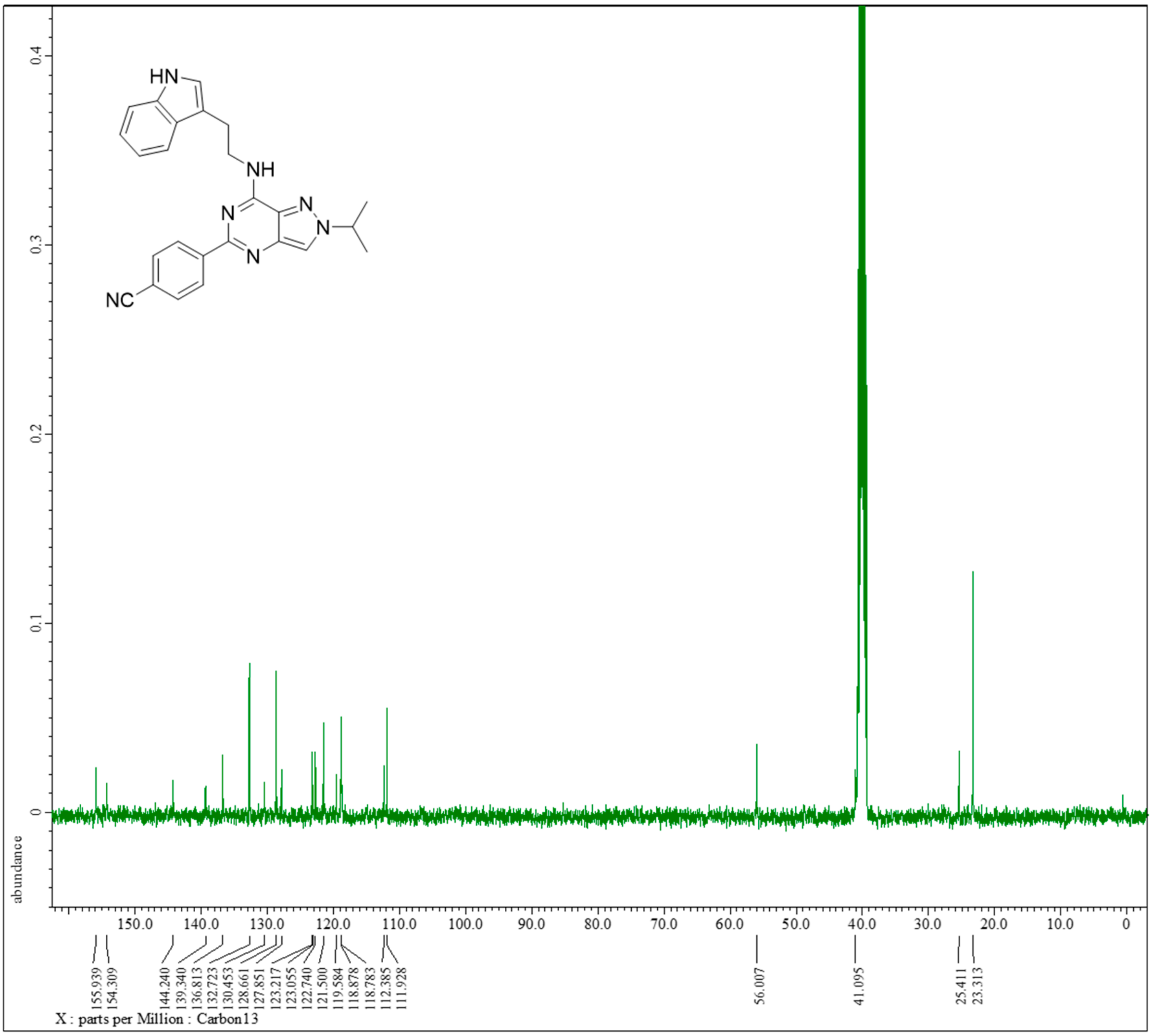
HN
NH
N
N
N
N
NC
abundance
0.4
0.3
0.2
0.1
0
150.0
140.0
130.0
120.0
110.0
100.0
90.0
80.0
70.0
60.0
50.0
40.0
30.0
20.0
10.0
0
155.939
154.309
144.240
139.340
136.813
132.723
130.453
128.661
127.851
123.217
123.055
122.740
121.500
119.584
118.878
118.783
112.385
111.928
56.007
41.095
25.411
23.313
X : parts per Million : Carbon13

3-(7-((2-(1*H*-indol-3-yl)ethyl)amino)-2-isopropyl-2*H*-pyrazolo[4,3-*d*]pyrimidin-5-yl)benzonitrile (7c)

^{1}H NMR, ^{13}C NMR

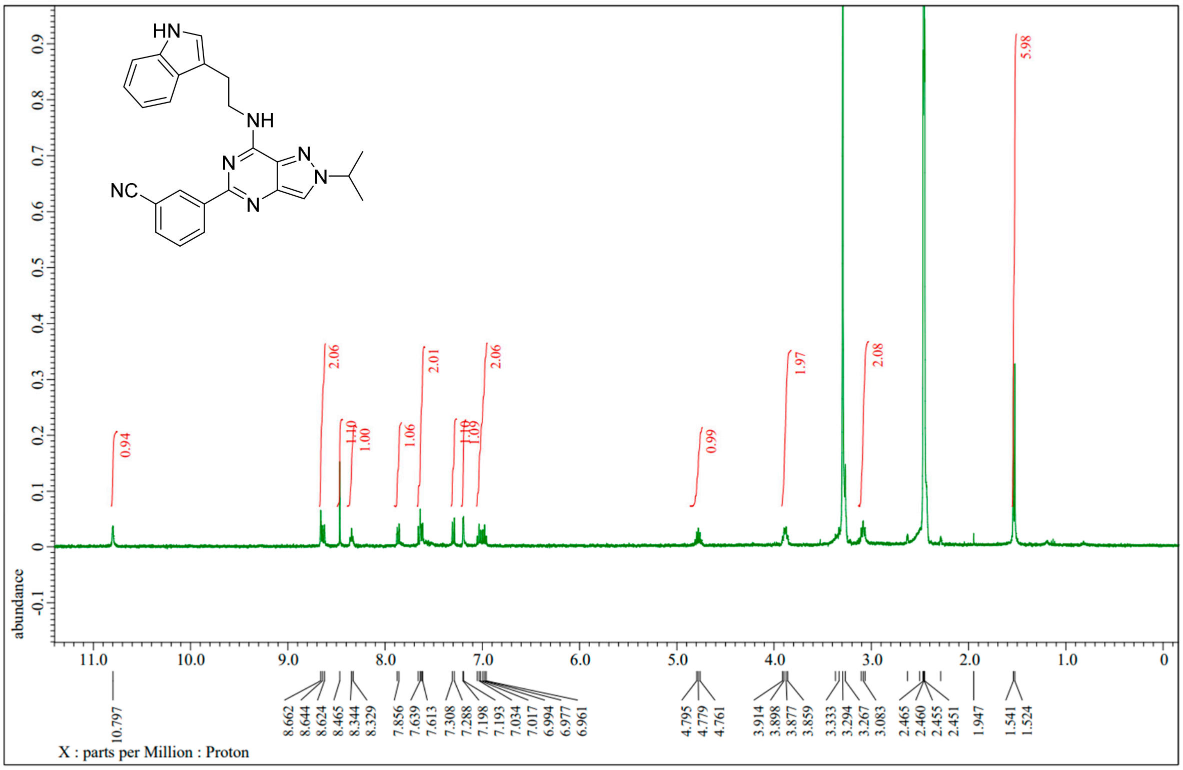

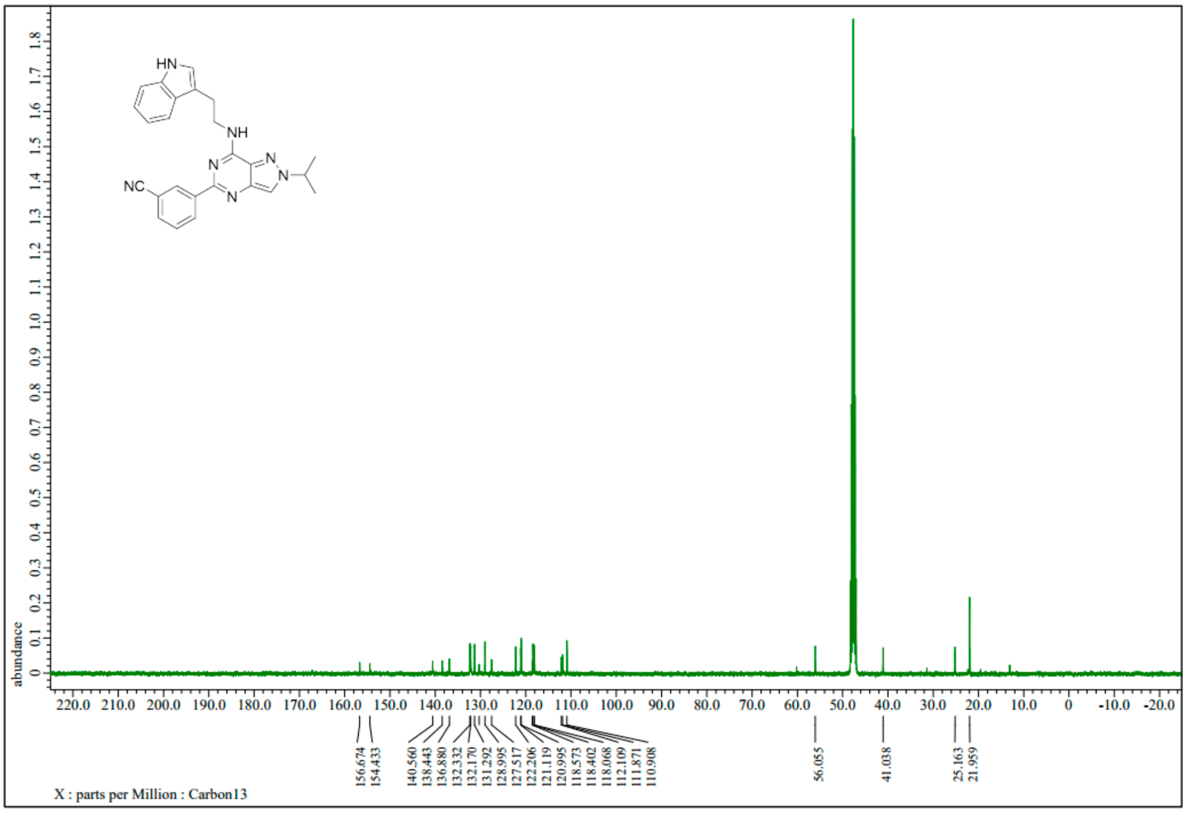

1-(3-(7-((2-(1*H*-indol-3-yl)ethyl)amino)-2-isopropyl-2*H*-pyrazolo[4,3-*d*]pyrimidin-5-yl)phenyl)ethan-1-one (7d)

^{1}H NMR, ^{13}C NMR

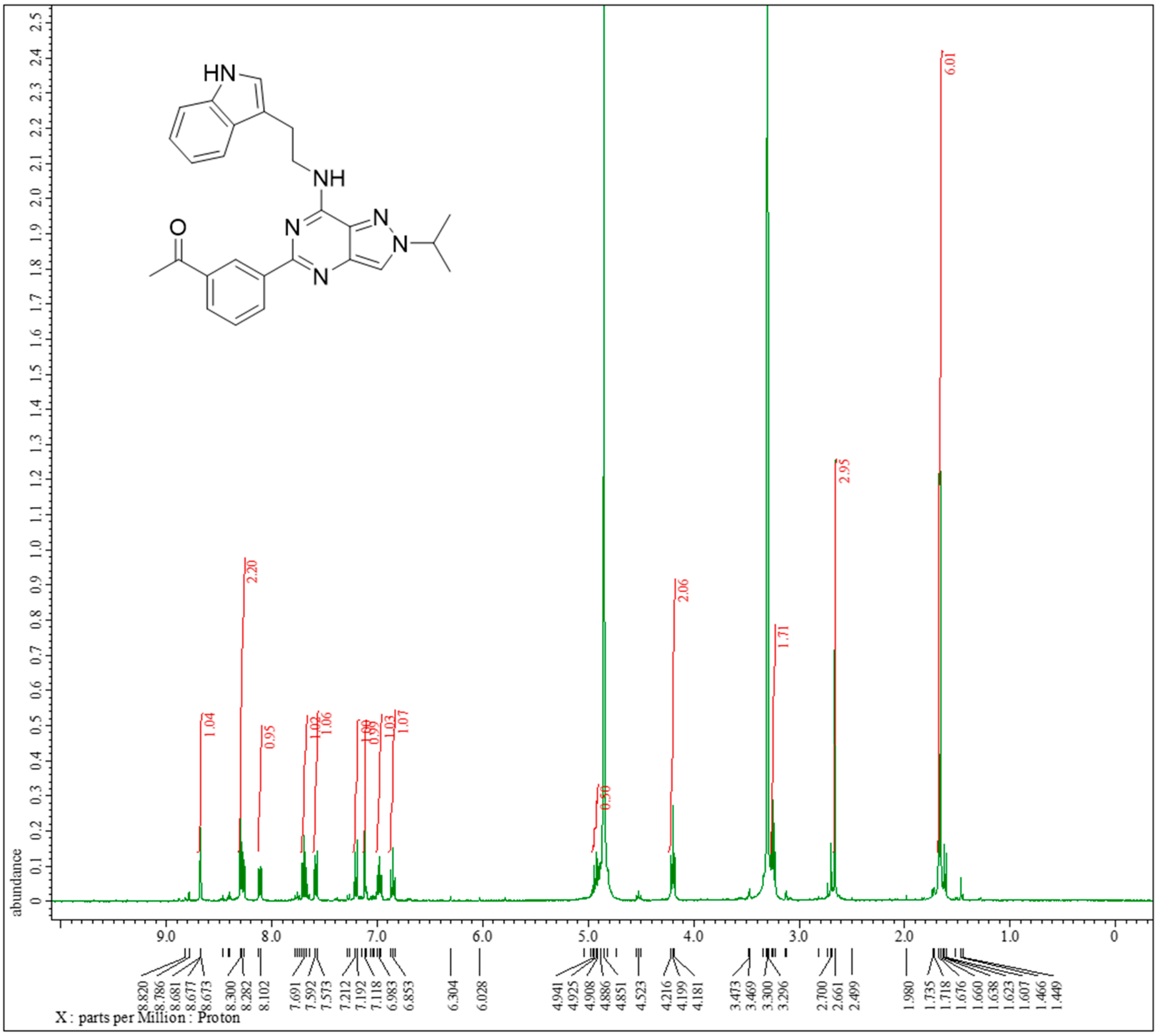

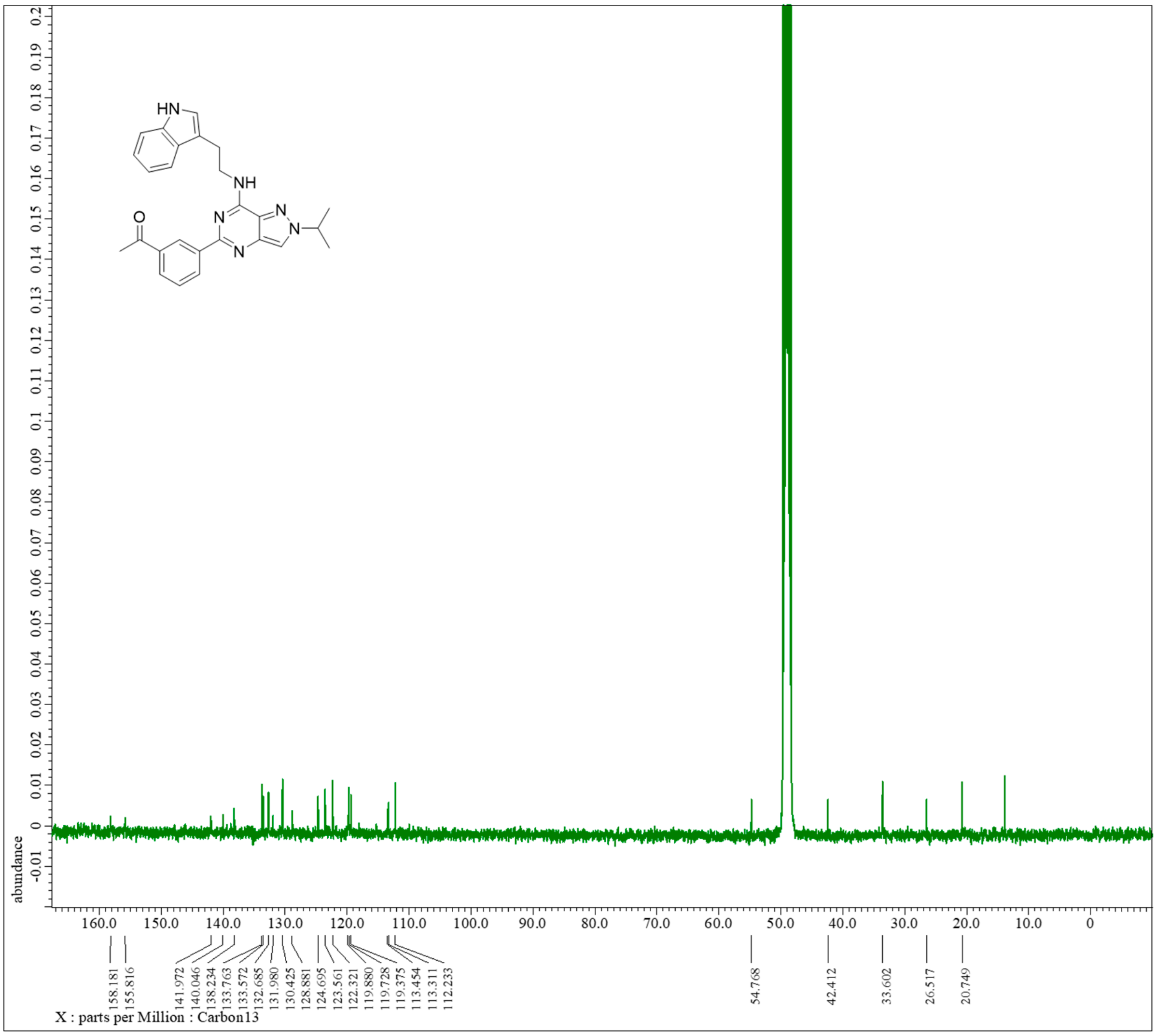

HN
NH
O
N
N
N
N
abundance
X : parts per Million : Carbon13
158.181
155.816
141.972
140.046
138.234
133.763
133.572
132.685
131.980
130.425
128.881
124.695
123.561
122.321
119.880
119.728
119.375
113.454
113.311
112.233
54.768
42.412
33.602
26.517
20.749

N-(2-(1*H*-indol-3-yl)ethyl)-2-isopropyl-5-(3-(trifluoromethyl)phenyl)-2*H*-pyrazolo[4,3-*d*]pyrimidin-7-amine (7e)

^{1}H NMR, ^{13}C NMR

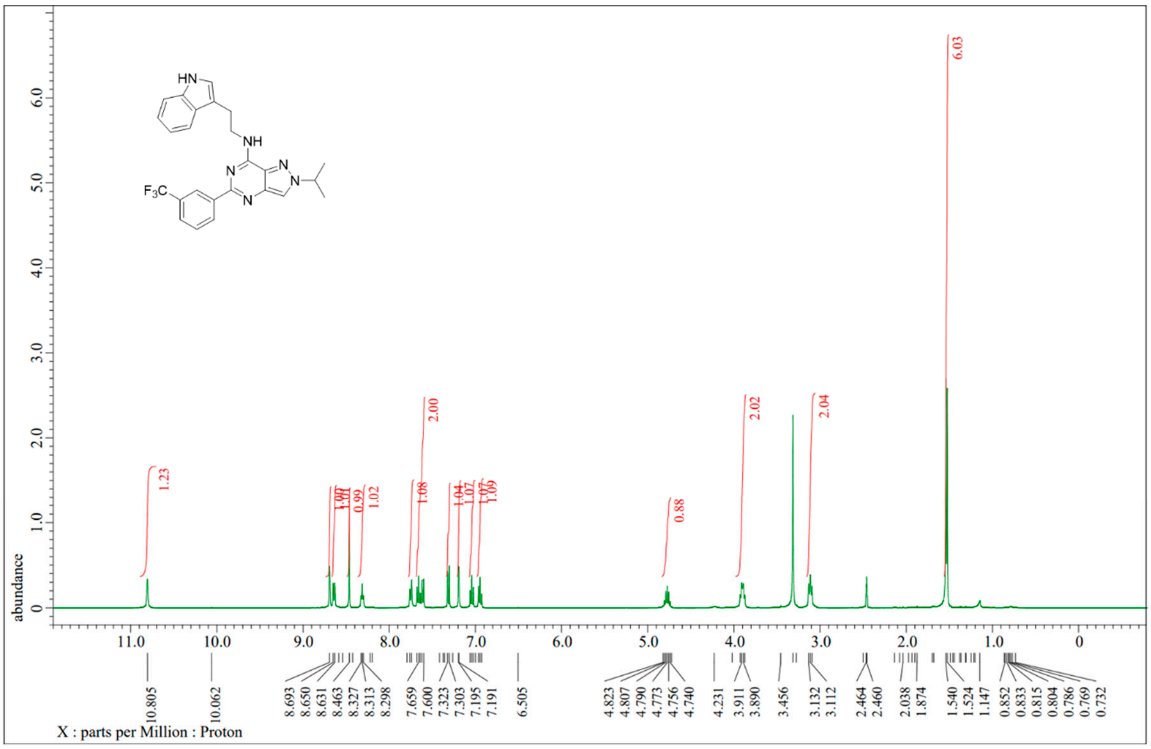

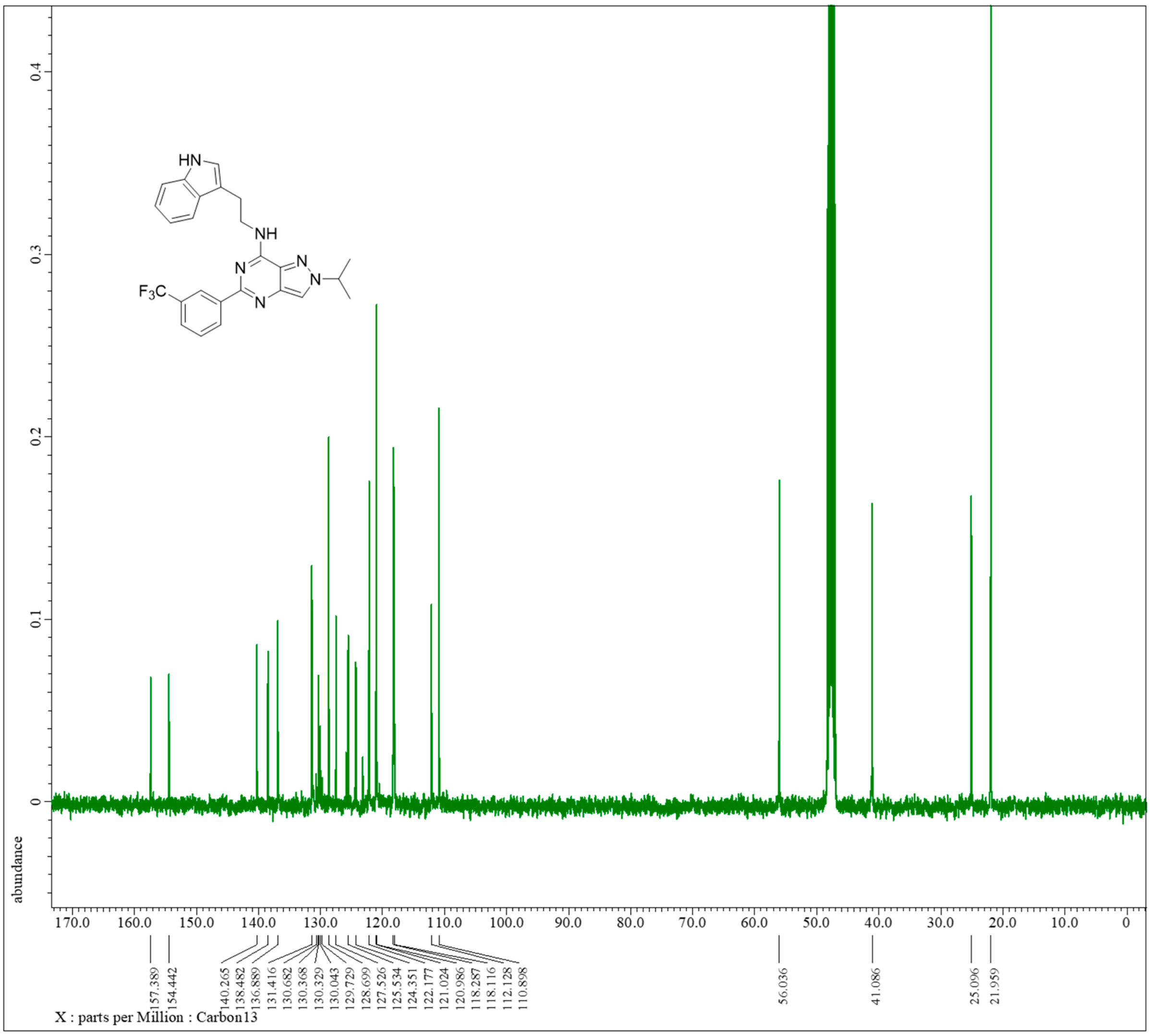
HN
NH
N
N
N
N
F_3C
abundance
0.4
0.3
0.2
0.1
0
170.0
160.0
150.0
140.0
130.0
120.0
110.0
100.0
90.0
80.0
70.0
60.0
50.0
40.0
30.0
20.0
10.0
0
157.389
154.442
140.265
138.482
136.889
131.416
130.682
130.368
130.329
130.043
129.729
128.699
127.526
125.534
124.351
122.177
121.024
120.986
118.287
118.116
112.128
110.898
56.036
41.086
25.096
21.959
X : parts per Million : Carbon13

***N*-(2-(1*H*-indol-3-yl)ethyl)-5-(3-aminophenyl)-2-isopropyl-2*H*-pyrazolo[4,3-*d*]pyrimidin-7-amine (7f)**

^{1}H NMR, ^{13}C NMR

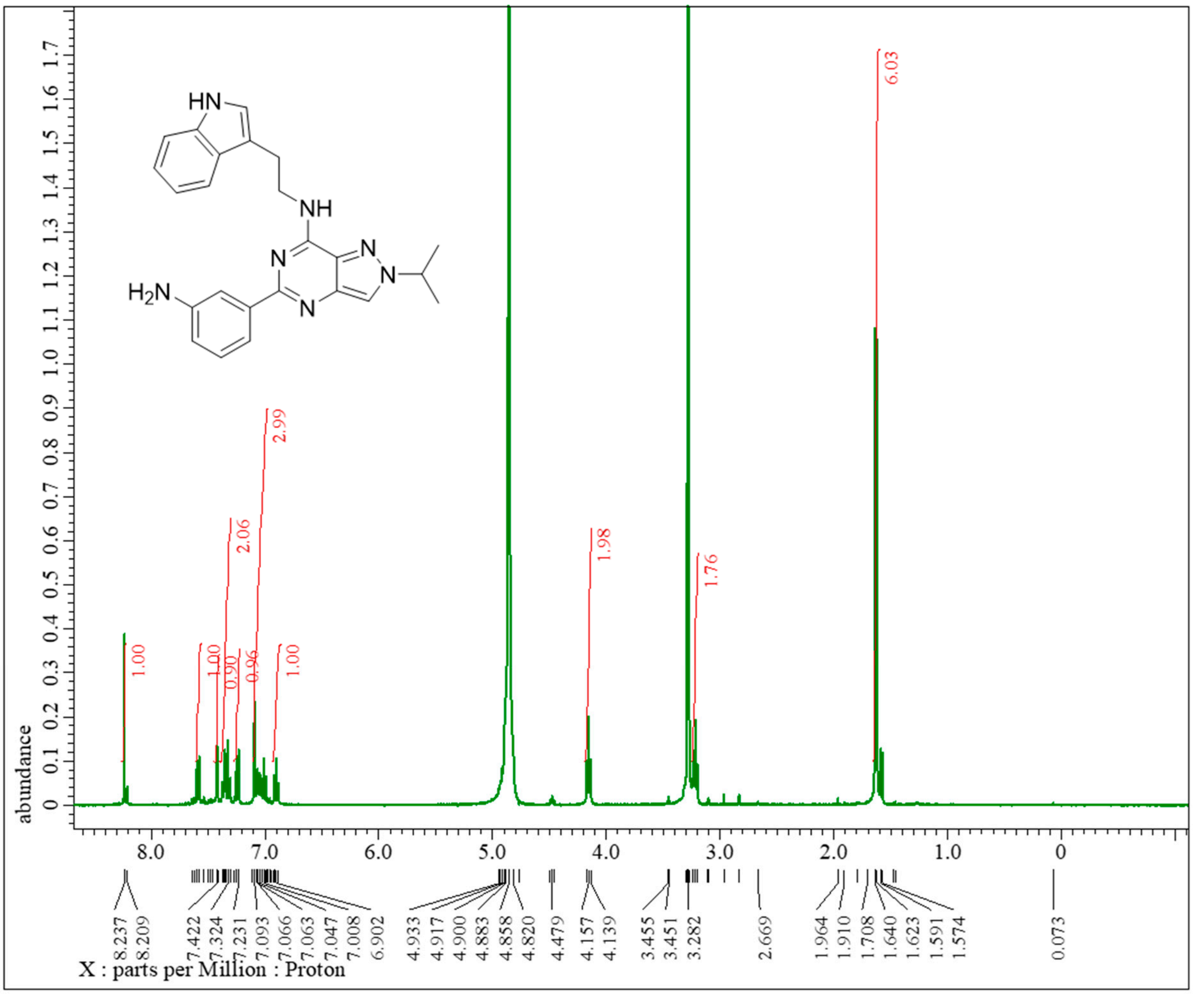

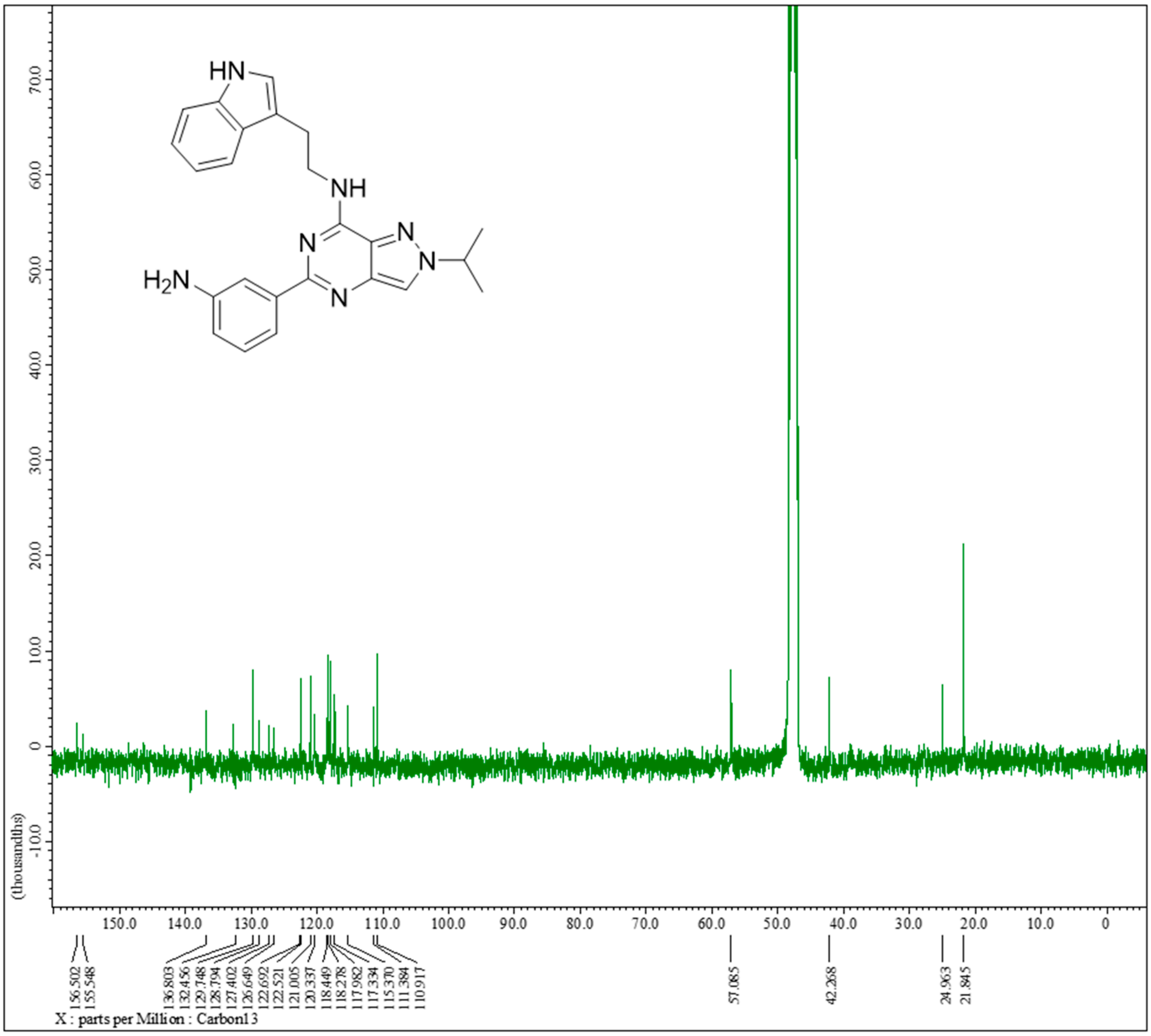
HN
NH
N
N
N
N
H_2N
(thousandths)
70.0
60.0
50.0
40.0
30.0
20.0
10.0
0
-10.0
150.0
140.0
130.0
120.0
110.0
100.0
90.0
80.0
70.0
60.0
50.0
40.0
30.0
20.0
10.0
0
156.502
155.548
136.803
132.456
129.748
128.794
127.402
126.649
122.692
122.521
121.005
120.337
118.449
118.278
117.982
117.334
115.370
111.384
110.917
57.085
42.268
24.963
21.845
X : parts per Million : Carbon13

3-(7-((2-(1*H*-indol-3-yl)ethyl)amino)-2-ethyl-2*H*-pyrazolo[4,3-*d*]pyrimidin-5-yl)benzonitrile (7g)

^{1}H NMR, ^{13}C NMR

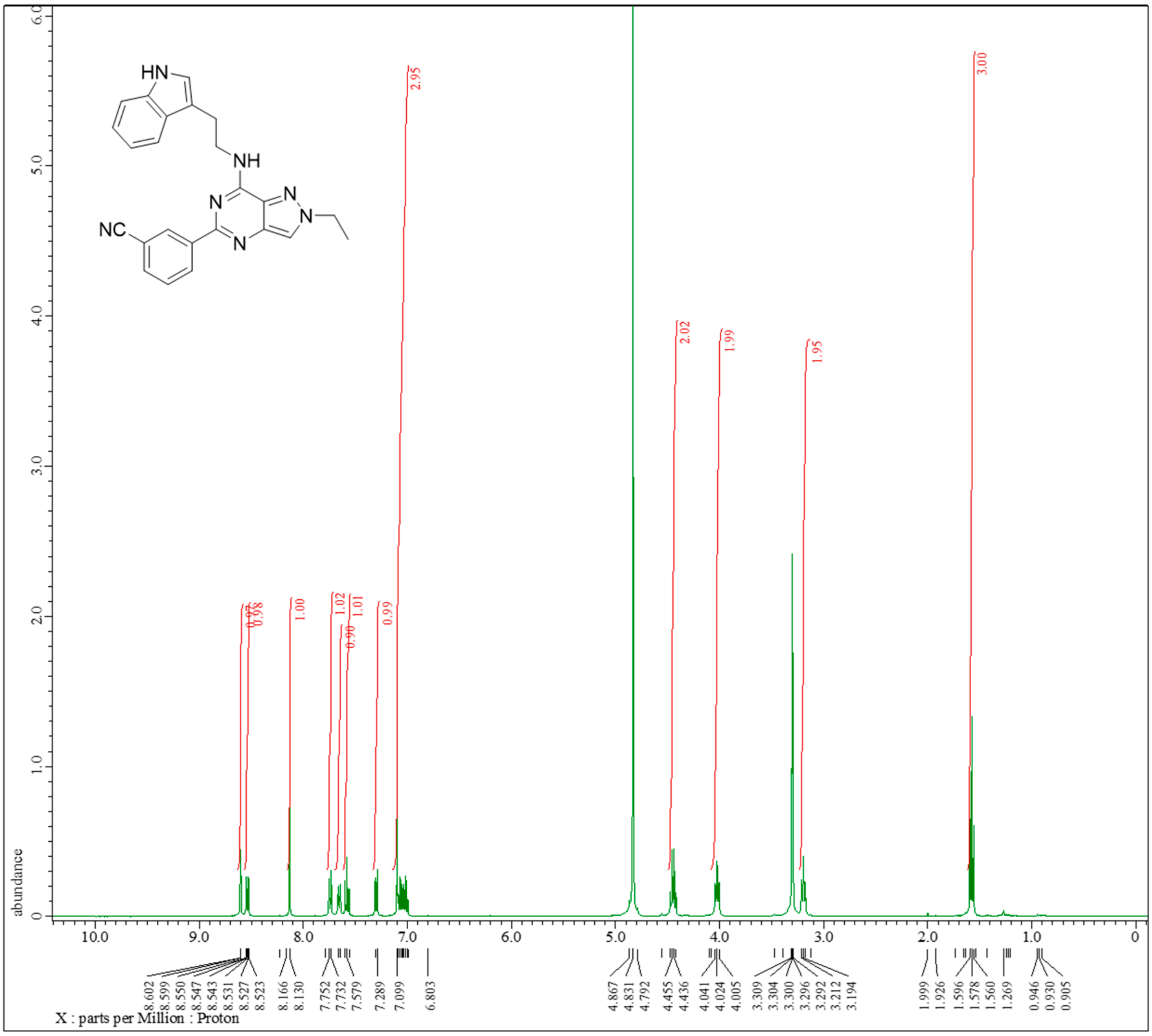

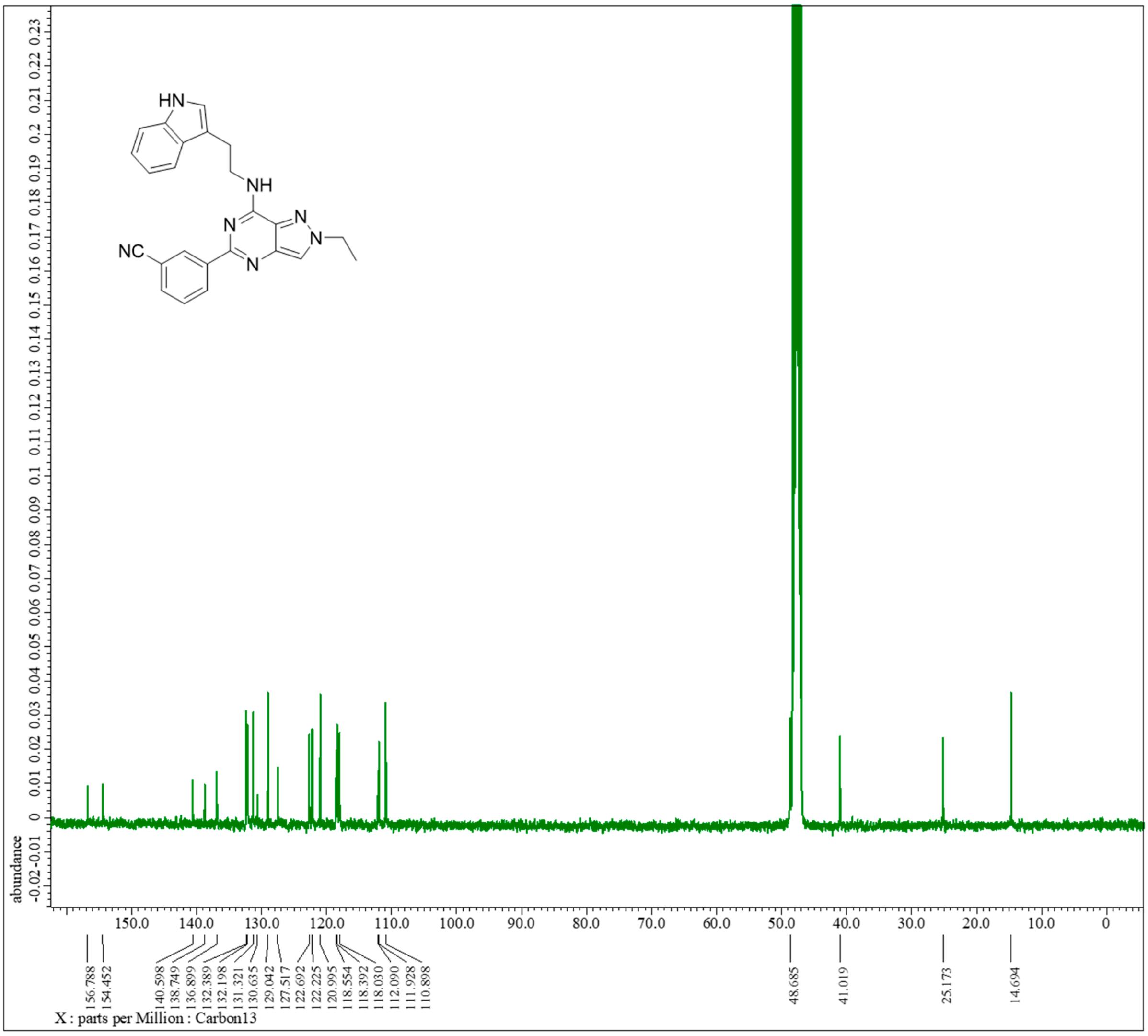
HN
NH
N
N
N
N
NC
abundance
X : parts per Million : Carbon13
156.788
154.452
140.598
138.749
136.899
132.389
132.198
131.321
130.635
129.042
127.517
122.692
122.225
120.995
118.554
118.392
118.030
112.090
111.928
110.898
48.685
41.019
25.173
14.694

3-(7-((2-(1*H*-indol-3-yl)ethyl)amino)-2-butyl-2*H*-pyrazolo[4,3-*d*]pyrimidin-5-yl)benzonitrile (7h) ^{1}H NMR, ^{13}C NMR

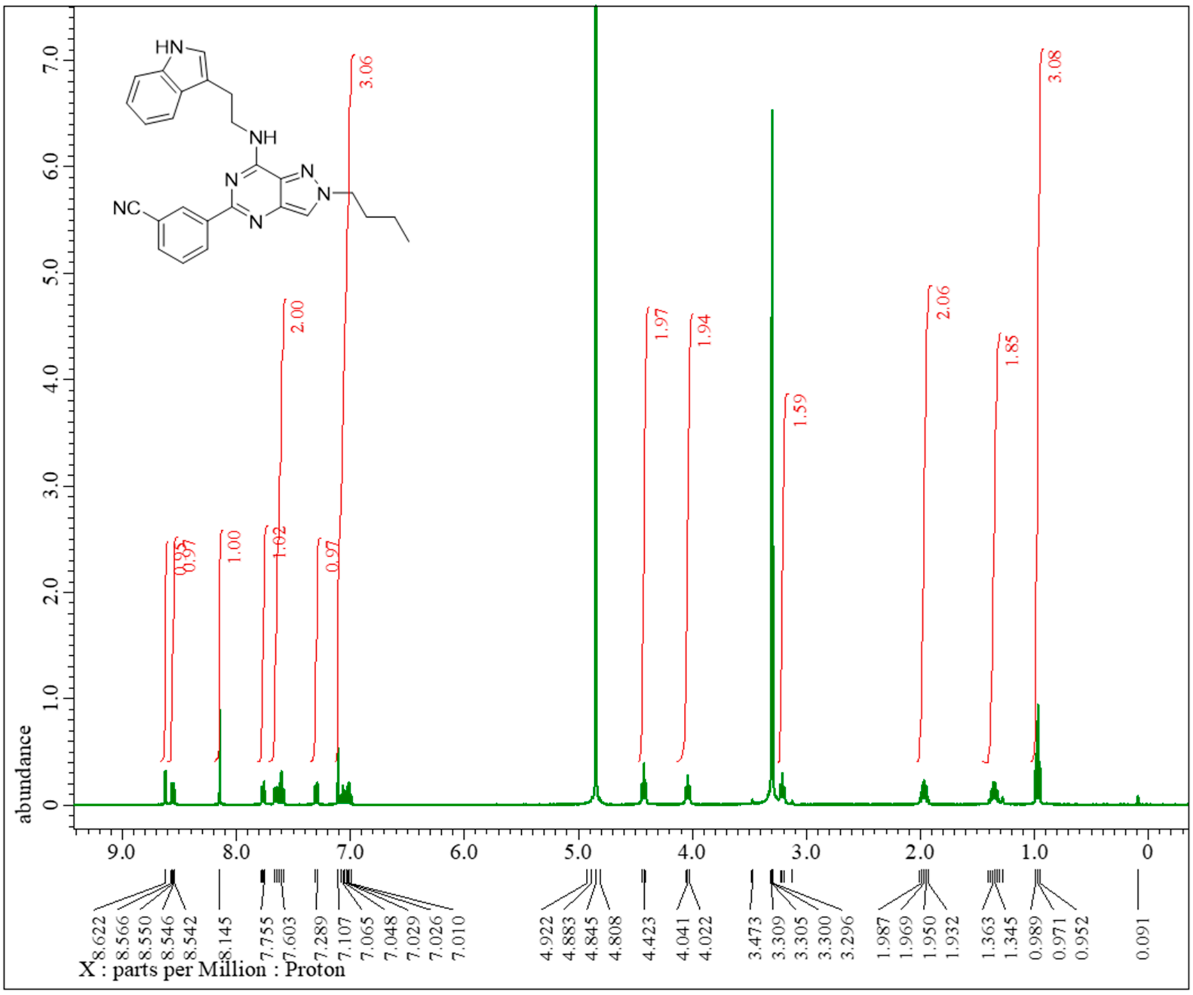

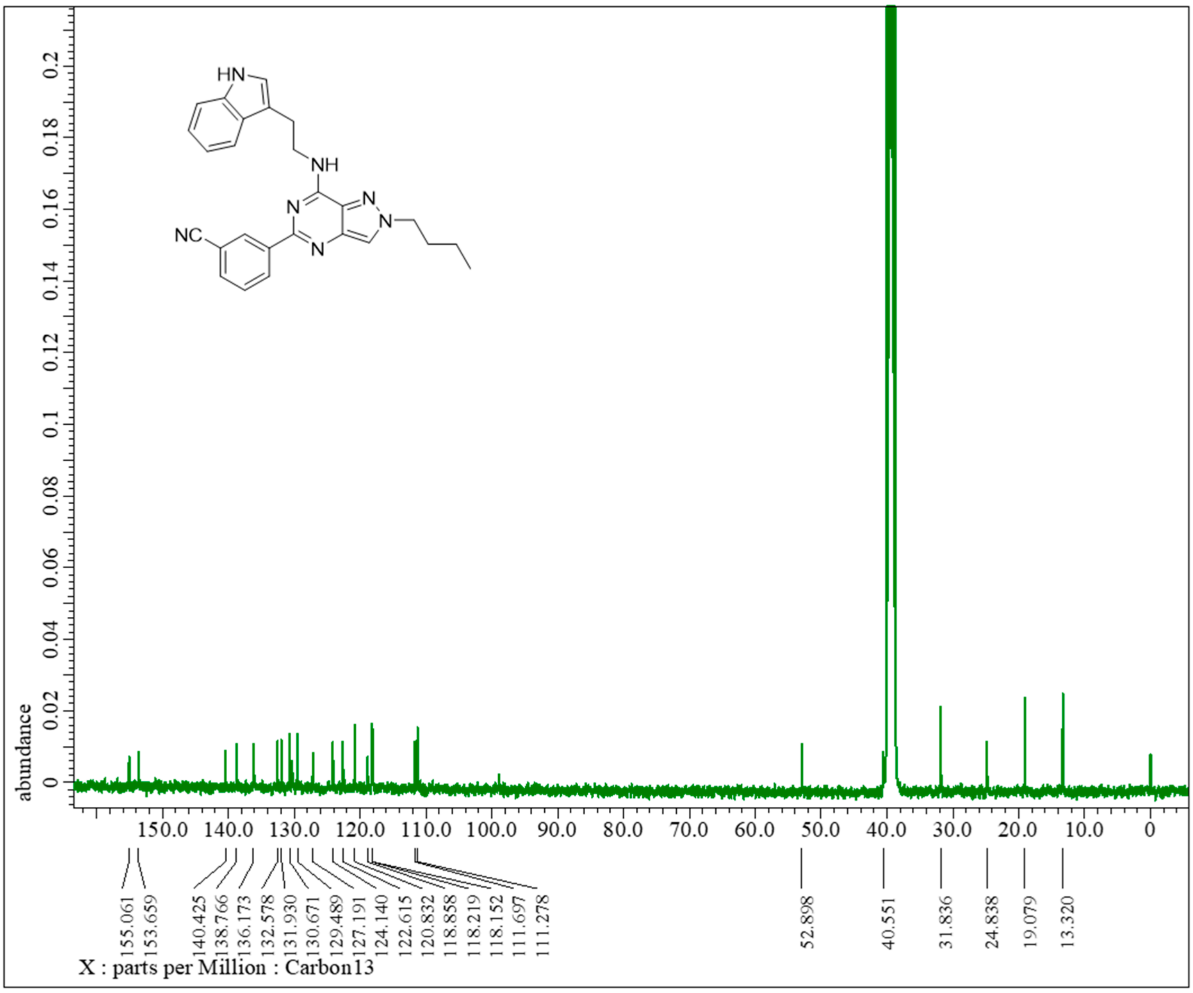

HN
NH
N
N
N
N
NC
abundance
0
0.02
0.04
0.06
0.08
0.1
0.12
0.14
0.16
0.18
0.2
150.0
140.0
130.0
120.0
110.0
100.0
90.0
80.0
70.0
60.0
50.0
40.0
30.0
20.0
10.0
0
155.061
153.659
140.425
138.766
136.173
132.578
131.930
130.671
129.489
127.191
124.140
122.615
120.832
118.858
118.219
118.152
111.697
111.278
52.898
40.551
31.836
24.838
19.079
13.320
X : parts per Million : Carbon13

3-(7-((2-(5-hydroxy-1*H*-indol-3-yl)ethyl)amino)-2-isopropyl-2*H*-pyrazolo[4,3-*d*]pyrimidin-5-yl)benzonitrile (7i)

^{1}H NMR, ^{13}C NMR

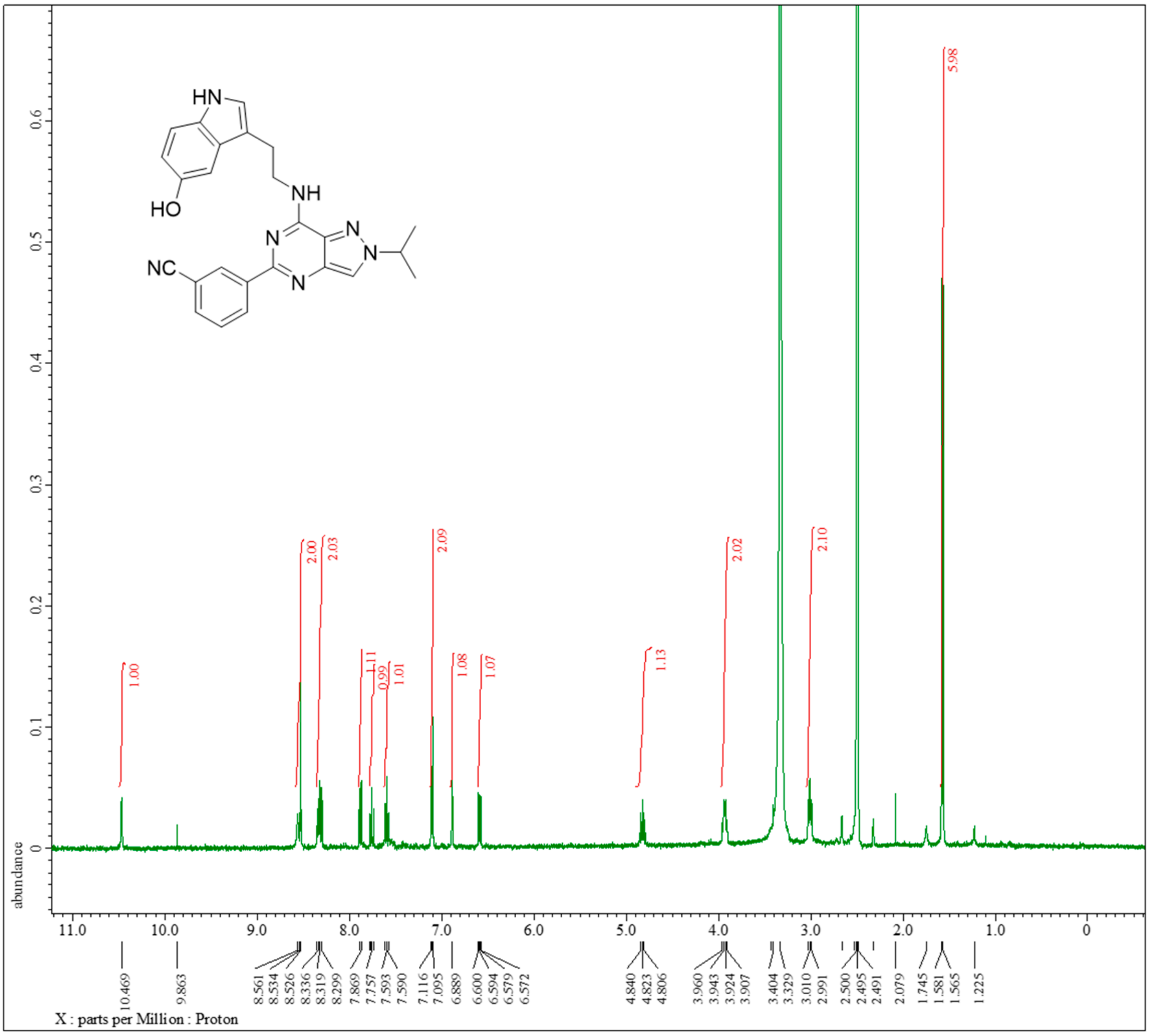

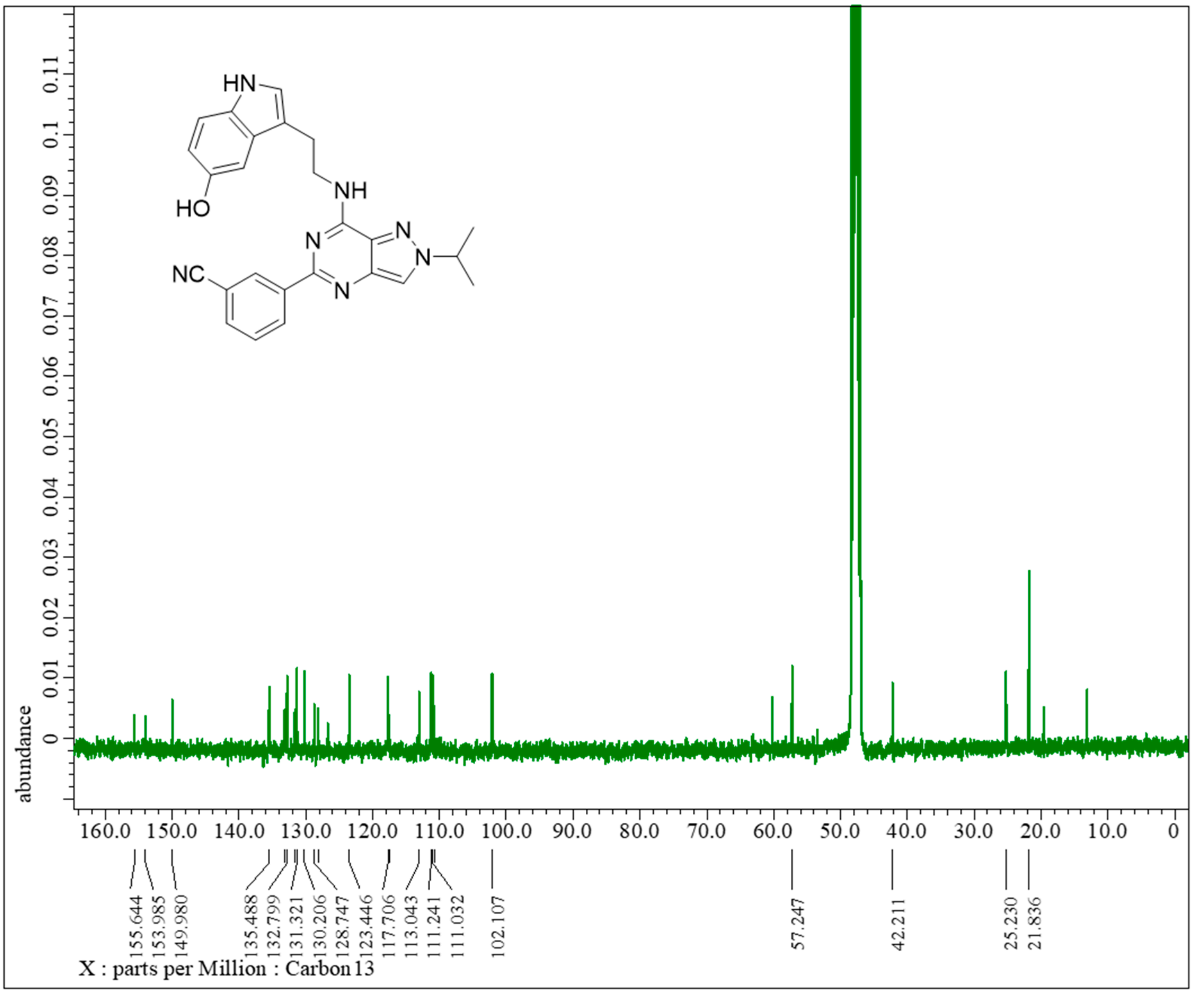
abundance
HN
HO
NH
N
N
N
N
NC
155.644
153.985
149.980
135.488
132.799
131.321
130.206
128.747
123.446
117.706
113.043
111.241
111.032
102.107
57.247
42.211
25.230
21.836
X : parts per Million : Carbon13

_N_-(2-(1_H_-indol-3-yl)ethyl)-5-(3-fluorophenyl)-2-isopropyl-2_H_-pyrazolo[4,3-_d_]pyrimidin-7-amine (7j)

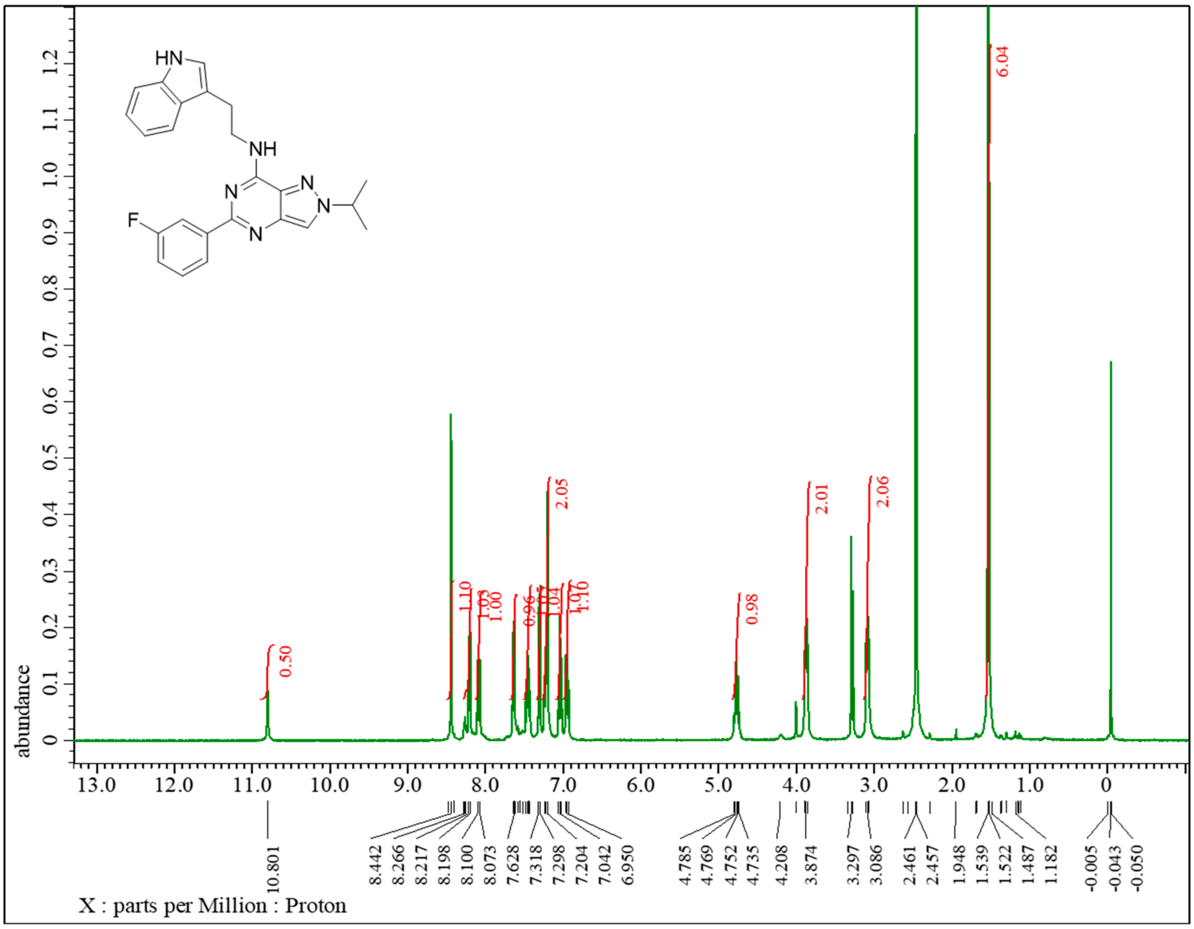

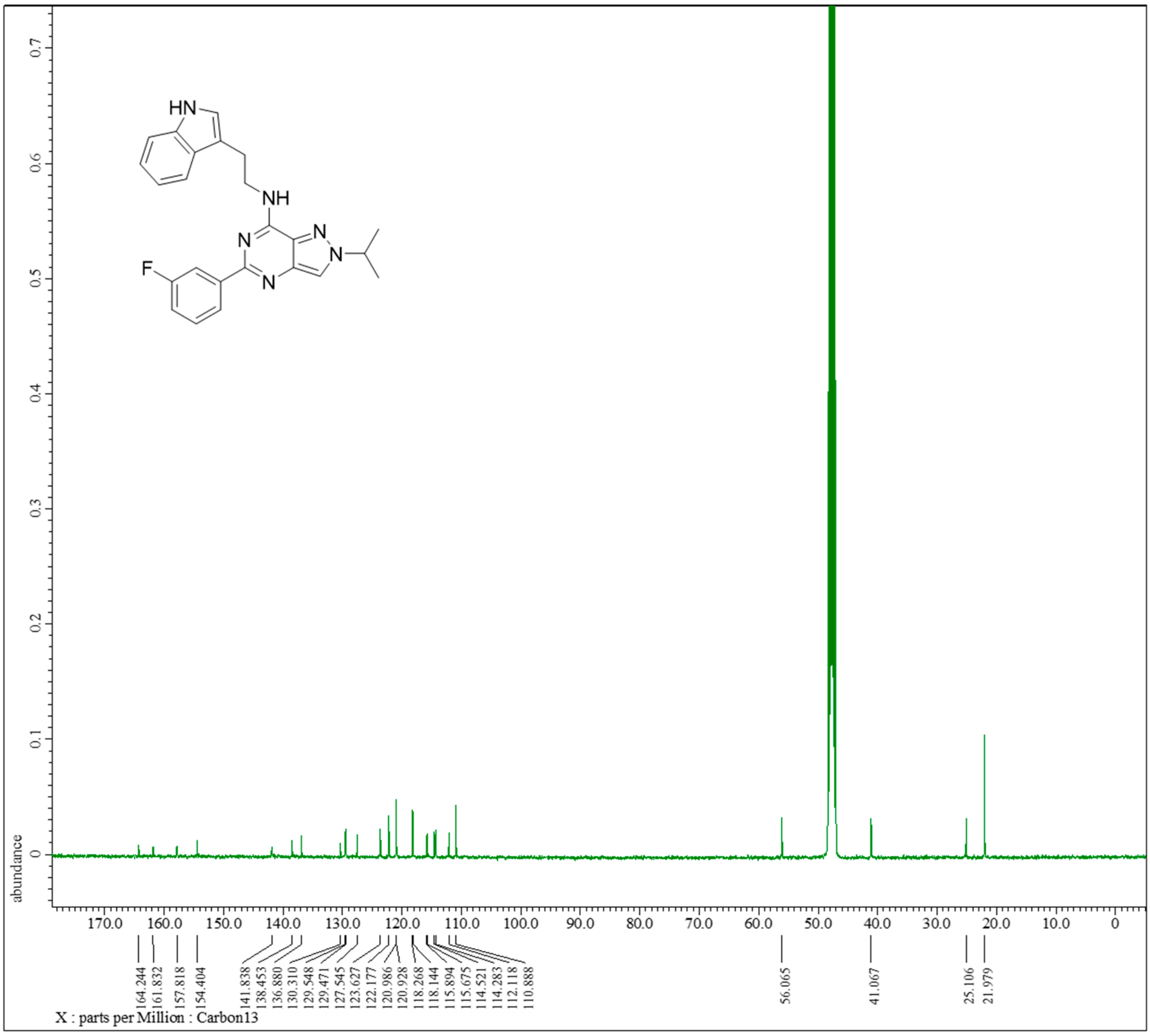
HN
NH
N
N
N
N
F
abundance
0
0.1
0.2
0.3
0.4
0.5
0.6
0.7
170.0
160.0
150.0
140.0
130.0
120.0
110.0
100.0
90.0
80.0
70.0
60.0
50.0
40.0
30.0
20.0
10.0
0
164.244
161.832
157.818
154.404
141.838
138.453
136.880
130.310
129.548
129.471
127.545
123.627
122.177
120.986
120.928
118.268
118.144
115.894
115.675
114.521
114.283
112.118
110.888
56.065
41.067
25.106
21.979
X : parts per Million : Carbon13

N-(2-(1H-indol-3-yl)ethyl)-5-(3,5-difluorophenyl)-2-isopropyl-2H-pyrazolo[4,3-d]pyrimidin-7-amine (7k)

^{1}H NMR, ^{13}C NMR

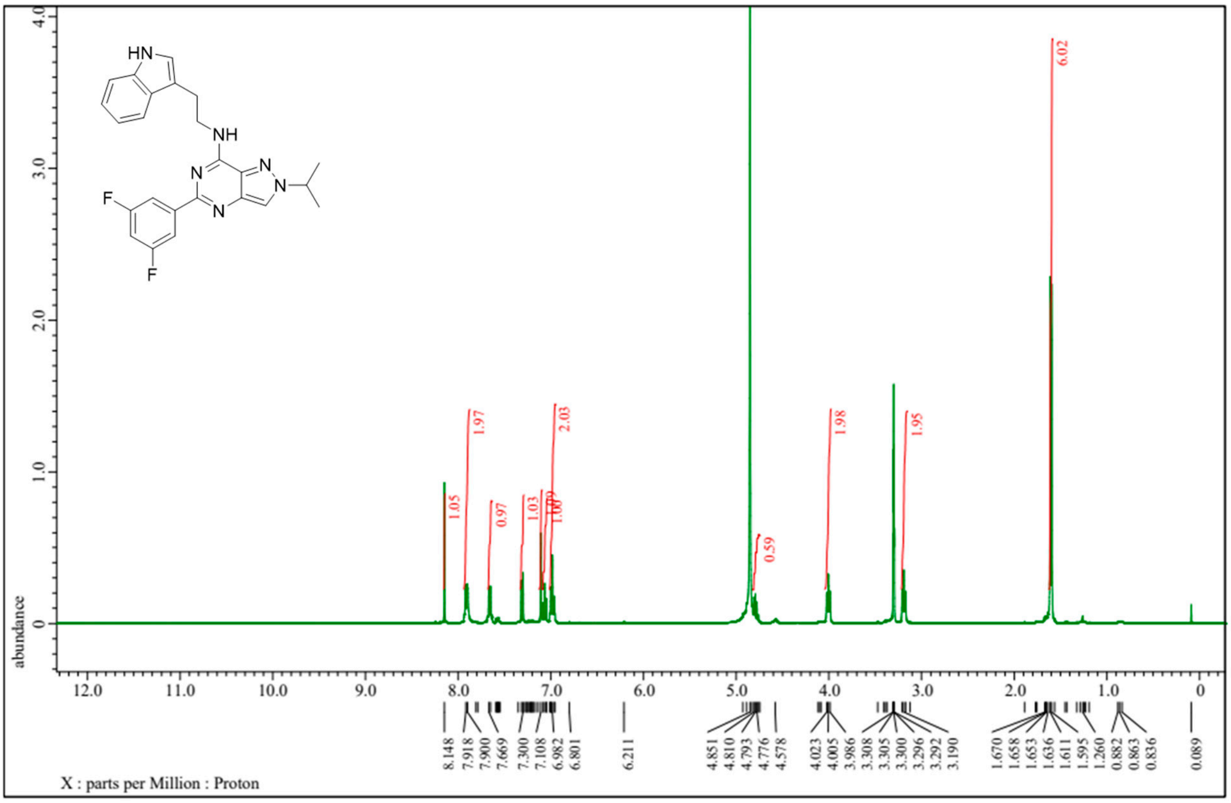

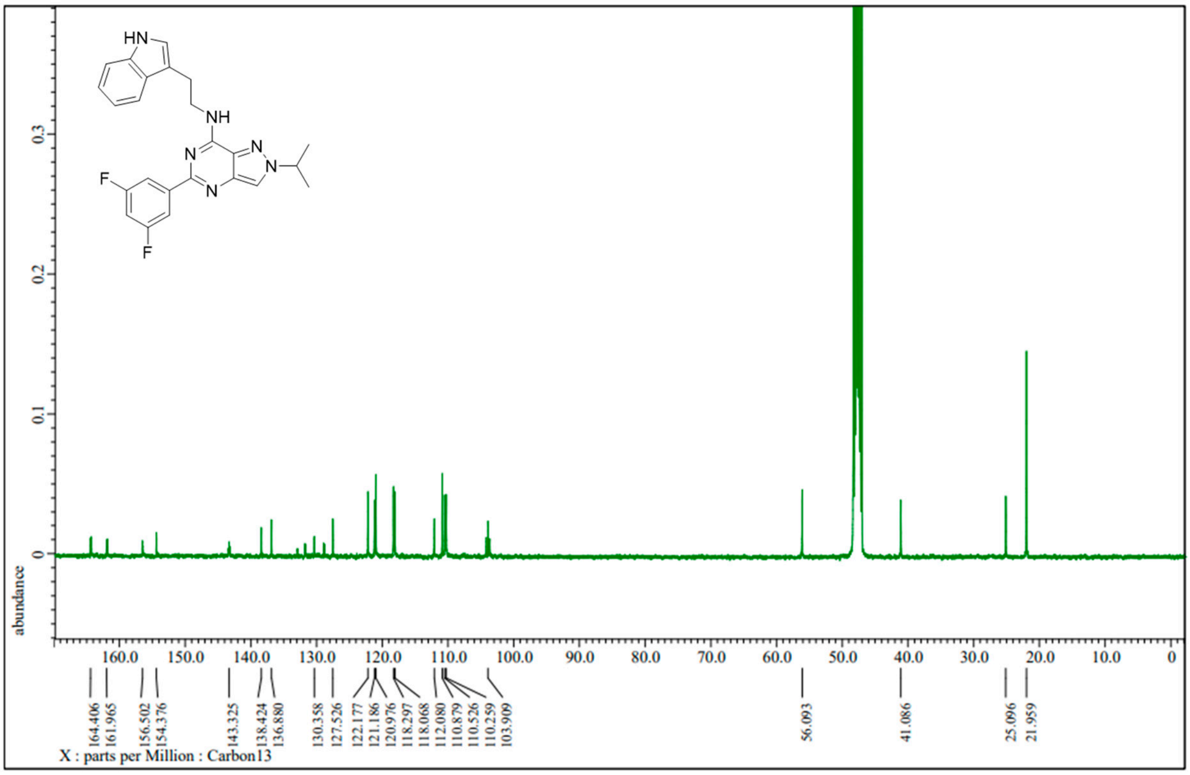
HN
NH
N
N
N
N
F
F
abundance
0
0.1
0.2
0.3
160.0
150.0
140.0
130.0
120.0
110.0
100.0
90.0
80.0
70.0
60.0
50.0
40.0
30.0
20.0
10.0
0
164.406
161.965
156.502
154.376
143.325
138.424
136.880
130.358
127.526
122.177
121.186
120.976
118.297
118.068
112.080
110.879
110.526
110.259
103.909
56.093
41.086
25.096
21.959
X : parts per Million : Carbon13

N-(2-(1*H*-indol-3-yl)ethyl)-2-isopropyl-5-(3-(trifluoromethoxy)phenyl)-2*H*-pyrazolo[4,3-d]pyrimidin-7-amine (7l)

^{1}H NMR, ^{13}C NMR

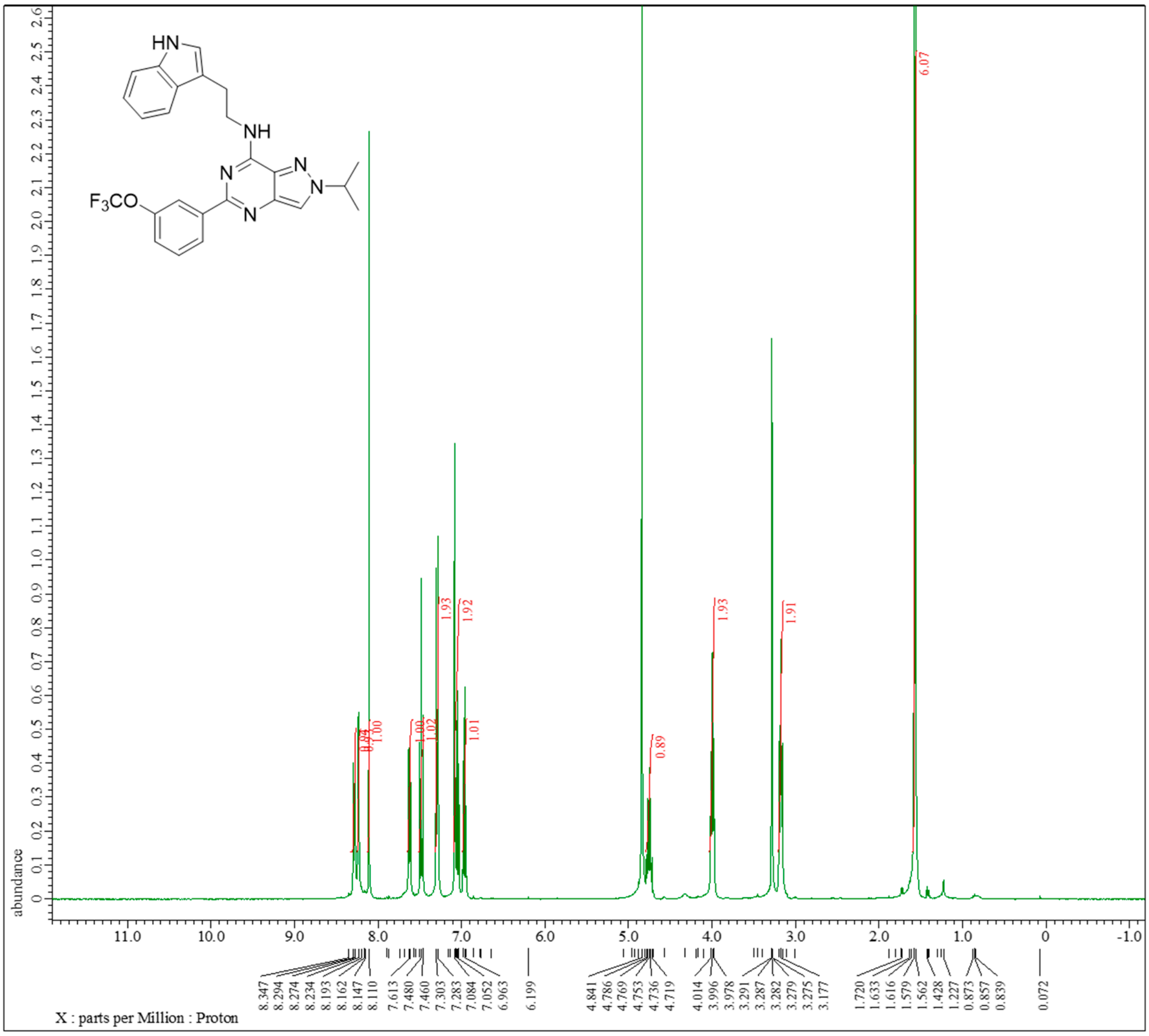

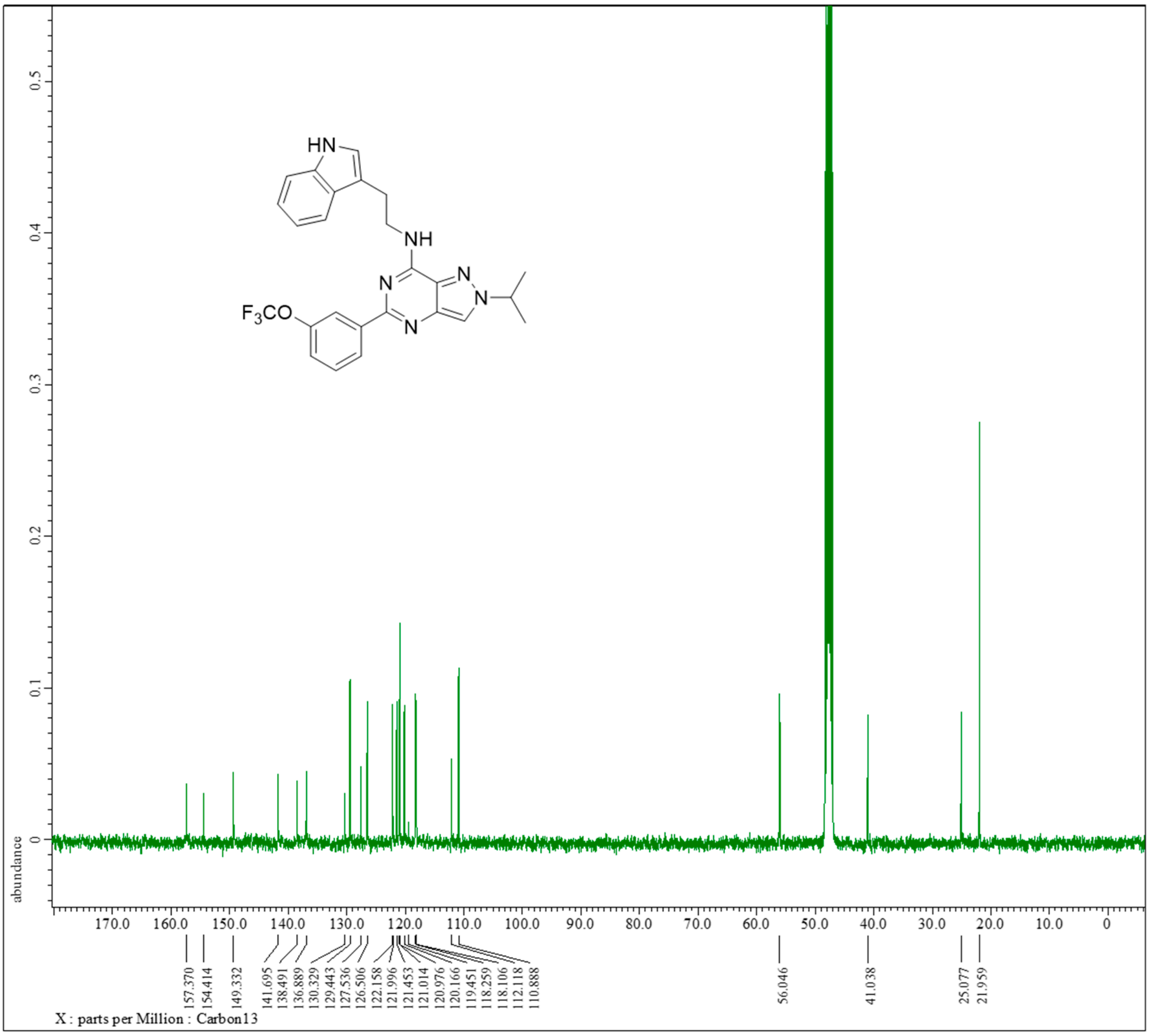
HN
NH
N
N
N
N
F_3CO
abundance
0
0.1
0.2
0.3
0.4
0.5
170.0
160.0
150.0
140.0
130.0
120.0
110.0
100.0
90.0
80.0
70.0
60.0
50.0
40.0
30.0
20.0
10.0
0
157.370
154.414
149.332
141.695
138.491
136.889
130.329
129.443
127.536
126.506
122.158
121.996
121.453
121.014
120.976
120.166
119.451
118.259
118.106
112.118
110.888
56.046
41.038
25.077
21.959
X : parts per Million : Carbon13

tert-butyl-3-(7-((2-(1*H*-indol-3-yl)ethyl)amino)-2-isopropyl-2*H*-pyrazolo[4,3-*d*]pyrimidin-5-yl)benzoate (7m)

^{1}H NMR, ^{13}C NMR

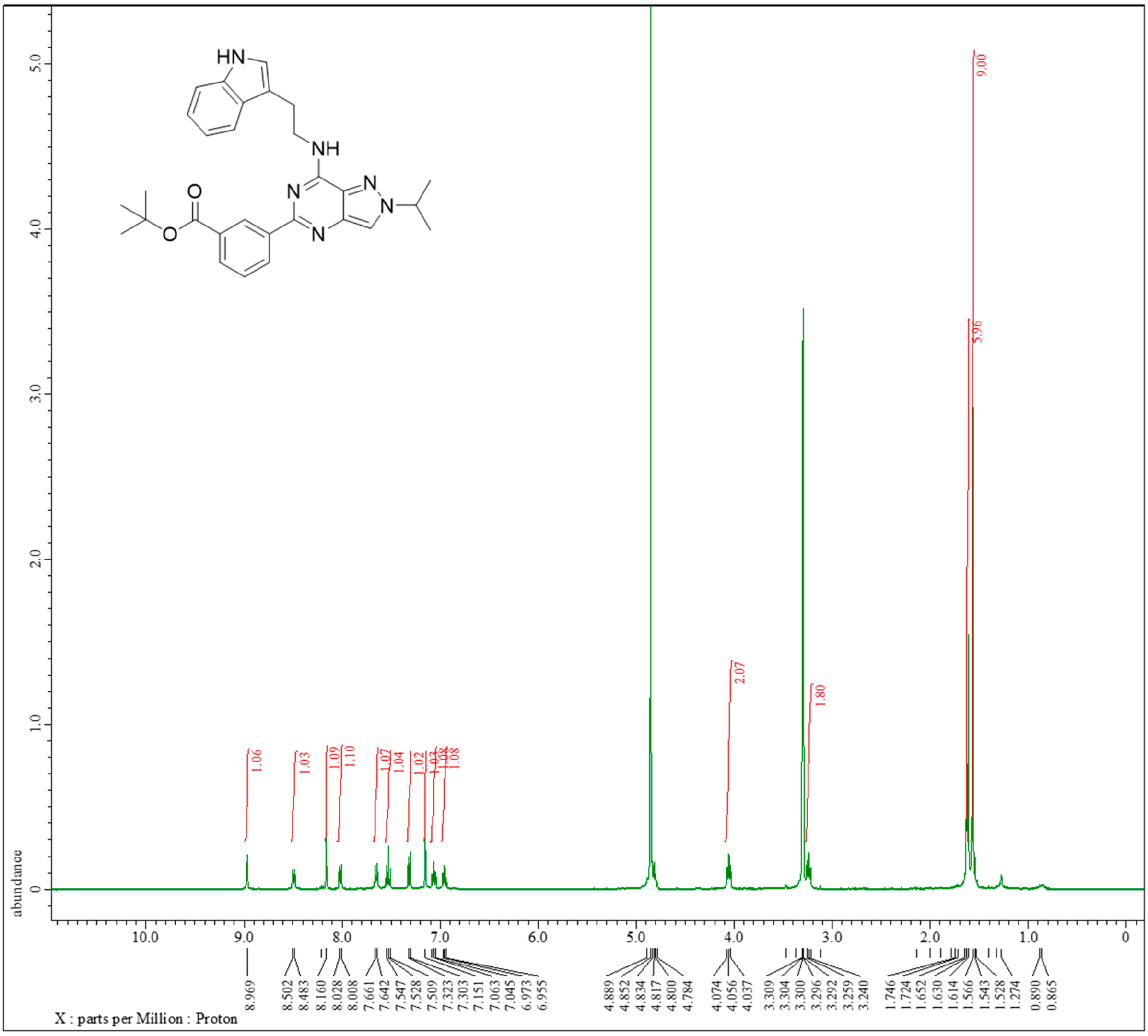

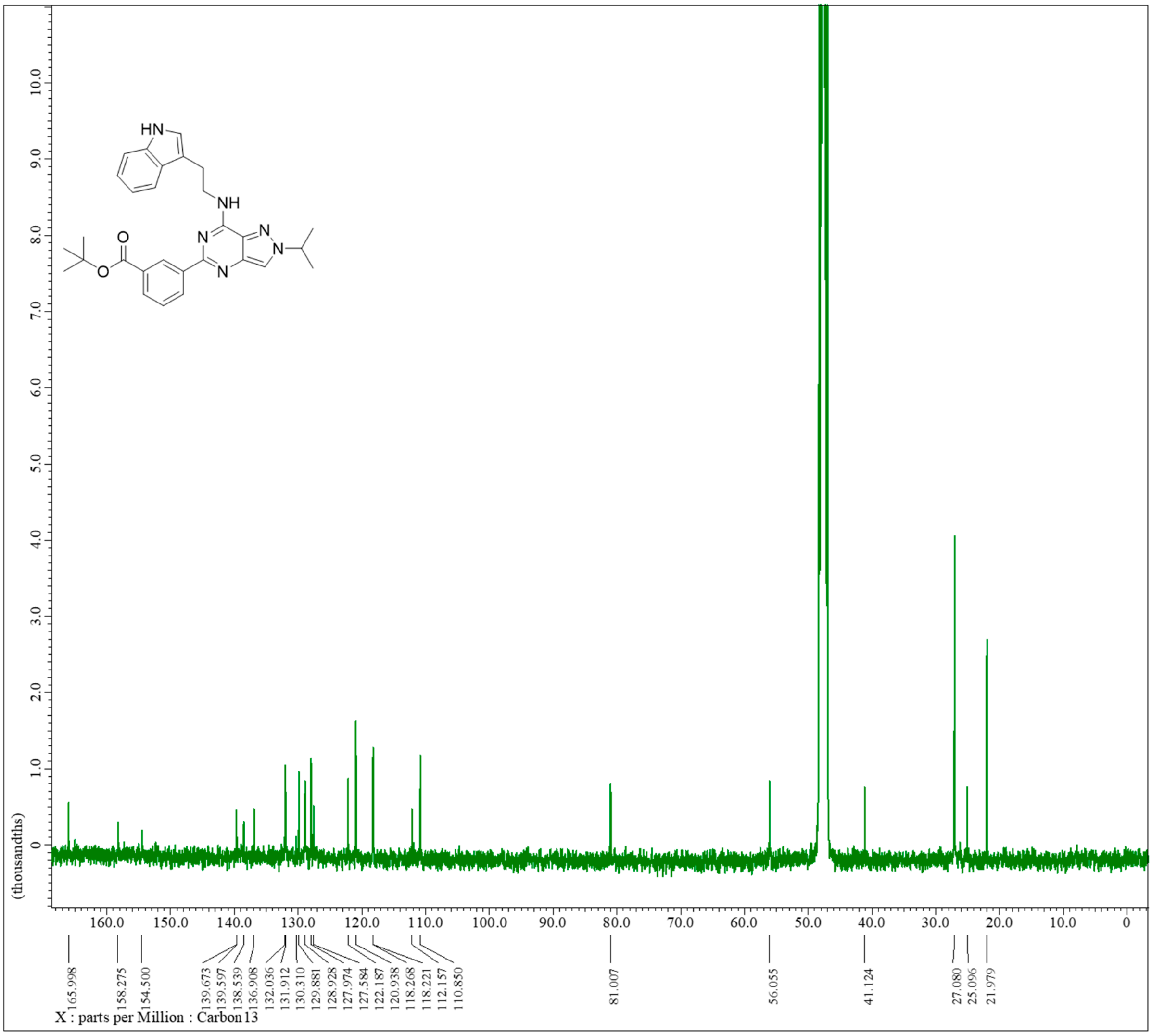
HN
NH
N
N
N
N
O
O
(thousandths)
0
1.0
2.0
3.0
4.0
5.0
6.0
7.0
8.0
9.0
10.0
160.0
150.0
140.0
130.0
120.0
110.0
100.0
90.0
80.0
70.0
60.0
50.0
40.0
30.0
20.0
10.0
0
165.998
158.275
154.500
139.673
139.597
138.539
136.908
132.036
131.912
130.310
129.881
128.928
127.974
127.584
122.187
120.938
118.268
118.221
112.157
110.850
81.007
56.055
41.124
27.080
25.096
21.979
X : parts per Million : Carbon13

HRMS data

N-(2-(1*H*-indol-3-yl)ethyl)-2-isopropyl-5-phenyl-2*H*-pyrazolo[4,3-*d*]pyrimidin-7-amine (7a)

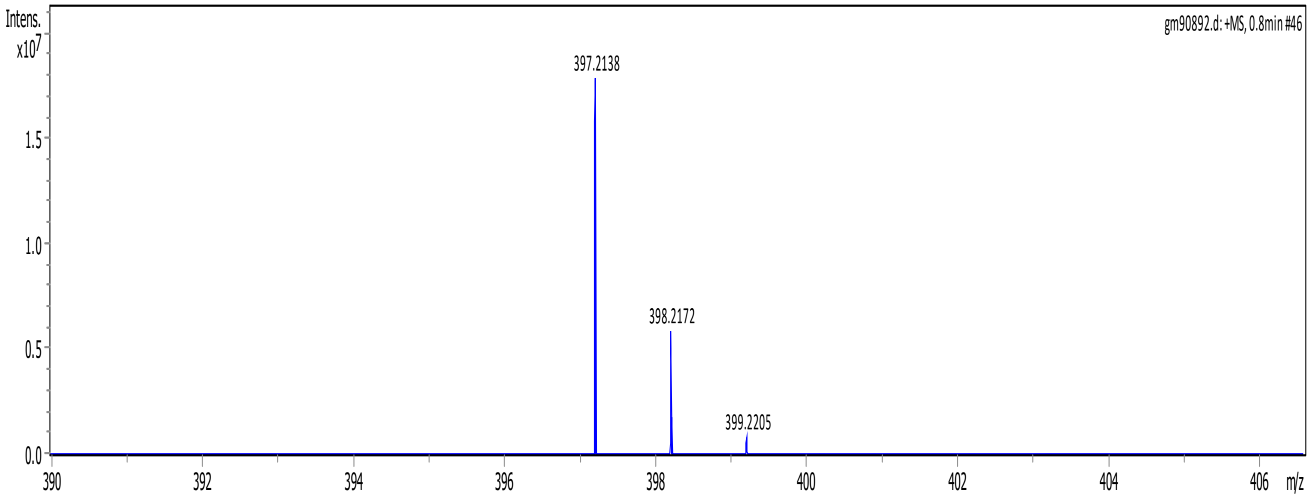

4-(7-((2-(1*H*-indol-3-yl)ethyl)amino)-2-isopropyl-2*H*-pyrazolo[4,3-*d*]pyrimidin-5-yl)benzonitrile (7b)

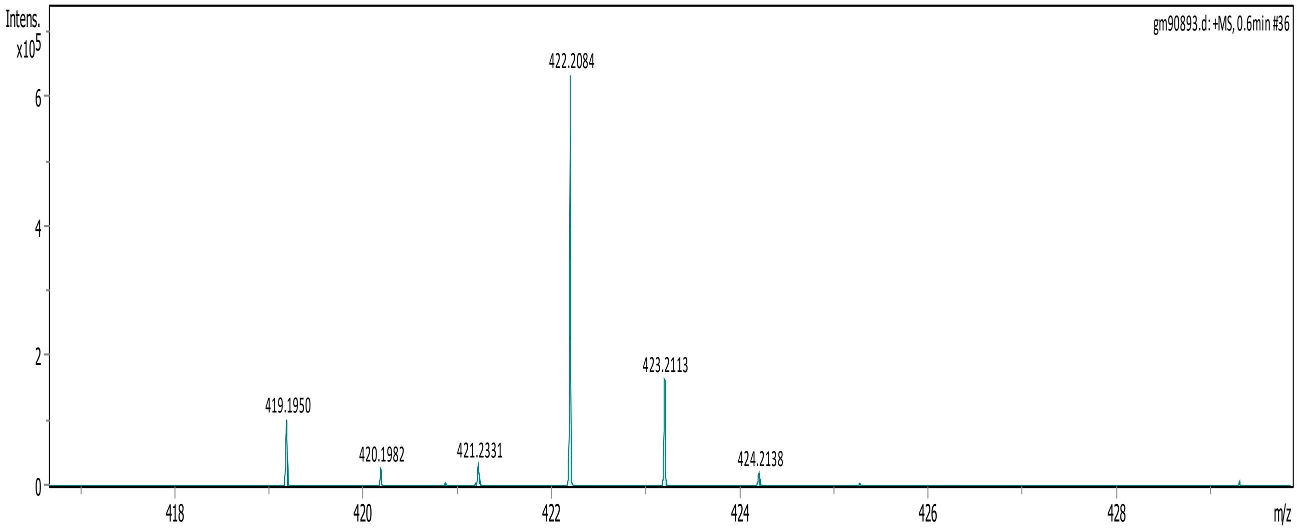

3-(7-((2-(1*H*-indol-3-yl)ethyl)amino)-2-isopropyl-2*H*-pyrazolo[4,3-*d*]pyrimidin-5-yl)benzonitrile (7c)

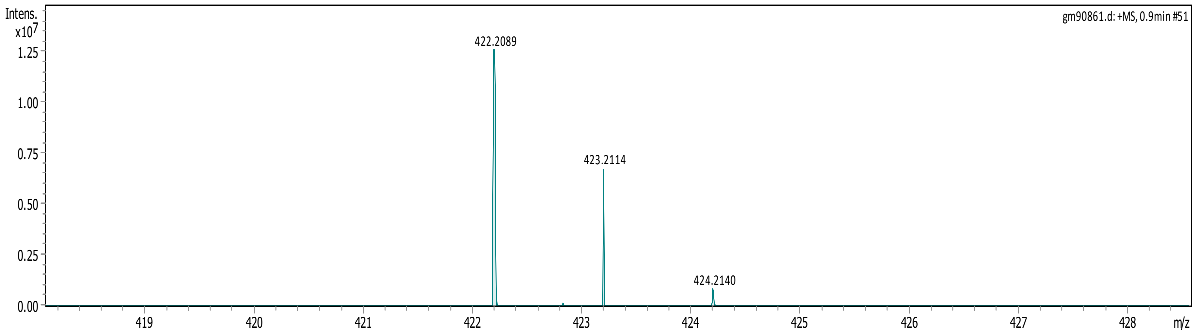

1-(3-(7-((2-(1*H*-indol-3-yl)ethyl)amino)-2-isopropyl-2*H*-pyrazolo[4,3-*d*]pyrimidin-5-yl)phenyl)ethan-1-one (7d)

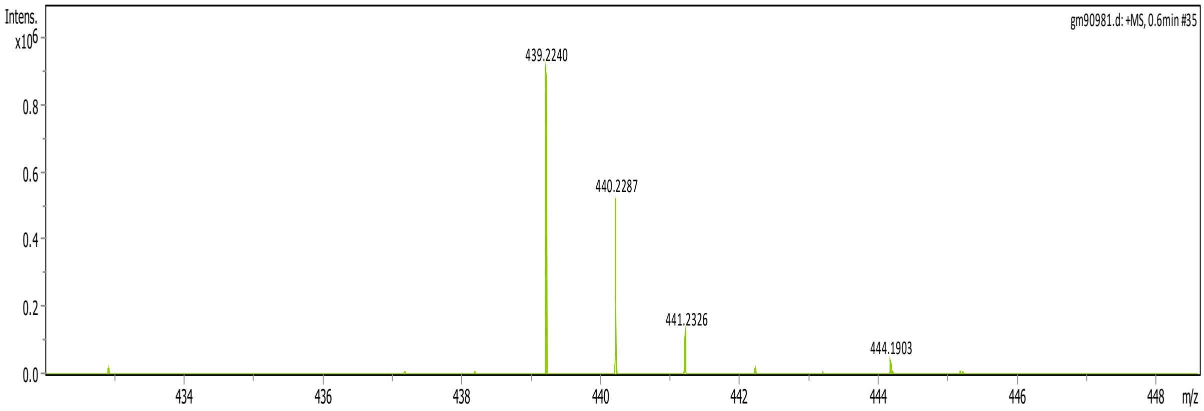

***N*-(2-(1*H*-indol-3-yl)ethyl)-2-isopropyl-5-(3-(trifluoromethyl)phenyl)-2*H*-pyrazolo[4,3-*d*]pyrimidin-7-amine (7e)**

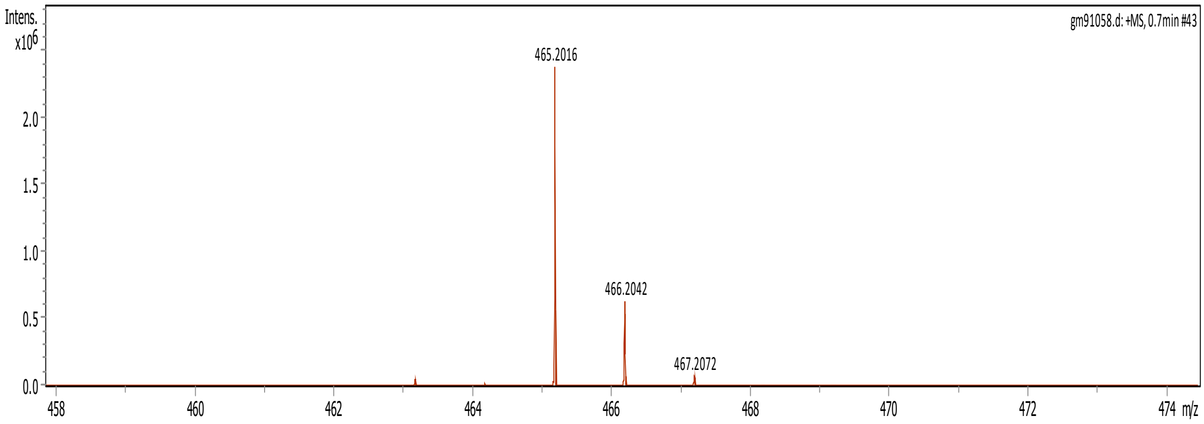

N-(2-(1H-indol-3-yl)ethyl)-5-(3-aminophenyl)-2-isopropyl-2H-pyrazolo[4,3-d]pyrimidin-7-amine (7f)

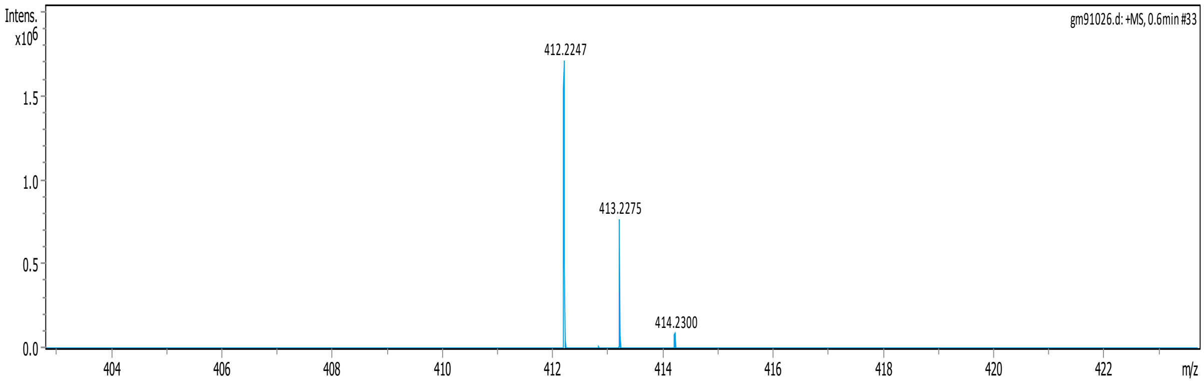

3-(7-((2-(1H-indol-3-yl)ethyl)amino)-2-ethyl-2H-pyrazolo[4,3-d]pyrimidin-5-yl)benzonitrile (7g)

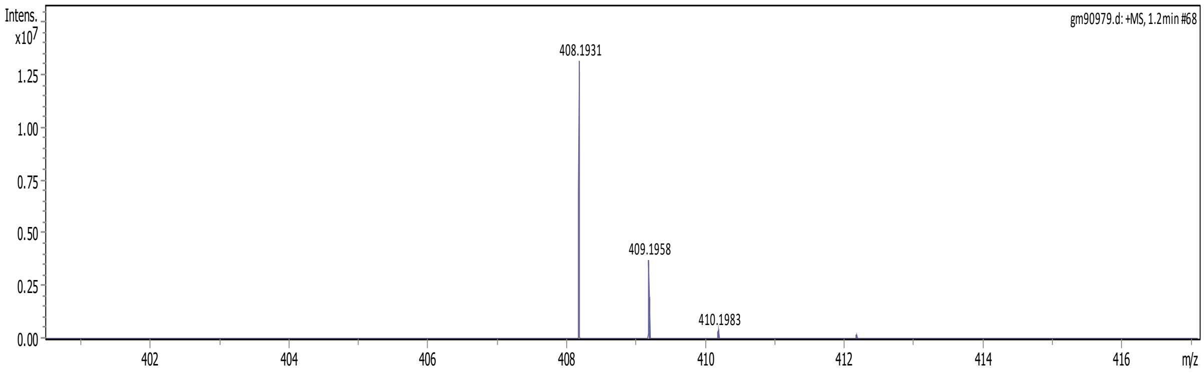

3-(7-((2-(1H-indol-3-yl)ethyl)amino)-2-butyl-2H-pyrazolo[4,3-d]pyrimidin-5-yl)benzonitrile (7h)

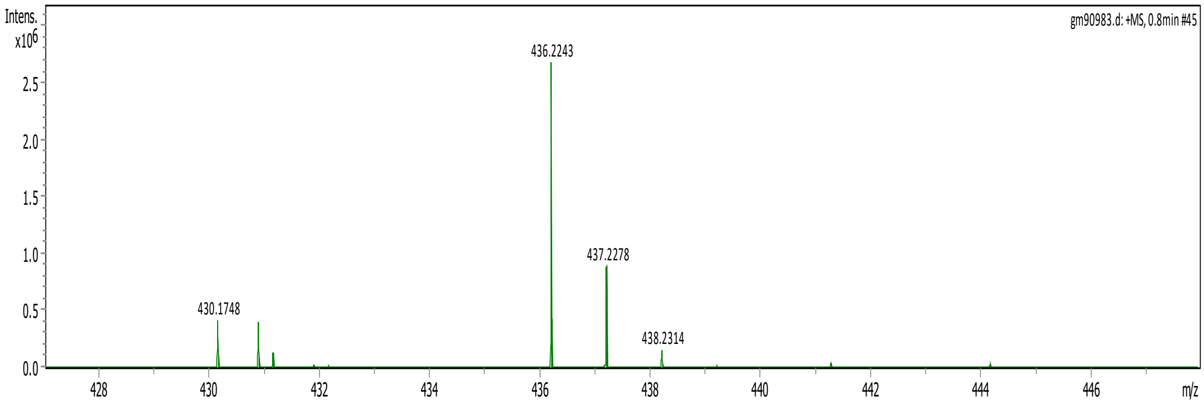

3-(7-((2-(5-hydroxy-1H-indol-3-yl)ethyl)amino)-2-isopropyl-2H-pyrazolo[4,3-d]pyrimidin-5-yl)benzonitrile (7i)

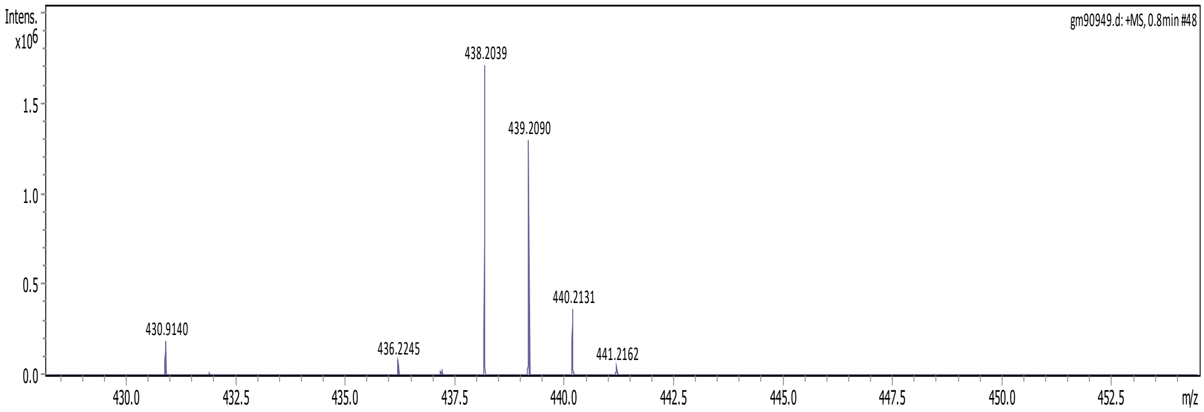

N-(2-(1H-indol-3-yl)ethyl)-5-(3-fluorophenyl)-2-isopropyl-2H-pyrazolo[4,3-d]pyrimidin-7-amine (7j)

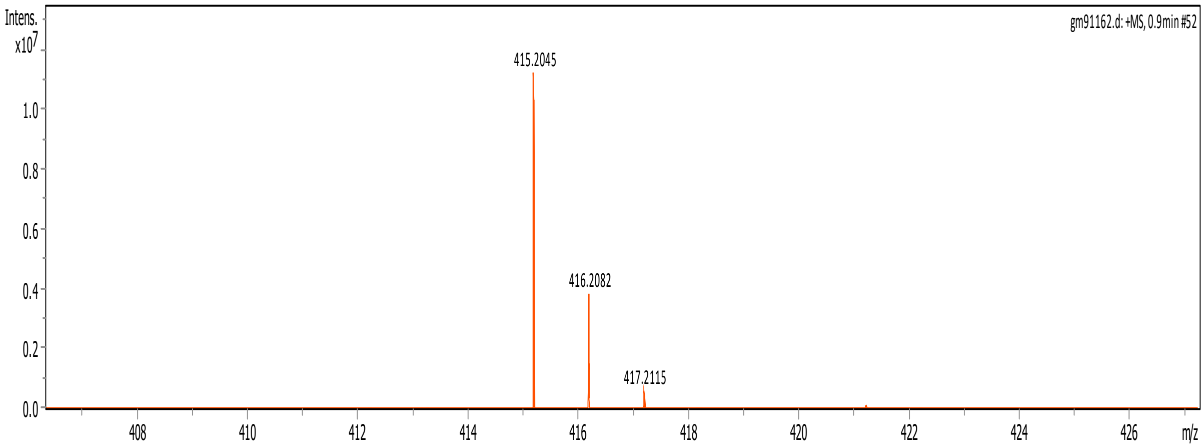

N-(2-(1H-indol-3-yl)ethyl)-5-(3,5-difluorophenyl)-2-isopropyl-2H-pyrazolo[4,3-d]pyrimidin-7-amine (7k)

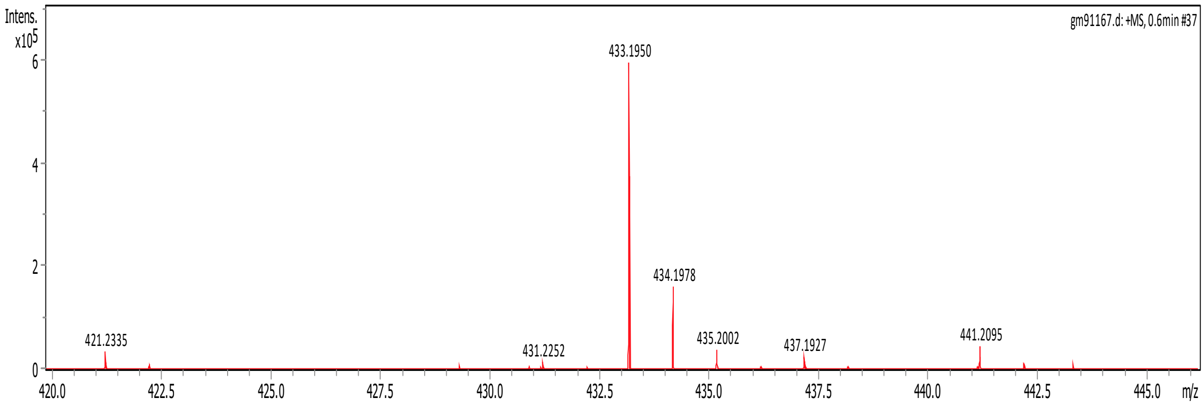

N-(2-(1H-indol-3-yl)ethyl)-2-isopropyl-5-(3-(trifluoromethoxy)phenyl)-2H-pyrazolo[4,3-d]pyrimidin-7-amine (7l)

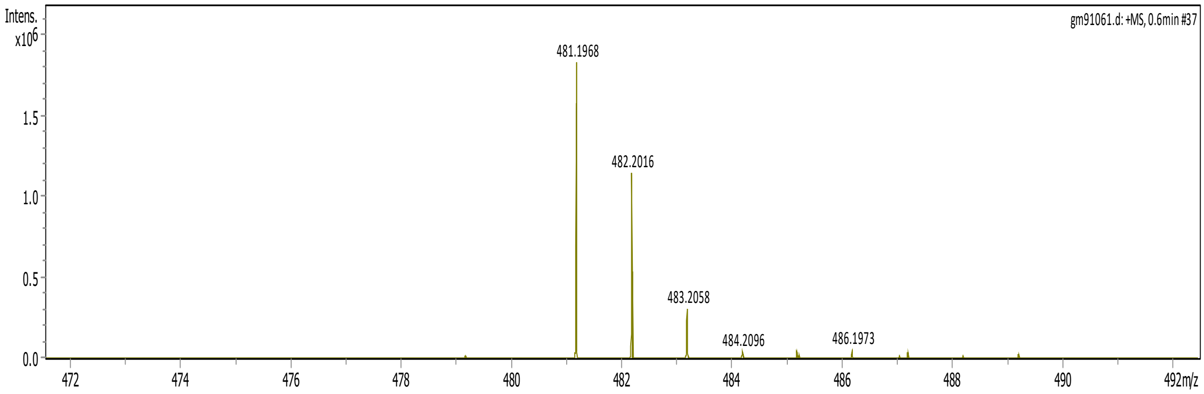

tert-butyl-3-(7-((2-(1*H*-indol-3-yl)ethyl)amino)-2-isopropyl-2*H*-pyrazolo[4,3-*d*]pyrimidin-5-yl)benzoate (7m)

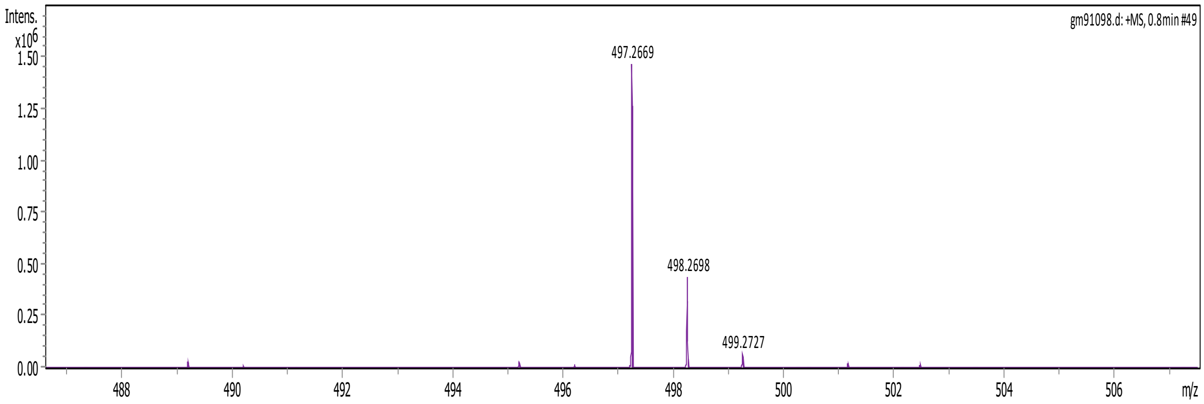

Supplement: Supplementary file 1 [file pharmaceutics-17-01359-s001.zip › pharmaceutics-3895560-supplementary.pdf]
